# Differentiating the roles of *Mycobacterium tuberculosis* substrate binding proteins, FecB and FecB2, in iron uptake

**DOI:** 10.1371/journal.ppat.1011650

**Published:** 2023-09-25

**Authors:** Rodger de Miranda, Bonnie J. Cuthbert, Thaís Klevorn, Alex Chao, Jessica Mendoza, Mark Arbing, Paul J. Sieminski, Kadamba Papavinasasundaram, Sumer Abdul-Hafiz, Sum Chan, Christopher M. Sassetti, Sabine Ehrt, Celia W. Goulding

**Affiliations:** 1 Department of Molecular Biology & Biochemistry, University of California Irvine, Irvine, California, United States of America; 2 Department of Microbiology and Immunology, Weill Cornell Medical College, New York, New York, United States of America; 3 UCLA-DOE Institute, UCLA, Los Angeles, Calofornia, United States of America; 4 Department of Microbiology and Physiological Systems, UMass Chan Medical School, Worcester, Massachusetts, United States of America; 5 Department of Pharmaceutical Sciences, University of California Irvine, Irvine, Califiornia, United States of America; McGill University, CANADA

## Abstract

*Mycobacterium tuberculosis* (Mtb), the causative agent of tuberculosis, poses a great threat to human health. With the emergence of drug resistant Mtb strains, new therapeutics are desperately needed. As iron is critical to the growth and survival of Mtb, mechanisms through which Mtb acquires host iron represent attractive therapeutic targets. Mtb scavenges host iron via Mtb siderophore-dependent and heme iron uptake pathways. While multiple studies describe the import of heme and ferric-siderophores and the export of apo-siderophores across the inner membrane, little is known about their transport across the periplasm and cell-wall environments. Mtb FecB and FecB2 are predicted periplasmic binding proteins implicated in host iron acquisition; however, their precise roles are not well understood. This study sought to differentiate the roles FecB and FecB2 play in Mtb iron acquisition. The crystallographic structures of Mtb FecB and FecB2 were determined to 2.0 Å and 2.2 Å resolution, respectively, and show distinct ligand binding pockets. *In vitro* ligand binding experiments for FecB and FecB2 were performed with heme and bacterial siderophores from Mtb and other species, revealing that both FecB and FecB2 bind heme, while only FecB binds the Mtb sideophore ferric-carboxymycobactin (Fe-cMB). Subsequent structure-guided mutagenesis of FecB identified a single glutamate residue—Glu339—that significantly contributes to Fe-cMB binding. A role for FecB in the Mtb siderophore-mediated iron acquisition pathway was corroborated by *Mycobacterium smegmatis* and Mtb pull-down assays, which revealed interactions between FecB and members of the mycobacterial siderophore export and import machinery. Similarly, pull-down assays with FecB2 confirms its role in heme uptake revealing interactions with a potential inner membrane heme importer. Due to ligand preference and protein partners, our data suggest that Mtb FecB plays a role in siderophore-dependent iron and heme acquisition pathways; in addition, we confirm that Mtb FecB2 is involved in heme uptake.

## Author summary

*Mycobacterium tuberculosis* (Mtb), the causative agent of tuberculosis, has long presented a threat to human health across the world and remains one of the leading infectious causes of death. Understanding how Mtb can survive and flourish in human hosts allows us to identify potential avenues for therapeutics. As iron is essential to Mtb survival, the mechanisms by which Mtb acquires iron from the host is a particularly attractive avenue. Here, we present evidence that two Mtb predicted periplasmic binding proteins, FecB and FecB2, are involved in iron uptake mechanisms. With ligand-binding experiments, we show that FecB2 specifically binds heme, while FecB can bind heme and the Mtb siderophore carboxymycobactin (cMB) in both its apo and iron bound forms. We also identify a FecB residue involved in ferric-cMB recognition using structure function analysis. Lastly, using co-immunoprecipitation, we identify FecB protein interaction partners that place FecB in the siderophore-dependent iron acquisition pathway and we confirm that Mtb FecB2 is involved in heme uptake. These experiments provide new and important information about a poorly understood, but essential Mtb survival mechanism, which ultimately may allow for novel anti-Mtb therapeutics.

## Introduction

Iron is an essential element for all forms of life as it is involved in respiration, electron transfer, energy generation and a variety of biological processes [1–4]. Bacterial pathogens, such as *Mycobacterium tuberculosis* (Mtb), must acquire iron from their host [5–8]. While iron is abundant in nature, free ferric iron (Fe^3+^) is highly insoluble in aerobic environments and can produce toxic free radicals via the Fenton reaction [9]. To prevent iron toxicity, host iron is typically sequestered within iron binding and storage proteins, such as transferrin and ferritin [10,11], or the small molecule, heme, which is the principal iron reservoir in humans [12]. Like ferric iron, free heme is relatively insoluble and toxic, so it is bound by a variety of hemoproteins. Thus, sequestration of iron/heme from host proteins poses an additional barrier for bacterial pathogens to overcome in host iron acquisition [13]. To overcome these challenges, many Gram-negative and Gram-positive bacterial iron uptake systems employ high affinity iron-chelating siderophores. Most bacteria endogenously synthesize one or more structurally unique siderophores, whereby each siderophore requires a distinct biosynthetic pathway and will employ an associated ferric-siderophore uptake system [14,15]. Notably, some microbes have also evolved mechanisms to use siderophores produced by other organisms present within their micro-environment [16]. Likewise, many bacterial pathogens have developed sophisticated heme uptake systems [13].

Mtb utilizes both iron and heme uptake systems to ensure its survival [17]. Mtb has a relatively well-studied siderophore-dependent iron uptake system, where Mtb siderophores are termed mycobactin (MB) and carboxymycobactin (cMB) [7]. MB and cMB are mixed-type siderophores with both phenolate and hydroxamate moieties. While the two siderophores share an identical core (**S4 and S9 Figs)** [7,18], they differ in hydrophobicity and cellular localization. Owing to its long aliphatic tail, MB is hydrophobic and is thought to be cell-wall and outer membrane associated, though recent studies have also observed MB in the culture filtrate [19,20]. Whereas cMB, which is more hydrophilic than MB due to a shorter aliphatic tail that terminates with a carboxylate group, is secreted and released from the mycobacterial cell to scavenge for host iron [21,22]. The cytosolic biosynthetic pathways of MB and cMB have been well-characterized [17,23]. Once synthesized, the export of MB and cMB across the inner membrane is facilitated by MmpL4/5 along with their accessory periplasmic proteins MmpS4/5 [20], and a small periplasmic protein Rv0455c of unknown function [24]. Iron-loaded or ferric-cMB (Fe-cMB) is imported across the inner membrane by the heterodimeric IrtA/IrtB (IrtAB) membrane transporter [25,26]. However, the proteins involved in shuttling apo-forms of MB and cMB through the Mtb periplasm and cell-wall environment, as well as those that facilitate the transport of iron-loaded MB and cMB from the extracellular space to the inner membrane, are currently unknown. Even less is known about heme uptake in Mtb. Several studies implicate a variety of proteins within the heme uptake pathway by genetic or biochemical methods, which include the secreted protein Rv0203; the cell-surface proteins PPE36, PPE37, PE22, and PE62; a predicted periplasmic binding protein (PBP), FecB2; and the DppABC inner membrane complex dipeptide/heme transporter [27–30]. However, the precise role or mechanism of these proteins in heme acquisition is not fully understood.

Given that Mtb is the causative agent of the disease tuberculosis (TB), and as iron is essential for the growth and survival of Mtb, targeting its iron/heme uptake pathways is a possible strategy to combat TB. For several decades, there have been on-going efforts to interrupt siderophore-mediated iron acquisition through the inhibition of MB and cMB biosynthesis [31,32]. However, the mechanisms of import and export of iron-scavenging siderophores and import of heme represent an alternate set of anti-TB drug targets. Thus, a more in-depth understanding of these import/export mechanisms at an atomic-level resolution is required [33–35].

In Gram-negative and Gram-positive bacteria, both heme and iron uptake pathways utilize PBPs to chaperone and shuttle ferric-siderophores (and sometimes apo-siderophores) and heme within the periplasmic or cell-wall environments to the bacterial inner membrane. Notably, some PBPs are tethered to outer and inner membrane proteins [13–15]. In Mtb, evidence suggests that two predicted ferric-citrate binding PBPs, FecB (Rv3044) and FecB2 (Rv0265c), participate in iron acquisition pathways [17]. Notably, there is no experimental evidence regarding the precise location of Mtb FecB and FecB2, so we will refer to them as substrate binding proteins (SBPs).

FecB is predicted to be part of the IdeR (iron-dependent regulator) regulon, which regulates Mtb iron acquisition [36]. Moreover, a study in *Mycobacterium avium* shows that *fecB* is upregulated under low iron conditions [37], suggesting that Mtb FecB plays a role in iron acquisition. A comprehensive Mtb transposon library study suggests that *fecB* along with a handful of other genes, is required for growth in the presence of Fe-cMB alone [38]; however, this transposon mutant phenotype was not confirmed further. In contrast, another study showed that the MtbΔ*fecB* mutant had no significant growth defect when grown in iron-limited conditions, indicating that FecB is not essential for Mtb iron acquisition [39]. Instead, this study suggested that FecB plays a role in maintaining Mtb cell-wall integrity and is involved in the intrinsic resistance of Mtb to multiple antibiotics [39]. FecB2 has also been implicated in heme uptake. A previous study showed that the MtbΔ*fecB2* mutant displayed a strongly attenuated growth phenotype in the presence of heme alone [28], suggesting that FecB2 is required for Mtb heme-iron acquisition. Ultimately, these studies suggest that FecB and FecB2 may act as Fe-cMB or heme chaperones, but the lack of biochemical and structural data leave their precise roles unclear.

Herein, we have solved the X-ray crystal structures of both Mtb FecB and FecB2 in their apo-forms, which show that the ligand-binding pockets have different potential ligand binding residues, shapes, and electrostatic surface potential, suggesting they bind different ligands. Through structural homology, FecB and FecB2 are predicted to bind ferric-siderophores or heme. We show that both FecB and FecB2 can bind heme, while FecB has a slightly higher affinity for heme than FecB2. Interestingly, FecB binds Fe-cMB whereas FecB2 does not. Mutational analysis of FecB demonstrated that FecB Glu339 is critical for Fe-cMB binding. To pinpoint the primary pathway that Mtb FecB participates in, we utilized both *Mycobacterium smegmatis* (Msm) and Mtb to identify potential interacting proteins of FecB. The identified interaction partners suggest that Mtb FecB plays a role in siderophore-dependent iron acquisition. A similar experiment with FecB2 in Msm shows that FecB2 interacts with FecB in addition to proteins proposed to be involved with heme uptake. These results verify that Mtb FecB2 plays a direct and specific role in heme uptake, whereas Mtb FecB potentially plays a role in both siderophore-dependent and heme-iron acquisition pathways.

## Results

Mtb FecB and FecB2 are quite different proteins with only 24% sequence identity (**S1 Fig**), however both have predicted signal peptides. For *in vitro* biochemical analyses and crystallographic studies, Mtb FecB and FecB2 were cloned in their predicted mature forms without a signal peptide, FecB (without residues 1–28) and FecB2 (without residues 1–38).

### The apo structures of Mtb FecB and FecB2

The structure of the mature form of FecB2 was solved by molecular replacement using the structure of the Msm homolog (MSMEG_0438; PDB code: 4MDY) as the search model. The structure of FecB2 was solved to 2.2 Å resolution in space group P2_1_. The asymmetric unit contains two FecB2 subunits. The FecB2 crystal structure models chain A residues 15–304 and chain B residues 15–303 (where numbering is for the mature FecB2 form and begins at residue 39). The structure of the mature FecB form was solved by molecular replacement using the structure of Mtb FecB2 (PDB code: 4PM4) as the search model. The structure of FecB was solved to 2.0 Å in space group C2, and the asymmetric unit contains two FecB molecules (chain A, 42–356 and chain B, residues 45–357, where numbering is for the unprocessed FecB form). The X-ray crystallography statistics are in **Table 1**.

**Table 1:** Data collection and refinement statistics for *Mycobacterium tuberculosis* H37Rv FecB (Rv3044) and FecB2 (Rv0265c).

	FecB	FecB2
**Data collection**	1 Å	0.9791 Å
Space group	C 2	P 2_1_
Cell dimensions at 100 K		
*a*, *b*, *c* (Å)	138.47, 86.84, 71.58	69.55, 65.43, 72.33
α, β, γ (°)	90, 104.11, 90	90, 116.41, 90
Resolution (Å)^a^	43.33–2.00 (2.07–2.00)	30.00–2.20 (2.28–2.20)
*R*_merge_ ^b^	0.085 (1.039)	0.127 (0.432)
*I* / σ*I*	6.28 (1.3)	10.4 (2.3)
Completeness (%)	97.2 (93.8)	94.5 (98.4)
Redundancy	2.0 (2.0)	2.4 (2.6)
**Refinement**		
Resolution (Å)	43.54–2.00 (2.02–2.00)	29.20–2.19 (2.27–2.19)
No. reflections	54322 (5203)	28034 (2882)
*R*_work_ / *R*_free_^c^	18.9 / 22.2 (34.9 / 38.2)	20.3 / 25.2 (26.6 / 31.3)
No. of residues	612	579
No. atoms		
Protein	4484	4411
Ligand/ion	120	13
Water	414	235
*B*-factors		
Protein	34.4	35.5
Ligand/ion	57.0	48.47
Water	43.2	31.1
R.m.s. deviations		
Bond lengths (Å)	0.004	0.003
Bond angles (°)	0.629	0.787
Ramachandran favored/outliers	97.7 / 0	93.5 / 0.4
PDB ID	7UQ0	4PM4

^a^. Values within parentheses refer to the highest shell.

^b^. *R*_merge_ = ∑∑|I_hkl_—I_hkl_(j) |/∑I_hkl_, where I_hkl_(j) is observed intensity and I_hkl_ is the final average value of intensity.

^c^. R_work_ = ∑||F_obs_|—|F_calc_||/∑|F_obs_| and R_free_ = ∑||F_obs_|—|F_calc_||/∑|F_obs_|, where all reflections belong to a test set of 10% data randomly selected in Phenix.

**Fig 1:**
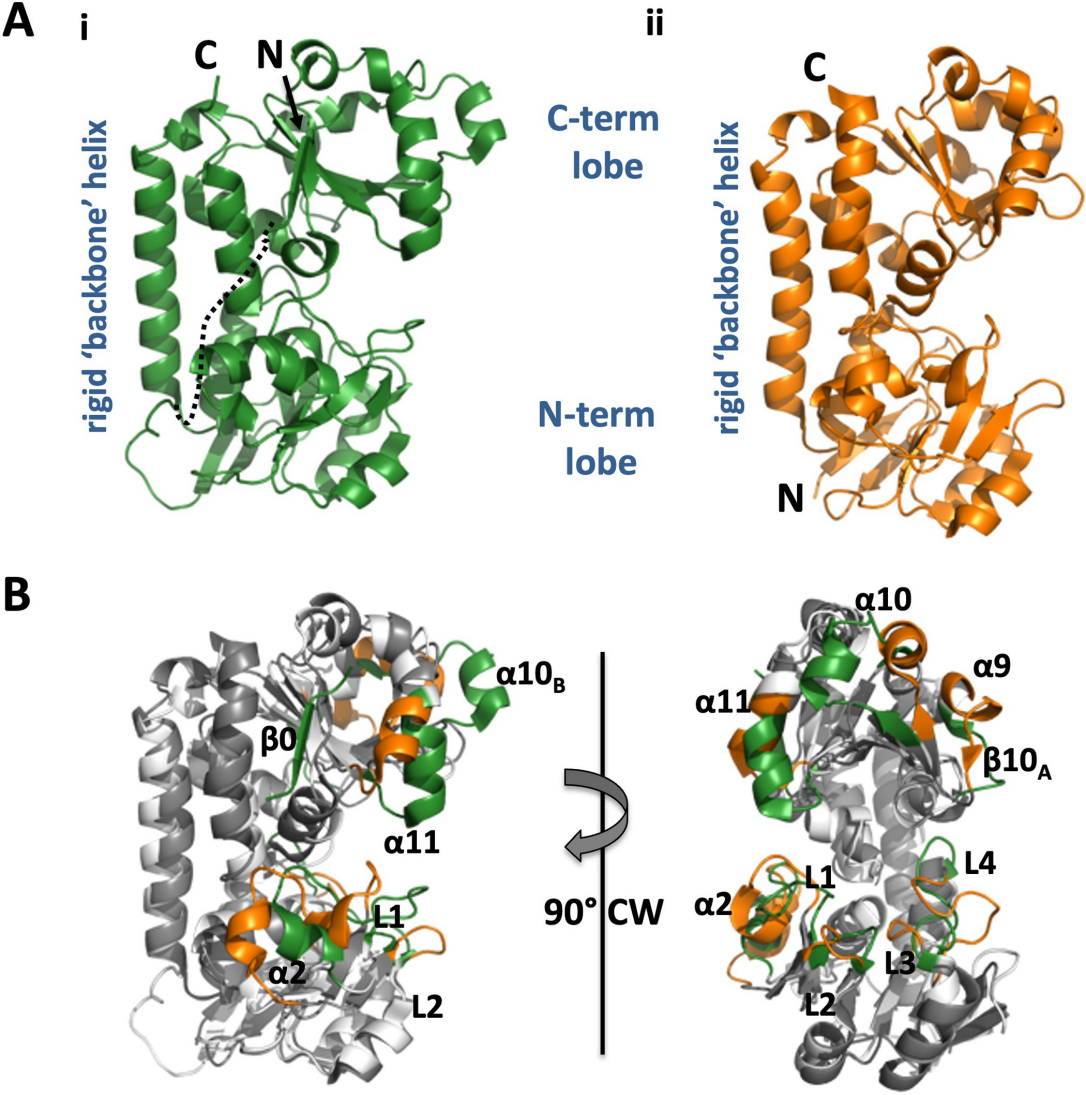
Overview of Mtb FecB and FecB2 structures. **A.** FecB (i, green) and FecB2 (ii, orange) structures are shown in cartoon form. Both are multi-domain proteins with an N-terminal lobe and a C-terminal lobe connected by a rigid ‘backbone’ helix. These features are labelled, as are the N- and C-termini. Notably, FecB has an extra N-terminal sequence that associates with the C-terminal lobe but has an unstructured connection between the N- and C-terminal lobes (dashed black line). **B**. FecB and FecB2 structures are superimposed to highlight the differences in secondary structure. Secondary structure elements that overlay well are colored in white (FecB) and grey (FecB2), while divergent secondary structure is colored as in (**A**) and labelled.

Mtb FecB and FecB2 are both members of the type III PBP family [40]. Each protein structure is composed of N- and C-terminal lobes consisting of mixed α/β structures with a central β-sheet surrounded by α-helices, with the two lobes linked by a rigid “backbone” helix (α6), forming the characteristic fold of the type III PBP family (**Figs 1A and S2A and S2B**). The two lobes of the protein form a cleft, which is the putative ligand-binding pocket. Notably, the N-terminal lobe has a β-hairpin (β1 and β2) followed by a parallel central β-sheet (β5/β4/β3/β6/β7). In FecB, the central β-sheet has a small antiparallel β-strand satellite (β5_B_). The central β-sheet is decorated with four helices (α1, α3, α4 and α5) and two one-turn helices (FecB α1_B_ and α2; FecB2 α2 and α2_B_) near the ligand-binding pocket. In contrast, the C-terminal lobe has a mixed β-sheet (β12, β11, β8, β9, β10). Both have unique additions to this core β-sheet, FecB has an additional β0 strand and FecB2 includes a β10_A_ strand. In both FecB and FecB2, the mixed β-sheet is decorated with four helices (α8, α10, α11, α15) and several short helices (α9, α12, α13, α14, and in FecB, an additional α10_B_).

The Mtb structures of FecB and FecB2 are similar and superimpose with a rmsd of 3.6 Å over the Cα-atoms of 264 residues. Most of the secondary elements overlay well; however, there are some notable differences. In the N-terminal lobe, several loop regions (L1, L2, L3 and L4) and a single-turn helix (α2) near the binding pocket are strikingly different (**Fig 1B**). The FecB and FecB2 C-terminal lobes have differences primarily between α-helices. In FecB there is a single-turn α9 and loop region, and in FecB2 there is an extended α9 followed by an additional β-strand, β10_A_. Additionally, α10 does not align well between the two proteins: in FecB2 there is a single extended α10, but in FecB there are two short helices, α10 and α10_B_, with α10_B_ protruding into the solvent. Finally, in FecB, the N-terminus of α11 diverges from its counterpart in FecB2.

**Fig 2:**
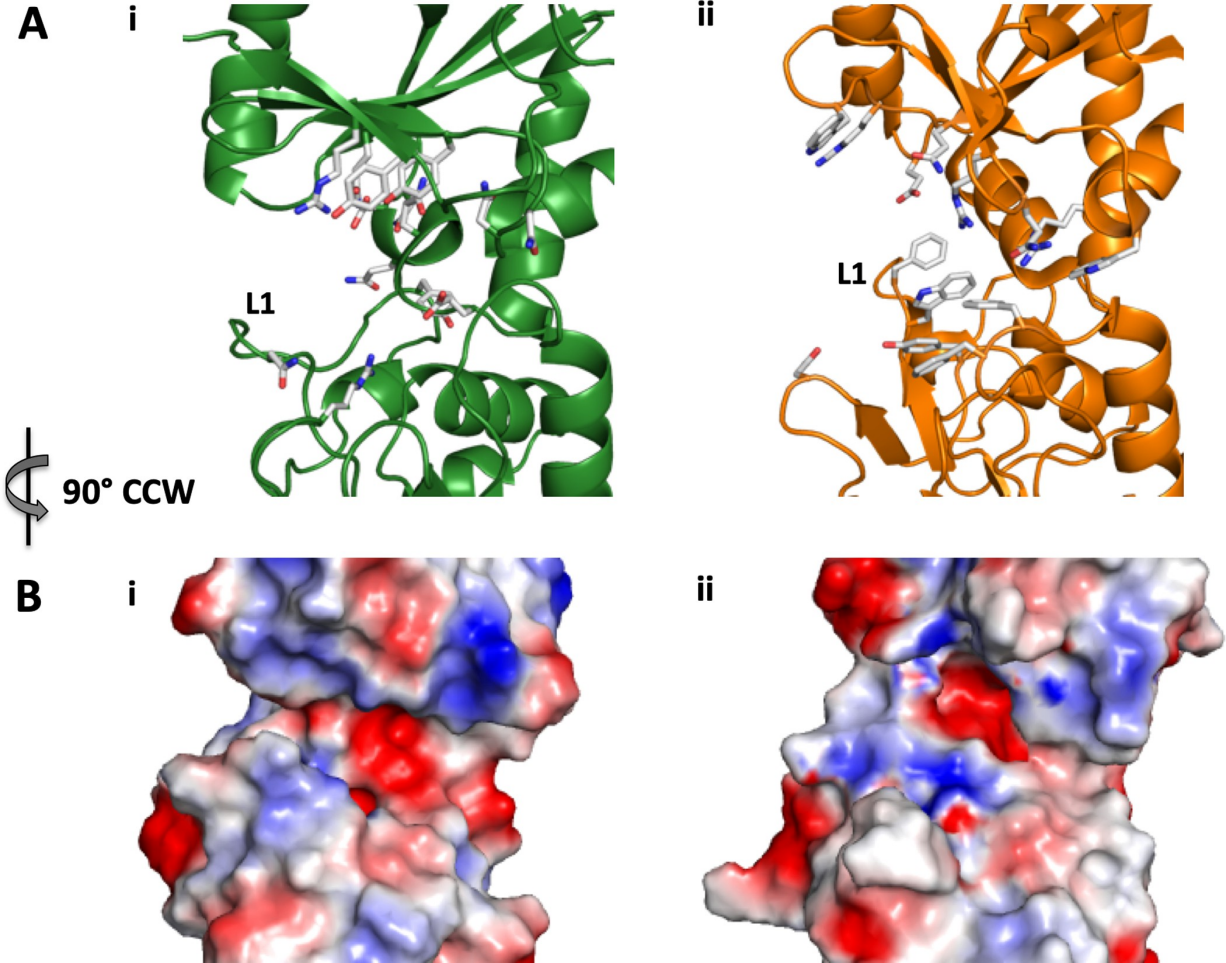
Comparison of the ligand-binding sites for FecB and FecB2. Putative ligand-binding site of FecB (left panels) and FecB2 (right panels) show that the FecB2 ligand binding pocket is in a more open form compared to that of FecB. **A.** FecB and FecB2 are represented in cartoon form and colored as in Fig 1A with potential ligand-binding residues shown as white sticks. **B**. The electrostatic molecular surface for FecB and FecB2 was generated by APBS (Adaptive Poisson-Boltzmann Solver), where white, red and blue represent hydrophobic, negative and positively charged surfaces, respectively.

**Fig 3:**
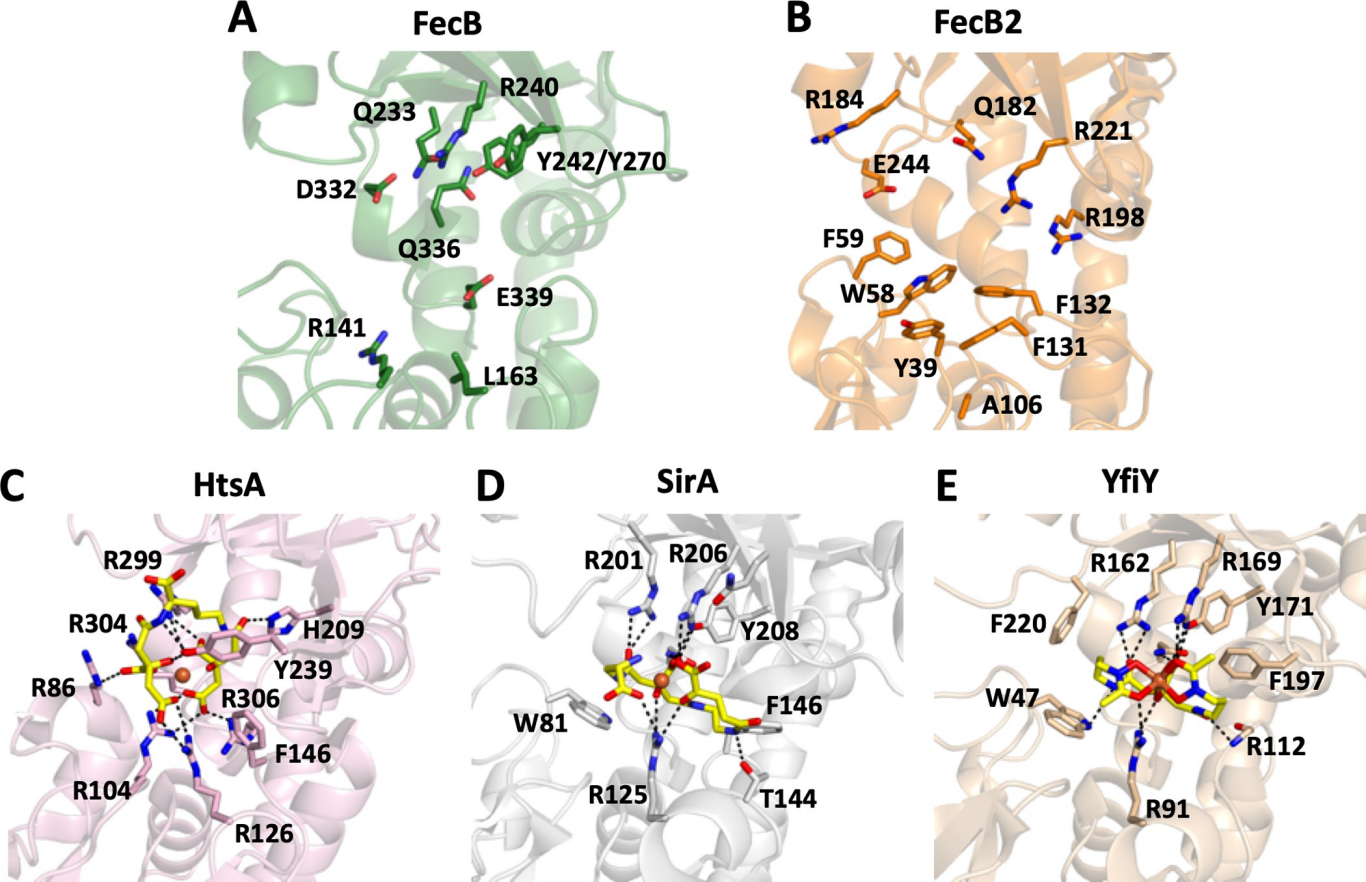
Structural comparison of Mtb FecB and FecB2 with highly similar structural homologs of siderophore-bound PBPs. Ligand-binding sites for **A**. Mtb FecB (green, PDB ID 7UQ0), **B**. Mtb FecB2 (orange, PDB ID 4PM4), **C**. *S*. *aureus* HtsA complexed with ferric-staphyloferrin A (pink, PDB ID 3LI2), **D.** *S*. *aureus* SirA complexed with ferric-staphyloferrin B (white, PDB ID 3MWF), and **E**. *B*. *subtilis* YfiY complexed with ferric-schizokinen (wheat, PDB ID 3TNY) are shown. HtsA, SirA and YfiY are in complex with siderophores (stick representation in yellow with iron as an orange sphere), and coordinating residues are shown as stick representations. Polar interactions are highlighted between the siderophore and coordinating residues (dashed black lines). For FecB and FecB2, conserved or similar residues to HtsA, SirA and YfiY PBPs are shown. Notably, HtsA, SirA and YfiY all have a conserved arginine residue (R126, R125 and R91, respectively) that interacts with the bound siderophore, this arginine is notably absent in FecB and FecB2 with Leu163 and Ala106, respectively, in a similar location.

The substrate binding pocket of FecB is quite different from that of FecB2 in contributing residues, shape, and electrostatic charges (**Figs 2 and S3**). The FecB binding pocket has three tyrosine, four asparagine, three glutamine and two arginine residues along with a glutamate and aspartate residue, **Figs 2A and 3**. In comparison, the substrate binding pocket of FecB2 contains fewer polar or charged residues than that of FecB (**Figs 2 and 3**). The binding cleft of FecB2 contains three arginine and two glutamine residues along with one glutamate, aspartate and tyrosine residue to provide polar contacts with a bound ligand; and three tryptophan and three phenylalanine residues. The resulting FecB pocket is narrower and deeper than the FecB2 ligand-binding pocket, with one side pinched together creating an almost triangular chasm, **Fig 2**. The lip (formed by loop L1) gives the FecB pocket its triangular appearance and the molecular surface is positively charged at the base of the pocket, **Fig 2B**. In contrast, the back of the FecB pocket is negatively charged. Indeed, this charged environment trapped a polyethylene glycol molecule from the crystallographic condition (**S3C Fig**). The FecB2 binding pocket is wider and shallower than the FecB pocket, and the resulting cleft has a predominately neutral base with a positively charged electrostatic surface surrounding the mouth of the negatively charged tunnel. This positively charged patch at the back of the FecB2 pocket juxtaposes the negatively charged patch in FecB at a similar location (**Fig 2B**).

### Comparison of the FecB and FecB2 ligand binding site residues to their structural homologs

Ferric-siderophores and heme are distinct molecules, both designed to coordinate and transport ferric iron. Heme is mainly a large planar porphyrin ring with carbon and carboxylate moieties, while ferric-siderophores have a wide-range of non-planar structures that coordinate a central iron atom typically through carboxylate or hydroxyl groups (**S4A and S4B Fig**). Proteins that recognize heme and ferric-siderophores are finely tuned to coordinate these molecules: polar interactions with charged moieties, hydrophobic contacts with carbon structures, aromatic residues to support aromatic ring structures, and usually in the case of heme, directly coordinate iron (**S4A and S4C Fig**).

In an attempt to assign a ligand for Mtb FecB and FecB2, DALI structural homology searches were performed (**S1 Table)** [41]. Mtb FecB has highest structural similarity to ferric-siderophore binding proteins such as *Bacillus cereus* YfiY (2.5 Å rmsd) [42], *Staphylococcus aureus* SirA (3.1 Å rmsd) [43] and HtsA (3.6 Å rmsd) [44] (**Fig 3**). Like FecB, FecB2 also has highest structural similarity with *B*. *cereus* YfiY (3.0 Å rmsd) and *S*. *aureus* HtsA (3.1 Å rmsd). Overall, however, FecB2 aligns more poorly to its structural homologs except for its close mycobacterial relative Msm FecB2 (0.9 Å rmsd, 65% sequence identity). Notably, all these ferric-siderophore PBPs are from Gram-positive bacteria and none of these structural homologs have high sequence similarity, where YfiY is the closest by sequence to FecB with 23% identity (**S1 Table and S1 Fig**). Mtb FecB and FecB2 also have high structural homology to other ferric-siderophore and heme PBPs. Indeed, both proteins have structural homology to PBP heme binding proteins in *S*. *aureus* (IsdE) [45], *Shigella dysenteriae* (ShuT) [46] and *Vibrio cholerae* (HutB) [47]. The structural homology of Mtb FecB and FecB2 to PBPs suggest that their ligands are potentially ferric-siderophores or heme (**S4A and S4B Fig**).

Both FecB and FecB2 have high structural similarity with *S*. *aureus* HtsA (**Fig 3**) [44], a PBP membrane-tethered component of the ATP-binding cassette-type (ABC) transporters, and binds ferric-staphyloferrin A for import into the cell (**S4 Fig)** [48]. Within the HtsA-ferric-staphyloferrin A complex, iron is not directly coordinated by HtsA and utilizes a combination of tyrosine, histidine, phenylalanine and four arginine residues to bind the siderophore noncovalently (**Figs 3 and S4C**) [44]. Of these HtsA residues, two critical siderophore binding residues Arg104 and His209, align to FecB Arg141 and Tyr242, respectively. While for FecB2, only one conserved HtsA siderophore coordinating residue, Phe146, aligns to FecB2 Phe131 or Phe132 (**Figs 3 and S1)**.

*S*. *aureus* SirA is another membrane-associated PBP, which specifically recognizes staphyloferrin B (**S4A and S4B Fig)** [43]. Within the SirA-ferric-staphyloferrin B complex, SirA utilizes a combination of tyrosine, threonine, asparagine, arginine, tryptophan and phenylalanine residues to bind the siderophore noncovalently (**Figs 3 and S4C4**). There is some conservation of residues in the SirA siderophore binding site to those in FecB and FecB2. In FecB, residues Arg240 and Tyr242 align with SirA Arg206 and Tyr208. In FecB2, SirA Trp81 aligns to FecB2 Trp58 or Phe59, and Arg201 and Phe146 aligns to FecB2 Arg184, and Phe131 or Phe132 (**Figs 3 and S1)**.

*B*. *subtilis* YfiY is a PBP that binds iron-hydroxamate siderophores and delivers them to other members of the ABC transporter complex [42]. YfiY binds schizokinen (PDB code: 3TNY) via three arginine residues, an asparagine and a tyrosine along with two aromatic residues (**Figs 3 and S1 and S4)**. In FecB, YfiY residues Arg169, Tyr171, and Asn261 are conserved (FecB Arg240, Tyr242, Gln336). In FecB2, only one residue YfiY Trp47 is conserved (FecB2 Trp58 or Phe59).

Notably, within the N-terminal lobe of HtsA, SirA and YfiY, each protein binds its respective siderophore by a superimposable arginine residue (**Fig 3**). In contrast, FecB and FecB2 do not have an arginine residue at this position but a hydrophobic, uncharged residue, FecB Leu163 and FecB2 Ala106 (or Gly107).

As stated above, the structural similarity of FecB and FecB2 to bacterial heme-binding PBPs was considerably lower than for siderophore-binding PBPs (**S1 Table**). IsdE coordinates heme-iron by a proximal methionine and distal histidine [45] (**S4C Fig)**, and neither FecB nor FecB2 have a methionine or histidine residue at these locations in their ligand binding pockets. In contrast, HutB, PhuT and ShuT [46,47] coordinate heme-iron via a proximal tyrosine residue. FecB2 has an N-terminal lobe tyrosine (Tyr39) that could potentially coordinate to heme-iron whereas FecB has no tyrosine residues in its N-terminal lobe, but it does have two in its C-terminal lobe (Tyr242 and Tyr270) that may be involved in binding heme.

As discussed above, both FecB and FecB2 binding pockets are rich in aromatic tyrosine, phenylalanine and/or tryptophan residues that can coordinate large nonpolar regions of bound siderophores or heme. Both FecB and FecB2 also possess arginine residues that could coordinate charged elements in the bound ligand, however FecB has a more charged binding pocket surface. Conservation of FecB residues Arg141, Arg240, Tyr242, and Gln336, and FecB2 residues Trp58, Phe59, Phe130, Phe132, and Arg184 with structural homologs suggest they could be potential siderophore interacting residues.

### Determination of potential substrates of FecB and FecB2

**Fig 4:**
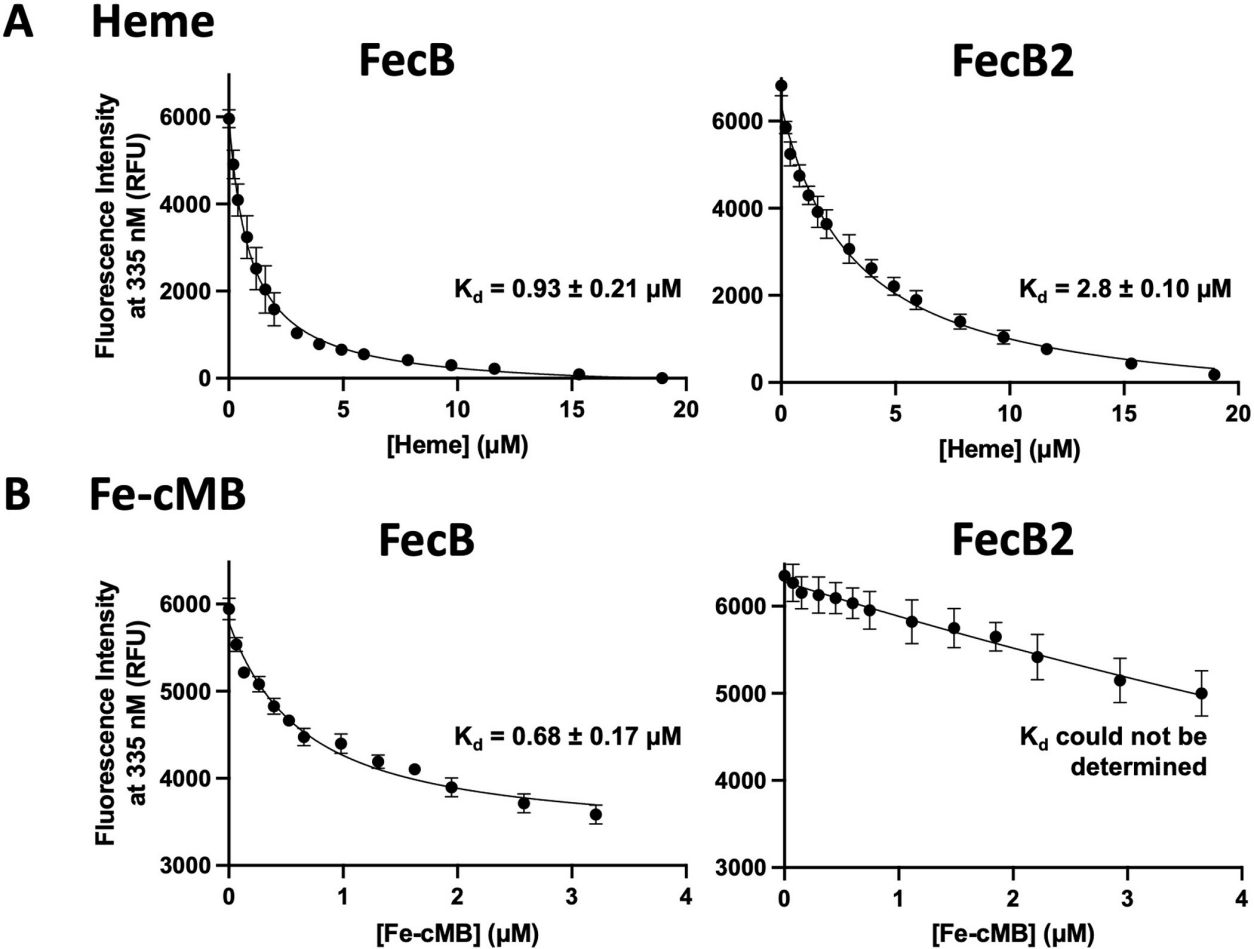
Both FecB and FecB2 bind heme, while FecB preferentially binds Fe-cMB compared to FecB2. Representative fluorescent emission intensities at 335 nm after excitation at 280 nm of 100 nM FecB (left panels) and 100 nM FecB2 (right panels) with increasing concentrations of **A.** heme and **B.** Fe-cMB. Notably the experiments in (**A**) were performed with tagless proteins. Curves were fit using Eq 1 and affinities (K_d_) are included for each titration. In (**A**) FecB binds heme with a higher affinity than FecB2, in contrast to (**B**) showing that FecB binds Fe-cMB whereas FecB2 does not.

We used a previously described fluorescence-based assay to determine the affinities of heme and Fe-cMB (and other potential ligands) to FecB and FecB2 [49]. Both FecB and FecB2 constructs with hexa-histidine (His) tags have high nanomolar to micromolar affinities to heme, 1.20 ± 0.10 μM and 0.87 ± 0.06 μM, respectively (**S5 Fig and Table 2)**. Previous studies have shown that His tags can interfere with heme binding studies [50–52], thus we determined the affinity of heme to FecB and FecB2 in the absence of a His tag (**Figs 4 and S5)**. For FecB, the affinity for heme was similar in the presence or absence of the His tag. However, in the case of FecB2, the affinity for heme was reduced 3-fold in the absence of the His tag (**Table 2**). These results indicate that both FecB (0.93 ± 0.21 μM) and FecB2 (2.8 ± 0.1 μM) bind heme.

**Table 2:** FecB and FecB2 K_d_’s for heme or Fe-cMB.

Protein	Ligand	K_d_ (μM)	Fold-decrease
FecB2-His FecB2	Heme	0.87 ± 0.06 2.8 ± 0.10	N/A
FecB-His FecB FecB-His Refolded	Heme	1.20 ± 0.10 0.93 ± 0.21 0.88 ± 0.26	N/A
FecB2-His	Fe-cMB	n.d.	‐‐
FecB-His FecB FecB-His Refolded	Fe-cMB	0.63 ± 0.12 0.68 ± 0.17 0.55 ± 0.12	N/A
**FecB-His variants**			
R141S L163R Q233S R240S Y242S Y270S E272S D332S Q336S E339S R240S/E339S Y242S/E339S	Fe-cMB	1.13 ± 0.37 1.16 ± 0.11 1.05 ± 0.34 1.10 ± 0.32 0.88 ± 0.19 0.94 ± 0.24 1.73 ± 0.36 0.99 ± 0.21 1.40 ± 0.55 n.d n.d n.d.	1.7 1.5 1.5 1.6 1.3 1.4 2.5 1.5 2.1 >10 ‐‐ ‐‐
**FecB2 variants**			
Y39S W58S R184S	heme	4.1 ± 0.1 4.0 ± 0.3 3.4 ± 0.2	1.5 1.4 1.2

When the affinity of Fe-cMB to FecB and FecB2 was tested, we observed a dramatic difference in their preference for Fe-cMB. FecB has high nanomolar affinity to Fe-cMB (0.68 ± 0.12 μM), whereas the affinity of FecB2 to Fe-cMB was too weak to be determined due to the low solubility of Fe-cMB (**S6 Fig and Table 2**). Moreover, the affinity of Fe-cMB for FecB was the same in the presence or absence of the His tag (**Figs 4 and S6 and Table 2**). These results suggest that while both FecB and FecB2 can bind heme, only FecB binds Fe-cMB with high affinity whereas FecB2 does not show measurable binding.

**Fig 5:**
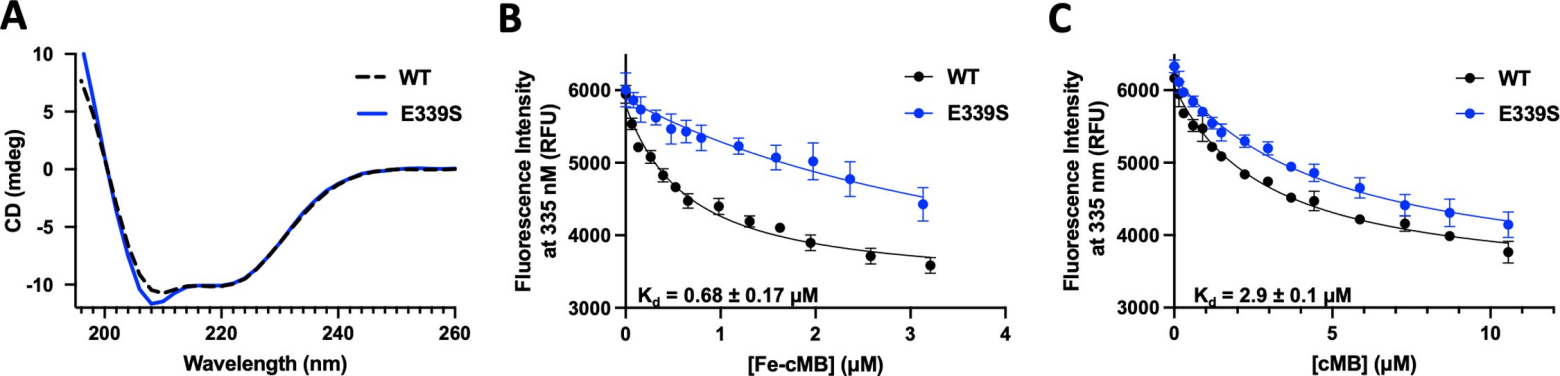
The affinity of Fe-cMB is attenuated for the FecB-E339S variant compared to WT-FecB. **A**. CD shows that both WT-FecB (dashed black line) and FecB-E339S (solid blue line) have the same secondary structural elements. **B & C**. Comparison of representative fluorescent emission intensities at 335 nm after excitation at 280 nm with increasing concentrations of (**B**) Fe-cMB and (**C**) apo-cMB binding to WT-FecB (100 nM, black line) and FecB-E339S (100 nM, blue line). Curves were fit using Eq 1 and cMB affinities (K_d_) are included for each titration for WT FecB.

As FecB binds Fe-cMB, and some ferric siderophore PBP-like proteins also bind to their apo-form [53,54], we wanted to test if FecB also binds to apo-cMB. We showed that the affinity of FecB to apo-cMb (2.9 ± 0.1 μM) was ∼4-fold lower when compared to Fe-cMB (**Figs 5C and S9A**), but still has a low micromolar affinity. Next, we asked the question if FecB could also bind a hydrophobic form of Fe-cMB, ferric-mycobactin J (Fe-MBJ) from *Mycobacterium paratuberculosis*. MBJ has a similar structure to Mtb mycobactin (**S9B Fig**). When comparing the affinities to FecB, we observed a ∼20-fold decrease of affinity for Fe-MBJ (K_d_ ∼11 μM) compared to Fe-cMB. These results suggest that FecB has a strong preference for Fe-cMB over Fe-MBJ, and that it also binds to apo-cMB.

Thus far, we have demonstrated that FecB binds Fe-cMB with high nanomolar affinity and its apo form with low micromolar affinity. We have also shown that FecB preferentially binds Fe-cMB over the more hydrophobic mycobacterium siderophore, Fe-MBJ. With this in mind, we also wanted to establish if FecB could bind other bacterial ferric-siderophores. We tested *E*. *coli* ferric-enterobactin and *P*. *aeruginosa* ferric-pyoverdines (Fe-Pyo), as *P*. *aeruginosa* also resides in the lung environment. Both ferric siderophores showed at least 40-fold weaker affinity to FecB compared to Fe-cMB (**S9C Fig**). This indicates that while FecB can accommodate and bind both heme and cMB in its apo or ferric form, it is not able to accommodate Fe-MBJ or other bacterial ferric siderophores tested.

In summary, these results firmly place FecB2 in the heme uptake pathway, which is further strengthened by a previous study that showed severe growth attenuation of the MtbΔ*fecB2* mutant in the presence of heme as the sole iron source [28]. FecB also binds heme but has a higher affinity for Fe-cMB with the additional caveat that it can also bind apo-cMB, suggesting that it may play roles both in heme acquisition and cMB-mediated iron uptake.

### Determination of FecB residues that coordinate to Fe-cMB

We have observed that FecB binds Fe-cMB with high nanomolar affinity while the affinity of Fe-cMB for FecB2 was too weak to be determined. As FecB is the first SBP shown to bind Fe-cMB *in vitro*, we interrogated FecB residues that could be involved in Fe-cMB binding. As described above, several potential Fe-cMB-interacting residues were identified in FecB due to structural conservation (**Figs 3 and S1**). To maintain protein solubility, these surface-exposed potential ligand-coordinating residues (Arg141, Gln233, Arg240, Tyr242, Tyr270, Glu272, Asp322, Gln336, and Glu339) were mutated to serine with one exception: Leu163 was mutated to an arginine as it superimposed onto a conserved arginine residue in the structures of HtsA, SirA and YfiY (Arg126, Arg125 and Arg91, respectively), **Fig 3**. Notably, all resulting FecB variants were insoluble, and were purified under denaturing conditions and refolded. To ensure that the FecB variants were correctly folded, circular dichroism (CD) spectroscopy was utilized. Wild-type (WT) FecB-His and its variants had similar CD traces (**Figs 5A and S7 and S2 Table**) suggesting that the FecB variants were correctly folded. We also tested whether refolded WT FecB had similar affinity for heme and Fe-cMB to that of native WT FecB. We observed that the affinity to Fe-cMB between the refolded and the natively purified FecB proteins were similar (**S8 Fig and Table 2**).

For all ten FecB variants, we determined their affinity to Fe-cMB (**S10 Fig and Table 2**). Out of the ten variants tested (R141S, L163R, Q233S, R240S, Y242S, Y270S, E272S, D322S, Q336S, and E339S), there was only one variant, E339S, (**Fig 5B**) that had a considerably reduced affinity to Fe-cMB. Ultimately, the affinity of the E339S variant was so diminished that it was unmeasurable due to the solubility limits of Fe-cMB. This acidic residue, Glu339, is situated at the back of the FecB ligand binding pocket (**Fig 3A**). A previous structure-guided study that probed the effect of known siderophore interacting PBP residues upon ferric-siderophore affinity demonstrated that out of the fifteen HtsA ferric-staphyloferrin A interacting residues tested, only three residues contribute significantly to the affinity [55]. Therefore, our observation that out of ten FecB residues predicted to interact with Fe-cMB tested, only one mutated residue, E339, had a strong effect on its binding affinity to Fe-cMB is not unprecedented, and does not rule out that the other FecB predicted interacting residues could be involved in binding Fe-cMB.

To further establish that Fe-cMB is a ligand for FecB, we constructed two double mutants using the FecB-E339S variant as the background with the additional mutation of R240S or Y242S. Notably, the two single FecB-R240S and FecB-Y242S variants only showed a modest decrease in affinity for Fe-cMB. The resulting double mutants, FecB-R240S/E339S and FecB-R242S/E339S, showed an even further diminished affinity towards Fe-cMB compared to the single FecB E339S mutant (**S10 Fig and Table 2**), suggesting that Fe-cMB is a specific ligand to FecB.

As FecB also binds apo-cMB and heme, we tested the affinity for both small molecules to the FecB E339S mutant. It was observed that FecB E339S had a similar affinity to heme compared to WT FecB (K_d_ is 0.78 ± 0.3 μM) and the affinity of apo-cMB only decreased in affinity by ∼1.7 fold (K_d_ is 4.8 ± 0.3 μM) (**Figs 5C and S9C**), further supporting that FecB Glu339 plays an important role in specifically binding Fe-cMB.

### Probing FecB2 residues that coordinate to heme

In an attempt to pinpoint FecB2 residues involved in heme binding, several residues were identified due to conservation with heme interacting residues from bacterial PBP homologs. The potential FecB2 heme interaction residues mutated to serine were Tyr39, Trp58, and Arg184; we propose that Tyr39 coordinates heme-iron. Surprisingly, we observed no significant decrease in affinity of these FecB2 variants compared to WT FecB2 (**S11 Fig**). Further biophysical characterization of the FecB2-heme complex and its variants is warranted.

### Investigation into the protein interaction partners of FecB

FecB appears to bind both Fe-cMB and heme. To better understand the role that FecB plays in mycobacterial iron acquisition, we sought to identify FecB interacting protein partners *in vivo*. To achieve this, we utilized co-immunoprecipitation (co-IP) followed by protein identification using tandem mass spectrometry (MS/MS) in the non-pathogenic Mtb model organism, Msm. The closest Msm homolog to Mtb FecB, Msmeg_2319 was cloned to encode a C-terminal FLAG tag [56]. With the intention of upregulating the iron acquisition machinery, Msm cultures were first grown under iron deplete conditions before supplementing with low iron (20 μM ferric ammonium citrate), and a control consisting of Msm bearing a FLAG expressing vector was run in tandem. First, we wanted to check that the signal peptide of Msm-FecB was processed, implying that it is exported to the periplasmic and cell-wall environments. Indeed, co-IP of the Msm-FecB-FLAG lysate with anti-FLAG beads followed by MS/MS resulted in all peptides assigned to Msm-FecB matching its full-length sequence apart from the first N-terminal 42 residues (**S12A Fig**), suggesting that the signal peptide was cleaved. Additionally, this allowed us to limit our protein partner interaction search to predicted periplasmic proteins, membrane and cell-wall proteins and their complexes. The resulting analysis of co-IP peptides revealed potential Msm-FecB-FLAG interacting protein partners that were not observed in the control co-IP (**S1 Data**).

Several identified interacting partners of FecB suggest that it is involved in siderophore-dependent iron acquisition (**Table 3A**). One of the interacting proteins of FecB is IrtB, which together with IrtA forms the heterodimeric inner membrane complex (IrtAB) that imports Fe-cMB into the mycobacterial cytosol [25,26]. Another interactor is MmpL5, where MmpL5 is an inner membrane protein involved in the export of Mtb siderophores [20]. We also observed MmpS5 and MmpS4 as FecB interacting candidates, which are known accessory proteins of MmpL5 and its family member MmpL4, respectively [20]. Finally, we identified one of the subunits involved in the ESX-3 complex, EccE3, where ESX-3 is a Type VII secretion system known to be involved in iron and heme homeostasis [19,57,58]. Several interacting partners are involved in the upregulation of iron sequestration machinery, indicating that FecB is involved in Mtb apo-siderophore export, ferric-siderophore import, or both.

**Table 3:** Top periplasmic and membrane protein hits of interest from Msm co-IPs with (**A**) FecB-FLAG, (**B**) MmpS4-FLAG, (**C**) MmpS5-FLAG and (**D**) FecB2-FLAG.

**A**				
**Protein**	**Msm *#***	**Mtb #**	**Coverage**	**Spectral Counts**
**FecB**	**MSMEG_1039/2319**	**Rv3044**	**87.1%**	**953**
MmpS4	MSMEG_0380	Rv0451c	41%	11
MmpS5	MSMEG_0226	Rv0677c	26%	4
IrtB	MSMEG_6553	Rv1349	6.9%	4
MmpL5	MSMEG_0225	Rv0676c	2.7%	2
EccE3	MSMEG_0626	Rv0292	8.7%	5
**B**				
**Protein**	**Msm #**	**Mtb #**	**Coverage**	**Spectral Counts**
**MmpS4**	**MSMEG_0380**	**Rv0451c**	**97.1%**	**294**
FecB	MSMEG_1039/2319	Rv3044	15.4%	5
**C**				
**Protein**	**Msm #**	**Mtb #**	**Coverage**	**Spectral Counts**
**MmpS5**	**MSMEG_0226**	**Rv0676c**	**80.3%**	**54**
FecB	MSMEG_1039/2319	Rv3044	25.2%	12
**D**				
**Protein**	**Msm #**	**Mtb #**	**Coverage**	**Spectral Counts**
**FecB2**	**MSMEG_0438**	**Rv0265c**	**95%**	**590**
FecB	MSMEG_1039/2319	Rv3044	54.9%	68
OppA	MSMEG_0643	Rv1280c	12.6%	10
OppD-2	MSMEG_0639	Rv1281c - 2^nd^ half	17.3%	6
OppD-1	MSMEG_0640	Rv1281c – 1^st^ half	6.7%	2

To corroborate our co-IP results for FecB, we performed reverse co-IPs with the small periplasmic proteins, MmpS4 and MmpS5. As Msm has several homologs of Mtb MmpS4 and MmpS5, we used the closest Msm homolog to Mtb MmpS4 and MmpS5 as the bait proteins: MSMEG_0380 (Msm-MmpS4) and MSMEG_0226 (Msm-MmpS5), respectively. As with Msm-FecB, both Msm-MmpS4 and Msm-MmpS5 were engineered to encode C-terminal FLAG tags. Msm cells containing the Msm-MmpS4-FLAG or Msm-MmpS5-FLAG vectors were grown under similar conditions to the Msm-FecB-FLAG experiment, followed by co-IPs and protein identification by MS/MS (**S2 and S3 Datas).** For both Msm-MmpS4-FLAG and Msm-MmpS5-FLAG lysates, the reverse co-IP results indicated that Msm-FecB is an interacting protein partner (**Table 3B and 3C**). These results confirm that FecB does indeed interact with MmpS4 and MmpS5 in Msm *in vivo* and lends credence to the original co-IP interacting partners described above.

### FecB interacts with MmpS5 in Mtb

**Fig 6:**
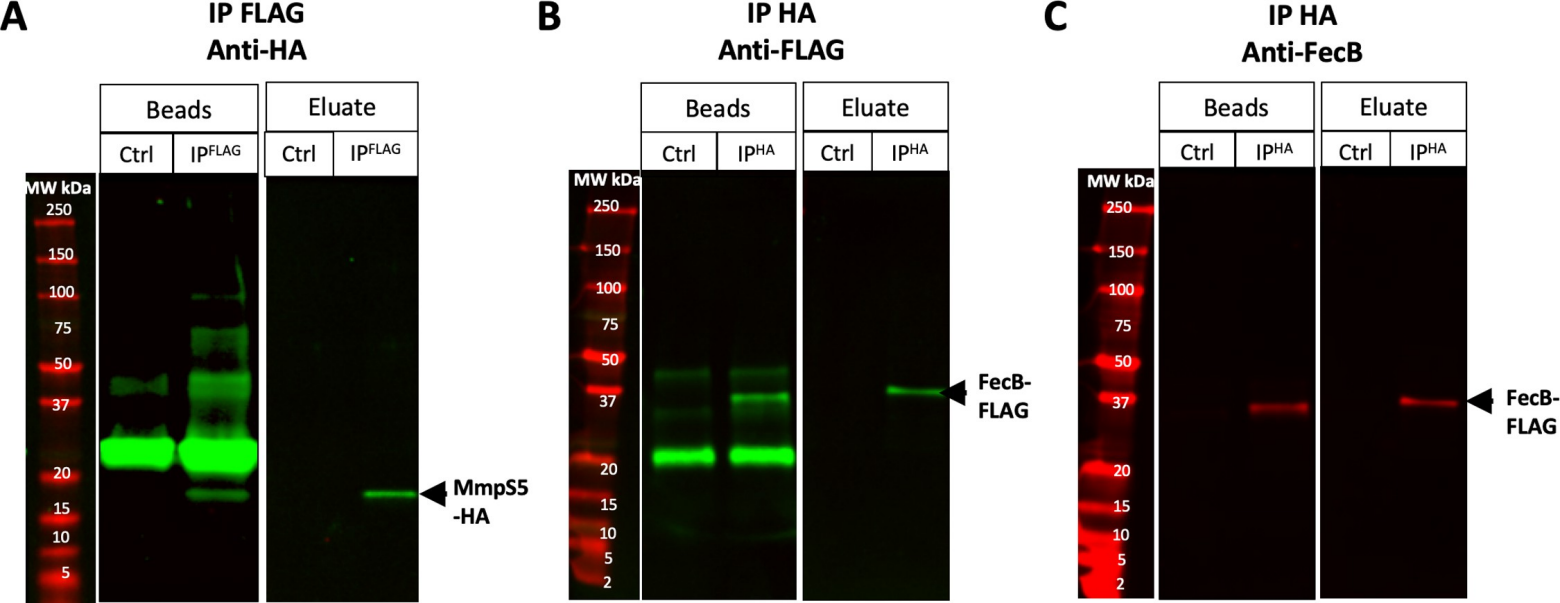
Western Blot analysis of Mtb co-IPs suggest that FecB interacts with MmpS5. Western Blot analysis of protein co-IPs with MtbΔ*fecB* with vectors expressing FecB-FLAG and MmpS5-HA, and the negative control whereby Mtb contains vectors that express the tags alone. Western Blot analysis of both the boiled beads and eluate of the (**A**) co-IP using the anti-FLAG beads to pull down FecB-FLAG, probed with an anti-HA antibody that recognizes MmpS5-HA, (**B**) co-IP using the anti-HA beads to pull down MmpS5-HA, probed with an anti-FLAG antibody that recognizes FecB-FLAG, and (**C**) co-IP using the anti-HA beads to pull down MmpS5-HA, probed with an anti-FecB antiserum that recognizes FecB.

To determine if FecB interacts with MmpS5 in a pathogenic mycobacterium, we tested this protein-protein interaction in Mtb utilizing co-IP experiments followed by western blot. Using the MtbΔ*fecB* mutant [39], we introduced two vectors: the first encoded a C-terminal FLAG tagged FecB and the second encoded a C-terminal hemagglutinin (HA) tagged MmpS5. We used WT Mtb with vectors only expressing the tags alone as the control. Cells were grown in regular 7H9 media and co-IPs were performed from cell lysates with anti-FLAG beads and anti-HA beads. Western blots were carried out and were blotted with either anti-FLAG or anti-HA primary antibodies, or anti-FecB antiserum (**S13 Fig**). The western blot of the co-IP using FecB-FLAG as the bait protein and analyzed with anti-HA primary antibody showed a band corresponding to MmpS5 from the eluted anti-FLAG beads (**Fig 6A**), indicating FecB pulled down MmpS5. The reverse experiment, in which MmpS5-HA was used as the bait protein, and probed with anti-FLAG primary antibody, showed an eluted band corresponding to FecB (**Fig 6B**), indicating MmpS5 pulled down FecB. Finally, when the western blot of the eluted anti-HA beads that pulled down MmpS5-HA (**S13 Fig**) was probed with anti-FecB antiserum, a clear band corresponding to FecB was observed (**Fig 6C**), reinforcing the results that MmpS5 binds to FecB. These results confirmed the above observation in the Msm FecB co-IP experiments and suggest that Mtb FecB interacts with Mtb MmpS5 *in vivo*.

### Investigation into the protein interaction partners of FecB2

To identify potential protein interaction partners for FecB2, we carried out a similar co-IP experiment as described for FecB. The closest Msm homolog to Mtb FecB2, Msmeg_0438, was cloned to encode a C-terminal FLAG tag and grown under normal iron conditions. We then performed a co-IP on the cell lysate with anti-FLAG beads followed by protein identification by MS/MS. First, we determined if the predicted signal peptide had been cleaved, and in contrast to FecB, we observed peptides for 95% of the FecB2 sequence suggesting that the signal peptide is unprocessed (**S12B Fig**). It was previously shown that Mtb FecB2 resides in the membrane fraction but is not cell-surface exposed [28], and the observation that the signal peptide is intact suggests that FecB2 is tethered to the periplasmic side of the inner membrane by a single transmembrane helical pass. When identifying potential protein partners, as for FecB, we limited our search to predicted membrane, periplasmic and cell-wall proteins, and to proteins not observed in the control co-IP (**S4 Data**).

A couple of identified interacting partners of FecB2 corroborate that it is involved in heme-iron acquisition (**Table 3D**). Notably, one of the top interacting proteins was FecB, which we have demonstrated to bind heme *in vitro*. Additionally, we have identified two proteins, OppA and OppD, involved in the ATP-dependent oligopeptide transport system. Thus, the Opp transport system in mycobacteria may be involved in heme import.

## Discussion

The *in vitro* FecB and FecB2 ligand binding experiments with Fe-cMB and heme indicate that only FecB binds Fe-cMB, whereas both FecB and FecB2 bind heme. We have also shown that FecB binds apo-cMB at low micromolar affinity. These results place both FecB and FecB2 in the heme-iron acquisition pathway. The results also strongly suggest that FecB plays a role in cMB-dependent iron acquisition.

FecB and FecB2 both have the Type III PBP fold and have high degrees of structural similarity. Nonetheless, there are major differences within the ligand binding pockets as highlighted in the results section. Additionally, the volume of the ligand binding pocket of FecB is substantially larger than that of FecB2 (526 Å^3^ vs 156 Å^3^, as determined by pCast [59]). As FecB binds both heme and cMB, the increased size of its binding pocket could reflect this promiscuity or could explain why FecB is able to bind Fe-cMB—a larger, bulkier ligand than heme (**S4 Fig**)—while FecB2 cannot.

The affinities of Mtb FecB to heme and cMB, and Mtb FecB2 to heme are on a similar affinity scale to other homologous PBPs for their specific ligand and supports FecB and FecB2 being located in the cell-wall or periplasm. HtsA and SirA, highly specific outer membrane receptors, bind ligands with low nanomolar affinities, while periplasmic proteins, FhuD1 and FhuD2, have ligand affinities in the mid-nanomolar to low micromolar range. Also, *E*. *coli* FhuD has been shown to interact with TonB, suggesting that it resides in the periplasm in proximity to the outer membrane; it has a ligand affinity in the high nanomolar to low micromolar range. As the affinities of Mtb FecB to cMB or heme and Mtb FecB2 to heme are within the high nanomolar to low micromolar range, these ligand affinities are consistent to other PBPs located in the periplasmic and cell-wall environments. As Mtb FecB2 does not bind Fe-cMB, this study suggests that heme is its specific ligand. Furthermore, we tested the affinity of three other ferric-siderophores for FecB, which have affinities in the mid micromolar range and above, suggesting that cMB and heme are specific ligands for Mtb FecB.

The FecB protein-protein interaction experiments described herein place FecB in the cMB-mediated iron acquisition pathway. Further, as FecB binds Fe-cMB *in vitro*, there is strong evidence for the involvement of FecB in Fe-cMB import. It should be noted, in a previous study, under iron-limiting and iron-replete conditions the MtbΔ*fecB* mutant did not display major growth defects relative to the WT Mtb, suggesting that it is not essential for iron acquisition [39]. Our studies suggest that FecB plays an important role in cMB utilization, however there may be an alternate iron acquisition pathway that is cMB-independent and FecB-independent or functional redundancy in a FecB-like protein. Interestingly, *E*. *coli* FecB is part of the larger Fec system that sequesters ferric citrate from the host and shuttles it to the cytosol [60]. Mtb, apart from FecB and FecB2, does not encode any homologs of other Fec system proteins. Instead, in the Msm FecB co-IP experiment, we observe that IrtB is an interaction partner of FecB, where IrtAB is the inner membrane transporter that imports Fe-cMB into the cytosol [25,26]. We propose that Fe-cMB loaded FecB docks to the periplasmic side of IrtAB and induces a conformational change in IrtAB to facilitate ATP-dependent import of Fe-cMB to the cytosol.

FecB is not only implicated in Fe-cMB import; this study also supports the involvement of FecB in cMB export. In vitro, FecB has a low micromolar affinity for apo-cMB. Additionally, we observe MmpS4 and MmpS5 as direct or indirect FecB interacting partners. MmpS4 and MmpS5 are essential periplasmic accessory proteins of the inner membrane cMB/MB exporters MmpL4 and MmpL5 [20], suggesting that FecB may also play a role in cMB efflux. Once MmpL4/5 proteins transport siderophores into the periplasm, little is known about the final translocation or incorporation of these molecules into the cell-wall and outer membrane architectures. While some Gram-negative bacteria couple inner membrane efflux pumps with TolC channels that span the outer membrane to secrete siderophores into the extracellular environment [61,62], Mtb does not possess a TolC homolog, so this avenue is not available. However, a small helical periplasmic protein, Rv0455c, was recently shown to be important in the secretion of Mtb siderophores into the extracellular space [24]. Notably, Rv0455c was not a protein interaction hit in our FecB co-IP experiment. We propose, as FecB binds to apo-cMB and directly or indirectly interacts with MmpS4/5, that FecB could chaperone Mtb apo-cMB to the outer membrane.

As discussed above, our results indicate that FecB could be involved in both the export of cMB and the import of Fe-cMB. Although most PBPs are thought to bind only ferric-siderophores, several PBPs bind and utilize both their iron-loaded and iron-free siderophores [53,54]. Notably, *P*. *aeruginosa* utilizes a complex of two PBPs, FpvF and FpvC, that forms when bound to the *Pseudomonas* ferric-siderophore, Fe^3+^-pyoverdine, and shuttles it through the periplasm to the inner membrane, where ferric iron is reduced for import into the cytosol. FpvF then shuttles the apo-siderophore to the siderophore export system [54,63]. *E*. *coli* FecB binds both ferric-citrate and citrate, although ferric-citrate is bound with higher affinity than citrate alone [53], which is a similar affinity differential observed for Mtb FecB between the apo and ferric cMB forms.

As both FecB and FecB2 bind heme, and as one of the top protein interaction hits of the FecB2 co-IP experiments is FecB, it is possible that both proteins are required for trafficking heme through the cell-wall and periplasmic envirnoments to the inner membrane. As mentioned above, *P*. *aeruginosa* has two PBPs, FpvF and FpvC, that work in concert to shuttle its ferric-siderophore through the periplasm to the inner membrane [64,65]. Mtb may require both FecB and FecB2 to transport heme to the inner membrane. Our observation that the signal peptide of FecB is processed whereas the signal peptide of FecB2 is not, suggests that FecB2 may be tethered to the inner membrane. With this in mind, we propose that FecB would first transfer heme to FecB2, and in turn, heme would be transferred to the inner membrane complex formed by the other two FecB2 interacting partners, OppA and OppD. A previous study established that the Mtb DppA, part of the DppABC inner membrane complex dipeptide/heme transporter, is required for Mtb growth in heme alone [28]. The closest Msm homolog for Mtb DppA is Msm OppA. As *Hemophilus influenza* OppA binds heme [66], and the Opp transport system is similar to the Dpp transport system, it suggests that the Opp system plays a role in heme uptake.

As Mtb relies on host-acquired iron for survival and growth, iron acquisition pathways are attractive drug targets. Herein, we have greatly expanded the knowledge on Mtb FecB and FecB2. We have solved the structure of FecB and determined that FecB binds heme and cMB in both its ferric and apo forms with high nanomolar to low micromolar affinity. We have also shown that FecB interacts with known components of the siderophore-mediated iron acquisition pathway, and that it potentially plays a dual role in both siderophore export and import. Furthermore, we have solved the structure of FecB2 and confirmed that it preferentially binds heme over Fe-cMB. The FecB2 interaction partners firmly place FecB2 in the heme uptake pathway, and our study also suggests that FecB may work in concert with FecB2 to facilitate Mtb heme acquisition. Finally, we propose that the heme and siderophore-mediated iron acquisition pathways are differentially regulated depending on the availability of the host iron source, and future investigations will be critical to further our understanding of the regulatory mechanisms of iron acquisition in Mtb.

## Materials & methods

### Protein expression and purification

#### Cloning of Rv0265c (FecB2) and Rv3044 (FecB)

For structural studies, the C-terminal portion of *rv0265c* (*fecB2*) was amplified from Mtb H37Rv genomic DNA with primers (Rv0265c-For, and Rv0265c-Rev **S3 Table**) incorporating 5′ *Nco*I and 3′ *Xho*I restriction enzyme sites and cloned into pET28a (Novagen). The resulting construct contains an N-terminal methionine residue followed by the mature FecB2 protein sequence (Ala39-Ala330) and a C-terminal non-cleavable His-tag. As residues 1–38 of FecB encompass the predicted N-terminal signal peptide and lipoprotein attachment site, they were omitted from the expression construct. The sequence of the cloned gene was verified by DNA sequencing (GeneWiz, Piscataway, NJ).

For ligand binding studies, the same gene encoding the mature FecB2 (residues Ala39-Ala330) was subcloned into pET28a to produce a construct with a thrombin-cleavable N-terminal His-tag. The expressed protein after thrombin cleavage includes an additional GSHM sequence at its N-terminus. The primers (Rv0265c-Thrombin-For and Rv0265c-Thrombin-Rev, **S3 Table**) incorporate 5′ *Nde*I and 3′ *Hind*III restriction enzyme sites.

For both crystallography and ligand binding studies, the gene (*rv3044*) encoding FecB without the predicted signal peptide (encoding for residues Ala29-Asn359) was cloned into a pET28a vector from Mtb H37Rv genomic DNA to encode for a thrombin-cleavable N-terminal His-tag like the FecB2-construct for ligand binding studies. The primers (Rv3044-For and Rv3044-Rev, **S3 Table)** incorporate 5′ *Nde*I and 3′ *Hind*III restriction enzyme sites. The resulting mature FecB protein includes Ala29-Asn359 and following thrombin cleavage includes an extra GSHM sequence at the N-terminus.

For co-immunoprecipitation of *M*. *tuberculosis* proteins, *fecB* (*rv3044*) was synthesized with a C-terminal FLAG-tag (GenScript) and cloned via Gateway cloning technology (Invitrogen) under control of the hsp60 promoter on an episomal plasmid. *MmpS5* (*rv0677c*) was synthesized with a C-terminal HA-tag and expressed under control of a synthetic promoter (P750) on a plasmid that integrates into the attL5 site of the Mtb genome.

#### Expression and purification of FecB2 and FecB for crystallization

The FecB2 expression plasmid was transformed into *E*. *coli* BL21-CodonPlus (DE3)-RIL cells (Agilent, Santa Clara, CA) and cells were grown in LB media supplemented with 50 μg/mL kanamycin to an OD_600_ of 0.6 before protein expression was induced by the addition of 0.5 mM IPTG. Cell growth was continued overnight at 18°C and the cells harvested by centrifugation and stored at -80°C. Cell pellets were resuspended in buffer A (20 mM Tris, pH 8.0, 300 mM NaCl, 10% glycerol) containing a protease inhibitor cocktail (cOmplete, Roche), hen egg white lysozyme and 1mM PMSF, and the cells were lysed by sonication on ice (45% amplitude, 15 seconds on 45 seconds off, for 20 cycles). The lysate was centrifuged for 30 minutes at 30000 x *g* and the clarified supernatant was incubated with Ni-NTA agarose beads at 4°C for 2 hours. The suspension was poured into a gravity column, washed extensively with buffer B (buffer A with 20 mM imidazole), and the bound protein eluted with buffer C (buffer A with 300 mM imidazole). Eluted protein was concentrated and further purified by size exclusion chromatography using a HiLoad Superdex 200 column (GE Healthcare Life Sciences, Piscataway, NJ) equilibrated in buffer A. Protein purity was assessed by SDS-PAGE and fractions containing pure protein were pooled and concentrated to 10 mg/ml for crystallization screening.

A similar protocol was carried out for the expression and purification of FecB. However, the FecB expression plasmid was transformed into *E*. *coli* BL21-Gold (DE3) cells (Agilent, Santa Clara, CA), and protein expression was induced with 1mM IPTG.

#### Removal of the N-terminal His-tag for FecB and FecB2

Following Ni-NTA purification, FecB and FecB2 proteins were treated with the thrombin CleanCleave kit (Sigma) following manufacturer protocols to remove His-tags. Briefly, thrombin resin was added to the protein and incubated overnight at 4°C with stirring. The resin was then removed by centrifugation at 2,500 rpm for 5 minutes and the cleaved protein sample was incubated with Ni-NTA resin (HisPur, Thermo Scientific) for one hour at room temperature. The flow-through was collected and verified to contain cleaved FecB or FecB2 by SDS-PAGE and MALDI-TOF mass spectrometry.

#### Construction and purification of FecB and FecB2 variants

The FecB single variants, R141S, Q233S, R240S, Y242S, Y270S, E272S, D322S, Q336S and E339S, and the FecB2 single variants, Y39S, W58S and R184S were generated by *in vitro* site-directed mutagenesis using Pfu Ultra Fusion HS DNA polymerase (Agilent) with the primers listed in **S3 Table** and then confirmed by DNA sequencing (GeneWiz from Azenta Life Sciences).

Each mutant vector was transformed into *E*. *coli* BL21-Gold (DE3) cells and grown as above. The FecB2 mutants were purified as described above and underwent His-tag removal. The FecB mutants were all insoluble and were purified as follows. Cells were lysed by sonication on ice in lysis buffer (50 mM Tris-HCl pH 7.4, 350 mM NaCl, 10% glycerol, 10 mM imidazole) containing 10 μM PMSF and hen egg white lysozyme at 45% amplitude, 15 seconds on 45 seconds off, for 20 cycles. The crude cell lysate was then centrifuged at 14,000 rpm for 1 hour before discarding the supernatant. The pellet was then solubilized in 50 mM Tris-HCl pH 7.4, 350 mM NaCl, 10% glycerol, 6M urea overnight at 4°C. The resuspended lysate was then centrifuged at 14,000 rpm for 1 hour before being passed through a 1 μm syringe filter to remove insoluble debris. The urea was then sequentially removed by dialysis in buffers of decreasing urea concentration: 4 M urea, 2 M urea, and two rounds of 0 M urea. The re-folded protein was then loaded onto Ni-NTA resin (HisPur Ni-NTA Resin, Thermo Scientific) and eluted with a stepwise elution gradient of imidazole going up to 500 mM imidazole. Fractions found to contain FecB by SDS-PAGE analysis (expected MW ∼37 kDa) were pooled and dialyzed against 50 mM Tris-HCl pH 7.4, 350 mM NaCl, 10% glycerol overnight at 4°C. Successful refolding was confirmed by circular dichroism (CD). Far UV CD spectra were collected at 25°C using a Jasco J-810 spectropolarimeter using 0.1 cm cuvettes with FecB variant (5 μM) in 5 mM Tris pH 7.4, 35 mM NaCl, 1% glycerol. The bandwidth and wavelength step were set to 1 nm and the BeStSel tool (https://bestsel.elte.hu/index.php) was used to quantify secondary structural elements.

### Protein structure determination

#### Protein crystallization

Crystallization screening was performed with a Mosquito robot (SPT Labtech, Cambridge, MA) and commercially available crystallization screens by the hanging drop vapor diffusion method. Diffraction-quality FecB2 crystals were grown with a crystallization reservoir of 3.0 M ammonium sulfate, 95 mM citric acid pH 5.0 with a reservoir to protein drop ratio of 1:1. Single crystals were cryoprotected with a short soak in reservoir solution containing 20% glycerol and then flash frozen with liquid nitrogen.

To obtain FecB diffraction-quality crystals, the crystallization reservoir was 0.2 M NaCl, 0.1 M Phosphate-citrate pH 3.8, 26% w/v PEG 8000, with a reservoir to protein drop ratio of 2:1. Single crystals were flash frozen in 100% Paratone oil.

#### Structure solution and refinement

Data from a single FecB2 crystal was collected at beamline 24-ID-C of the Advanced Photon Source and processed with Denzo and Scalepack [67]. The Rv0265c structure was subsequently solved by molecular replacement with the program Phaser [68]. The search model was generated by threading the Rv0265c sequence onto the structure of *M*. *smegmatis* FecB (MSMEG_0438; PDB accession code 4MDY) using the Phyre server [69]. The structure was refined using buster-tnt [70] and model quality was assessed with PROCHECK [71], MolProbity [72], and the UCLA-DOE SAVES server (http://nihserver.mbi.ucla.edu/SAVES/). Figures were prepared using PyMOL [73] and electrostatic surfaces were generated using APBS [73] and the PDB2PQR server [74].

Data from a single FecB crystal was collected at ALS beamline 8.2.1, and was indexed, integrated, and scaled using in iMOSFLM [75] and Aimless [76]. Initial phases for the FecB structure were determined by molecular replacement using the Phaser program [68] from the PHENIX suite using the *B*. *cereus* PBP, YfiY (PDB ID 3TNY), as the search model. Model building was carried out by Autobuild in the PHENIX [77], and followed by iterative rounds of structure refinement in Coot [78] and phenix.refine [79].

### Preparation and purification carboxymycobactin

#### Preparation and purification of Fe-cMB

Ferric-carboxymycobactin (Fe-cMB) was purified from Msm growth media as previously described [80] using an MsmΔ*fxbA* variant [81], a generous gift from Eric Rubin residing at the Harvard School of Public Health. Briefly, a culture of Msm*ΔfxbA* was streaked on 7H10 agar plates containing 50 μg/mL hygromycin B and grown for 3 days at 37°C. A colony was then inoculated into 7H9 media containing 10% ADC supplement and 50 μg/mL hygromycin B and grown for 5 days at 37°C. The resulting culture was then inoculated 1:1,000 into minimal media containing 5 g KH_2_PO_4_, 5 g L-asparagine, 60 mL of glycerol, and the pH was adjusted to 7.0 in a final volume of 1 L. After autoclaving, syringe-filtered (0.2 μm filter, Minisart Plus, Sartorius) MgSO_4_ (final concentration 1.7 mM), ZnSO_4_ (6.9 μM) and MnSO_4_ (1.8 μM) were added with 50 μg/mL hygromycin B and grown for another 5 days at 37°C. Finally, the culture was inoculated 1:100 into fresh minimal media as described above with the addition of 0.08 μg/mL FeSO_4_ and grown for a final 5 days at 37°C. To isolate Fe-cMB, the bacteria were centrifuged at 5,000 rpm for 30 minutes and the resulting supernatant was filtered with a 0.2 μm filter. The media was then acidified to a pH of 3.5 with HCl before dropwise addition of 10% FeCl_3_ (w/v) in 100% ethanol until the formation of a reddish-brown precipitate (FePO_4_) was observed. The solution was then stirred for 1 hour at room temperature before centrifugation at 5,000 rpm for 30 minutes and the resulting supernatant was filtered with a 0.2 μm filter. Fe-cMB was then extracted into 1 volume of ethyl acetate. The organic later was washed two times with water, dried over anhydrous MgSO_4_, and filtered using filter paper (Whatman) before being dried by rotary evaporation. The resulting crude Fe-cMB extract was then purified via HPLC (Beckman Coulter, System Gold) on an Ultrasphere ODS 5 μm 80 Å C18 Column (150 × 10 mm, Hichrom) at a flow rate of 2 mL/min. The mobile phase consisted of Solvent A: Water:0.1% FA, and Solvent B: ACN:0.1% FA. A wash was run for 6 minutes at 36% B before a linear gradient was run starting at 36% B and ending at 96% B over 60 minutes, and then holding at this concentration for a further 20 minutes. Fe-cMB retention times were 11.5 minutes, 12.7 minutes, 14.0 minutes, 15.8 minutes, 18.5 minutes, and 21.7 minutes for the different chain lengths of Fe-cMB produced (n = 3–8). Fe-cMB were pooled based on their characteristic absorbance at 450 nm and lyophilized before being stored at -20°C.

#### Removal of iron and isolation of apo-cMB

Fe-cMB was converted to its desiferri form as previously described [82]. Briefly, HPLC-purified Fe-cMB was incubated with 50 mM EDTA, pH 4, for one hour at room temperature. The resulting desferri-cMB (cMB) was then separated from EDTA using solid-phase extraction with a Sep-Pak C18 cartridge (Waters) equilibrated with 2 CV each of: acetonitrile (ACN), water, 0.1% trifluoroacetic acid (TFA), and methonal:0.1% TFA (10:90). After sample application the column was washed with 4 CV each of 0.1% TFA, and ACN:0.1% TFA (20:80), and then 2 CV of methanol:0.1% TFA (50:50) and eluted with 1.5 CV of methanol and lyophilized and stored at -20°C. The cMB was then solubilized in 50 mM Tris-HCl pH 7.4, 150 mM NaCl. To determine concentration, a portion of the solution was ferrated by adding an excess of FeCl_3_ and then analyzed by UV/vis spectroscopy (ε_450_ = 3,800.16 M^-1^ cm^-1^).

### Fluorescence titrations

Fluorescence quenching titrations of heme, ferric-carboxymycobactin (Fe-cMB), ferric-enterobactin, ferric-pyoverdines and ferric-mycobactin J (Fe-MBJ) were performed as described previously [49,52]. Stock solutions of FecB or FecB2 (100 nM), heme (100 μM), Fe-cMB (80 μM), and Fe-Pyo (1 mM) were prepared in 50 mM Tris-HCl pH 7.4, 150 mM NaCl. Stock solutions of Fe-Ent (1 mM), Fe-MBJ (250 μM), and PPIX (230μM) were prepared in the same buffer with the addition of 10% DMSO. All small molecules besides Fe-cMB were added in the following titration series: 2 x 1 μL, 4 x 2 μL, 4 x 5 μL, 3 x 10 μL, and 2 x 20 μL injections, while Fe-cMB followed the same titrations without the final three injections. In between each titration the solution was incubated for 3 minutes with stirring at 200 rpm at 20°C. Representative raw and corrected fluorescence spectra for each experiment is provided in **S14 Fig**. Fluorescence spectra were acquired between 300–500 nm using a Hitachi F-7100 Fluorescence Spectrophotometer through excitation at 285 nm with the following settings: PMT voltage of 950 V, excitation slit width of 2.5 nm, emission slit width of 5.0 nm, and a scan speed of 240 nm/min.

#### Fluorescence emission spectral analysis

Results from the fluorescence-based assay were fit to Eq 1 derived from [52], to determine the equilibrium dissociation-constant (*K*_*d*_) of heme or Fe-cMB with FecB2 or FecB and its mutants.


$$F\;=\;\frac{\left([FecB]\;+\;[ligand]\;+\;K_{d}\right)\;-\;\sqrt{{\left([FecB]\;+\;[ligand]\;+\;K_{d}\right)}^{2}\;-\;4[FecB][ligand]}}{2}\;\times \;\left(\frac{F_{min}-F_{max}}{\left[FecB\right]}\right)\;+\;F_{max}$$ (1)

In Eq 1, [FecB] is the total concentration of FecB or FecB2 or mutants, [ligand] is the total concentration of heme or Fe-cMB, *F*_max_ is the emission intensity without ligand, and *F*_min_ is the emission intensity for fully ligand-bound FecB(2). Fitting of the fluorescence emission intensity at 335 nm for *K*_*d*_ determination was performed using GraphPad Prism (Ver 9.3.1).

### *M*. *smegmatis* co-IP and protein interaction partner identification

#### Cloning of Msm fecB, fecB2, mmpS4 and mmpS5 genes

Msm *fecB* (MSMEG_2319), *fecB2* (MSMEG_0438), *mmpS4* (MSMEG_0380) and *mmpS5* (MSMEG_0226) coding sequences were amplified from Msm mc^2^155 genomic DNA using Pfu Ultra Fusion HS DNA polymerase (Agilent) using recommended components and PCR cycle conditions. All these genes were expressed from the constitutive Ptb38 promoter [56] and carried a FLAG epitope coding sequence at the 3’ end.

Msm *fecB* (MSMEG_2319) and Msm *fecB2* (MSMEG_0438) were cloned into the KanR mycobacterial expression vector pDE43-MEK using the Gateway cloning technology (Invitrogen), and Msm *mmpS4* (MSMEG_0380) and Msm *mmpS5* (MSMEG_0226) genes were assembled into a HygR mycobacterial expression plasmid using the NEBuilder HIFI DNA assembly (New England Biolabs). Appropriate control vectors lacking the coding sequences were prepared by restriction digestion and cloning in a set of annealed oligos. The expression plasmids and the control vectors were electroporated into Msm mc^2^155 and selected on 7H10 plates supplemented with 25 μg/ml kanamycin or 50 μg/ml hygromycin, as appropriate. This description of plasmids and the list of oligos used in this study are listed in **S3 Table.**

#### Co-IP of FLAG-tagged Msm constructs

Msm variants were streaked on Middlebrook 7H10 agar plates supplemented with 10% albumin/dextrose/catalase (ADC), 0.05% Tween-80, and 50 μg/mL kanamycin and grown for 3 days at 37°C. For FecB2-FLAG, a single colony was then used to inoculate growth cultures in Middlebrook 7H9 media supplemented with 10% ADC, 0.05% Tween-80, and 50 μg/mL kanamycin and grown for 2 days at 37°C before the cells were harvested by centrifugation at 5,000 rpm for 20 mins, and then washed twice with TBS (Tris-Buffered Saline, 50 mM Tris-HCl pH 7.4 + 150 mM NaCl, at which they were stored at -20°C until the co-IP was performed. For all other constructs a single colony was used to inoculate growth cultures in Middlebrook 7H9 media supplemented with 10% ADC, 0.05% Tween-80, and 50 μg/mL kanamycin and grown for 2 days at 37°C before the cells were harvested by centrifugation at 5,000 rpm for 20 mins, and then washed twice with TBS (Tris-Buffered Saline, 50 mM Tris-HCl pH 7.4 + 150 mM NaCl) and then were resuspended in iron-free 7H9 media supplemented with 10% ADC, 0.05% Tween-80, and 50 μg/mL kanamycin and grown for an additional 2 days at 37°C to deplete intracellular iron levels. The media was then supplemented with 20 μM ferric ammonium citrate and grown for a final 2 days at 37°C before harvesting the cells by centrifugation at 5,000 rpm for 25 minutes. The cells were then resuspended and lysed in buffer (1 mL per 25 mg cell mass, 50 mM Tris-HCl pH 7.4, 350 mM NaCl, 10% glycerol) by sonication (60% amplitude, 15 seconds on, 45 seconds off, 10 cycles). N-dodecyl-β-D-maltoside, a mild detergent used for membrane protein research, was then added to a concentration of 1% w/v and incubated for 2 hours at 4°C. To remove cellular debris the lysate was centrifuged at 14,000 rpm for 30 minutes and syringe filtered with a 0.1 μm filter. FLAG-tagged FecB and potential interacting partners were then precipitated using ANTI-FLAG M2 affinity gel slurry (Sigma) following manufacturer protocols. Briefly, 40 μL of resin slurry and lysate were incubated overnight at 4°C before centrifuging for one minute at 2,500 rpm and washed 2x with 500 μL of TBS. The proteins were then eluted from the slurry by treating with 100 μL of 1% formic acid and heating at 95°C for 10 minutes. The supernatant was then neutralized by adding 400 μL of 50 mM ammonium bicarbonate. The proteins were then treated with 0.5 mg/mL dithiothreitol for 30 minutes at 80°C, followed by treatment with 0.5 mg/mL iodoacetamide for one hour in the dark. The proteins were then digested using 1.5 ng/μL of trypsin and incubated overnight at 37°C. The peptides were then de-salted using Sep-Pak C18 cartridges (Waters) following manufacturer protocols to prepare them for mass spec analysis.

#### Mass spectrometry data acquisition

Proteomics data were acquired via liquid chromatography (LC)-MS/MS using an UltiMate 3000 UHPLC (Thermo Fisher Scientific) coupled in-line with an Orbitrap Fusion Lumos mass spectrometer (Thermo Fisher Scientific) using a Nanospray Flex ion source. Mobile phase A is comprised of 0.1% FA in water, while mobile phase B is 0.1% FA in ACN. The total flow rate was 300 nL min^-1^, and the C18-cleaned peptides were separated over a 57 min gradient from 4% to 25% buffer B (total run time is 90 min) on an Acclaim PepMap RSLC column (50 cm x 75 μm). Survey (MS1) scans were acquired in Orbitrap (FT) with automated gain control (AGC) target 1E6, maximum injection time 50 ms, and dynamic exclusion of 60 s after 2 selections across the scan range of 375–1800 m/z. MS/MS spectra were acquired in data-dependent acquisition mode at top speed for 3 s per cycle. The AGC target was set to 1E4 with maximum injection time of 35 ms. Ions were subjected to stepped-energy higher-energy collision dissociation (seHCD) fragmentation at a normalized collision energy (NCE) of 20±5%.

#### Protein identification using MaxQuant

The raw LC-MS/MS data files were analyzed using MaxQuant (version 1.5.2.8), with the spectra searched against the Uniprot *M*. *smegmatis* database (updated June 2018). For identification of the peptides, the mass tolerances were 20 ppm for initial precursor ions and 0.5 Da for fragment ions. Two missed cleavages in tryptic digests were allowed. Cysteine residues were set as static modification. Oxidation of methionine was set as the variable modification. Filtering for the peptide identification was set at a 1% false discovery rate (FDR).

### Mtb co-IP and Western protocols

#### Co-IP of FLAG- or HA-tagged Mtb proteins

150 mL of MtbΔ*fecB* [39] culture transformed with pGMEK-P_hsp60_-*fecB*-FLAG and pGMCS-P_750_-*mmpS5*-HA was grown in roller incubator until the culture reached OD_580_ = 1. The bacteria were collected by centrifugation at 4000 rpm, 8 min, 4°C, followed by resuspension in 20 mL PBS 0.05% Tween 80. After a second centrifugation step (4000 rpm, 8 min, 4°C), the pellet was resuspended in 1.2 mL of lysis buffer (50 mM Tris-HCl pH7.4, 50 mM NaCl) with protease inhibitor (Roche cOmplete, Mini, EDTA-free protease inhibitor cocktail). 600 mL of suspension was bead-beaten with ∼”300 mL” of zirconia beads 4 times for 30 seconds each, with chilling on cold rack between each round. Lysates were centrifuged at 11,000 x g, 5 min, 4°C to remove unbroken cells and insoluble material. An aliquot of the lysate fraction was saved for Western-blot and the rest was added either to 50 mL slurry of “FLAG” magnetic agarose (Thermo Scientific Pierce Anti-DYKDDDDK Magnetic Agarose) that had been pre-washed in lysis buffer according to the manufacturer’s protocol, or to 25 mL of HA magnetic beads (Thermo Scientific Pierce Anti-HA Magnetic Beads) that had been pre-washed in 0.05% TBS-T (Tris-Buffered Saline, 50 mM Tris-HCl pH 7.4 + 150 mM NaCl, with 0.05% of Tween 20) according to the manufacturer’s protocol. Lysates were incubated with magnetic agarose/beads for 1 hr at room temperature on a rotating platform. After incubation, the flow-through fraction was collected using a magnetic stand and subsequent washing steps were performed according to the magnetic agarose/bead manufacturer’s protocol. The elution of immunoprecipitated proteins was performed first with addition of (i) 1.5 mg/mL Pierce 3x DYKDDDDK Peptide (in PBS) to “FLAG” magnetic agarose, or (ii) 2 mg/mL Pierce HA Peptide (in TBS) to HA magnetic beads, which were incubated for 10 min at room temperature on a rotating platform. Further recovery of immunoprecipitated proteins was obtained by resuspending magnetic agarose/beads in PBS with 4x Laemmli sample buffer (Bio-Rad) containing 2-mercaptoethanol and boiling for 10 min at 95°C. 1:4 dilution of 4x Laemmli sample buffer containing 2-mercaptoethanol was also added to all other fractions (lysate, flow-through and eluate) which were also boiled for 10 min at 95°C. Samples were stored at -80°C prior to running the western-blots to detect the proteins of interest.

#### Western-Blot to detect FLAG- and HA-tagged proteins

Samples were thawed and boiled at 95°C for 10min prior to loading on a 4–20% precast polyacrylamide gel (Bio-Rad Mini-PROTEAN Precast Protein Gels). After running at 100 V for 60 min, proteins were transferred to a nitrocellulose membrane (Invitrogen iBlot 2 Transfer Stacks) in an iBlot2 dry blotting system (Invitrogen). Membranes were blocked with Intercept (TBS) Blocking Buffers (Li-Cor) for 1 hr at room temperature, followed by incubation with primary antibodies anti-FLAG (monoclonal anti-FLAG M2, Sigma, at 1:800) or anti-HA (HA Tag Monoclonal Antibody 2–2.2.14, Invitrogen, at 1:10,000) or polyclonal anti-FecB antiserum (at 1:5000 dilution; produced by Thermofisher) in dilution buffer (1:1 PBS:Intercept buffer plus 0.1% Tween 20) on a rocking platform, at 4°C, overnight. After washing the primary antibody with PBS 0.1% Tween 20 three times, membranes were incubated with a fluorescent secondary antibody (Goat anti-Mouse IgG (H+L) Cross-Adsorbed Secondary Antibody DyLight800, Thermo Fisher, or IRDye 680LT Donkey anti-rabbit, Thermo Fisher at 1:10,000 in dilution buffer) for 1h at room temperature on a rocking platform. After three washes with PBS 0.1% Tween 20, the membranes were visualized using the Azure 600 Imaging System.

## Supporting information

S1 Fig Alignment of *M*. ***tuberculosis* (Mt) FecB and FecB2 with structural homologs.** A sequence alignment of FecB and FecB2 homologs based on both sequence and secondary structure elements were generated using Clustal Omega and Dali. A cartoon depicting the secondary structure elements of Mtb FecB is shown above the sequence alignment. Conserved residues are indicated in bold, with siderophore or heme coordinating residues highlighted in red. Siderophore coordinating residues that are known to be critical for siderophore binding are shown in yellow text. Residues that are known to interact with a protein partner are highlighted in blue. Structural homologs are included from *M*. *smegmatis* (Ms-FecB2, PDB ID 4MDY), *E*. *coli* (Ec-FitE, 3BE6), *Staphylococcus pseudintermedius* (Sp-FhuD, 5FLY), *Streptococcus pneumoniae* (Sp-PiaA, 4HMQ), *Staphylococcus aureus* (Sa-HtsA, 3LI2; Sa-SirA, 3MWF; Sa-FhuD2, 4FNA; Sa-IsdE, 2Q8Q), *Bacillus anthracis* (Ba-FpuA, 6ALL), *Bacillus cereus* (Bc-YfiY, 3TNY), *Bacillus subtilis* (Bs-FeuA, 3HXP; Bs-FhuD, 2WHY; Bs-YclQ, 3GFV), *Corynebacterium glutamicum* ATCC 13032 (Cg-HmuT, 5AZ3), and *Shigella dysenteriae* (Sd-ShuT, 2R7A).
(PDF)

S2 Fig Secondary structure assignments for FecB and FecB2. FecB (**A**) and FecB2 (**B**) are colored by secondary structure where α-helices and β-strands, respectively, are colored blue and yellow for FecB and green and pink for FecB2. N- and C-termini are labeled in red. FecB and FecB2 are shown with two identical 180**°** orientations.
(PDF)

S3 Fig Close-up of the ligand binding pocket of FecB and FecB2, along with interacting residues of PEG observed in the FecB crystal. (A and B) Left panels: (**A**) FecB (green) and (**B**) FecB2 (orange) are shown as cartoon depictions and potential ligand binding residues shown as white sticks. Right panels: APBS (Adaptive Poisson-Boltzmann Solver ‐ a PyMol plugin) generated electrostatic surfaces of the ligand binding sites of FecB and FecB2. With negatively and positively charged molecular surfaces colored in red and blue, respectively. (**C**) FecB crystallized with a PEG molecule (pink stick) observed in the ligand-binding pocket. Polar interactions within 4 Å between FecB residues and PEG are indicated with dashed black lines, coordinating residues are shown as green sticks, and a coordinating water molecule is shown as a red sphere. Arg240, Tyr242 and Tyr270 are shown for comparison to Fig 3.
(PDF)

S4 Fig Molecular structures of heme and siderophores. A and B represent molecular structures of heme and the apo- and ferric-siderophore respectively, and C represents heme bound to Sa-IsdE (PDB 2Q8Q) and ferric-siderophores bound to PBPs as in staphlyoferrin A bound to Sa-HtsA (PDB, 3LI2), staphyloferrin B bound to Sa-SirA (PDB, 3MWF) and schizokinen bound to Bc-YfiY (PDB, 3TNY) and carboxymycobactin S bound to siderocalin (PDB, 1X89). Depcitions were produced in LigPlot.
(PDF)

S5 Fig Comparison of heme affinity of FecB and FecB2. Representative fluorescent emission intensities at 335 nm after excitation at 280 nm of 100 nM FecB (left panels) and 100 nM FecB2 (right panels) with increasing concentrations of heme. Experiments were performed with (**A**) and without (**B**) the HisTag. Curves were fit using the equation in the methods and heme affinities (K_d_) are included for each titration.
(PDF)

S6 Fig Comparison of Fe-cMB affinity of FecB and FecB2. Representative fluorescent emission intensities at 335 nm after excitation at 280 nm of 100 nM FecB (left panels) and 100 nM FecB2 (right panels) with increasing concentrations of Fe-cMB. Experiments were performed with (**A**) and without (**B**) a HisTag. Curves were fit using the equation in the methods and Fe-cMB affinities (K_d_) are included for each titration. Notably, no titration was carried out for FecB2 without a Histag, as Fe-MB bound so poorly to FecB-HisTag.
(PDF)

S7 Fig Circular dichroism (CD) spectra of FecB and its variants. CD was performed to ensure that no major structural changes occurred with the generation of FecB variants compared to wild-type (WT) FecB. Experiments were performed at 25°C using a Jasco J-810 spectropolarimeter. WT and variant FecB samples (5 μM) were analyzed in 5 mM Tris pH 7.4, 35 mM NaCl, 1% glycerol.
(PDF)

S8 Fig Heme affinity of FecB in its native and refolded forms. Representative fluorescent emission intensities at 335 nm after excitation at 280 nm of WT-FecB with increasing concentrations of heme. Titrations were performed with WT-FecB (100 nM) purified in its (**A**) native, soluble form and (**B**) refolded form. Curves were fit using the equation in the methods and heme or Fe-cMB affinities (K_d_) are included for each titration.
(PDF)

S9 Fig Determination of apo-cMB and ferric-siderophores affinity of FecB. Representative fluorescent emission intensities at 335 nm after excitation at 280 nm of FecB with increasing concentrations of apo-cMB or Fe-siderophores. (**A**) Apo-cMB affinity to WT- and E339S-FecB variant tested. (**B**) Affinity of ferric-MBJ for FecB. (**C**) Affinity of Fe-pyoverdines (Fe-pyo) and Fe-enterobactin (Fe-EB) for FecB. Curves were fit using the equation in the methods and affinities (K_d_) are included for each titration where possible.
(PDF)

S10 Fig Determination of Fe-cMB affinity of FecB ligand-binding pocket variants. Representative fluorescent emission intensities at 335 nm after excitation at 280 nm of FecB variants with increasing concentrations of Fe-cMB. FecB variants tested for Fe-cMB affinity include FecB L135R, R141S, Q233S, R240S, Y242S, Y270S, E272S, D332S, Q336S, and E339S mutations, along with the double mutants FecB R240S-E339S and Y242S-E339S. Curves were fit using the equation in the methods and Fe-cMB affinities (K_d_) are included for each titration.
(PDF)

S11 Fig Determination of heme affinity of FecB2 ligand-binding pocket variants. Representative fluorescent emission intensities at 335 nm after excitation at 280 nm of FecB2 variants with increasing concentrations of heme. FecB2 variants tested for heme affinity include FecB L135R, R141S, Q233S, R240S, Y242S, Y270S, E272S, D332S, Q336S, and E339S mutations, along with the double mutants R240S-E339S and Y242S-E339S. Curves were fit using the equation in the methods and Fe-cMB affinities (K_d_) are included for each titration.
(PDF)

S12 Fig FecB-FLAG has a processed signal peptide while FecB2 does not. **(A)** Peptides that were identified by MS (highlighted in yellow) results in 87% coverage of full-length FecB. The remaining residues not identified by MS are the first 42 residues, suggesting that FecB is processed and translocated to the periplasmic and cell-wall environments. **(B)** In the case of FecB2-FLAG, there is 99% coverage of the full-length FecB2 sequence, suggesting that the signal peptide is not processed.
(PDF)

S13 Fig Western Blot analysis of Mtb co-IPs suggest that FecB interacts with MmpS5. Western Blot analysis of protein co-IPs with MtbΔ*fecB* with vectors expressing FecB-FLAG and MmpS5-HA, and the negative control whereby Mtb contains vectors that express the tags alone. Western Blot analysis of both the boiled beads and eluate of the (**A**) co-IP using the anti-FLAG beads to pull-down FecB-FLAG, probed with anti-FLAG (left panel) and anti-HA (right panel) antibody that recognizes FecB-FLAG andMmpS5-HA (respectively), (**B**) co-IP using the anti-HA beads to pull down MmpS5-HA, probed with an anti-FLAG (left panel) and anti-HA (right panel) antibody that recognizes FecB-FLAG and MmpS5-HA (respectively), and (**C**) co-IP using the anti-FLAG (left panel) and anti-HA beads (right panel) to pull down FecB-FLAG and MmpS5-HA (respectively), probed with anti-FecB antiserum that recognizes FecB. This figure shows all steps that generated **Fig 6**, so includes all lysates, washes, boiled beads and eluents.
(PDF)

S14 Fig Raw data and difference spectra for tryptophan fluorescence experiments. Representative titration experiments for FecB with ferric-carboxymycobactin (Fe-cMB) and heme. Raw data includes buffer fluorescence alone, and matched titrations of ligand into buffer (Fe-cMB spectrum) or into FecB (FecB raw spectrum). To attain the FecB difference spectrum, one must measure the three forms of raw data and subtract buffer and ligand signal from the protein-ligand experiment. The difference spectrum indicates fluorescence changes due to interactions between ligand and protein, as noise from ligand alone and buffer has been subtracted. The data from difference spectra is then plotted and fit to determine the affinity of the protein for its ligand.
(PDF)

S1 Table DALI server structural homology results for FecB and FecB2. (DOCX)

S2 Table Quantification of Secondary Structure Elements from Circular Dichroism with FecB variants. (DOCX)

S3 Table List of primer sequences used in this study. (DOCX)

S4 Table List of plasmids used in this study. (DOCX)

S1 Data MS/MS protein results for the FecB-FLAG co-PI experiment. (XLSX)

S2 Data MS/MS protein results for the MmpS5-FLAG co-PI experiment. (XLSX)

S3 Data MS/MS protein results for the MmpS5-FLAG co-PI experiment. (XLSX)

S4 Data MS/MS protein results for the FecB2-FLAG co-IP experiment. (XLSX)
