# Supplementary material for: Differentiating the roles of Mycobacterium tuberculosis substrate binding proteins, FecB and FecB2, in iron uptake
Source: PLoS Pathog. 2023 Sep 25;19(9):e1011650. doi: 10.1371/journal.ppat.1011650 (PMC10553834; doi:10.1371/journal.ppat.1011650)
Supplement: S1 Fig — tuberculosis (Mt) FecB and FecB2 with structural homologs. A sequence alignment of FecB and FecB2 homologs based on both sequence and secondary structure elements were generated using Clustal Omega and Dali. A cartoon depicting the secondary structure elements of Mtb FecB is shown above the sequence alignment. Conserved residues are indicated in bold, with siderophore or heme coordinating residues highlighted in red. Siderophore coordinating residues that are known to be critical for siderophore binding are shown in yellow text. Residues that are known to interact with a protein partner are highlighted in blue. Structural homologs are included from M. smegmatis (Ms-FecB2, PDB ID 4MDY), E. coli (Ec-FitE, 3BE6), Staphylococcus pseudintermedius (Sp-FhuD, 5FLY), Streptococcus pneumoniae (Sp-PiaA, 4HMQ), Staphylococcus aureus (Sa-HtsA, 3LI2; Sa-SirA, 3MWF; Sa-FhuD2, 4FNA; Sa-IsdE, 2Q8Q), Bacillus anthracis (Ba-FpuA, 6ALL), Bacillus cereus (Bc-YfiY, 3TNY), Bacillus subtilis (Bs-FeuA, 3HXP; Bs-FhuD, 2WHY; Bs-YclQ, 3GFV), Corynebacterium glutamicum ATCC 13032 (Cg-HmuT, 5AZ3), and Shigella dysenteriae (Sd-ShuT, 2R7A). (PDF) [file ppat.1011650.s001.pdf]

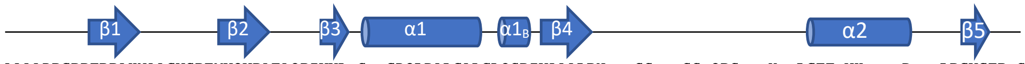


```
Mt-FecB   ----------------------AAAADPGPPTRPAHNAAGVSPEMVQVPAEAQRIVVL-S--GDQLDALCALGLQSRIVAAALPN---SS---SS-QPS---Y--LGTT-VH----D---LPGVGTR-S 142
Mt-FecB2  ------------------------------AAVTITHL----FGQTVIKEPPKRVVSA-G--YTEQDDLLAVDVV--PIAVTDWF---GD---QPFAVW---P-WAAPK-L--GGAR---PAVLNL-DN 86
Ms-FecB2  ---------------------------DDGSVTVRHA-----FGDTTIPGPPQRVVSA-G--LTEQDDLLAVGVV--PIAVTDWF---GG---EPFGVW---P-WAQRQ-L--AGAQ---PAVLNL-DN 107
Ec-FitE   ----------------------------EPVQVFTDDL----GRKVTVPAHPKRIVSL-H-DLDITIPLIELGVP--PVASHGRT---RPDGSHF-IRS-GAL--LTGV-DF----DNSSIAFIGT--A 97
Sp-PiaA   -------------------------EHAPDKIVLDHA-----FGQTILDKKPERVATI-A--WGNHDVALALGIV--PVGFSKANYGVSA---DK-GVL---P-WTEEK-IKELNGK---ANLFDDL-D 114
Sa-SirA   ------------------------------TTSIKHAM-----GTTEIKGKPKRVVTL-Y--QGATDVAVSLGVK--PVGAVESW---TQ---KP-KFE---Y--IKND-LK----D---TKIVGQE-P 104
Sa-HtsA   ----------------------------ASTISVKDEN-----GTVKVPKDAKRIVVL-E--YSFADALAALDVK--PVGIADD-----G---KK-KRI---IKPVREK-IG----D---YTSVGTR-K 105
Bc-YfiY   ------------------------------EVVTVEHAM----GKTEVPANPKRVVIL-T--NEGTEALLELGVK--PVGAVKSW---TG---DP-WYP---H--IKDK-MK----D---VKVVGDE-G 70
Ba-FpuA   -------------------------------ITIKHAE-----GETKLDKPAKKVVVL-E--WVYSEDLLALGVQ--PVGMAD---------IK-NYNKWVN--TKTK-PS----K--DVVDVGTR-Q 104
Bs-FhuD   ------------------------------KKKIEYLD-----KTYEVTVPTDKIAITGS--VESMEDAKLLDVH--PQGAISF-----S---GK-FPD---M--FKDI-TD----K---AEPTGEK-M 85
Sp-FhuD   -------------------------------TKAFNLKTAKGEEKIDIPKDPKRIVVM-A--PTYAGGLKYLDA--NIVGVSDQ---------VD-QSP---V--LAKQ-FK----D---VDKVGA--- 71
Sa-FhuD2  ------------------------------ETKSYKMDD---GKTVDIPKDPKRIAVV-A--PTYAGGLKKLGA--NIVAVNQQ---------VD-QSK---V--LKDK-FK----G---VTKIG---- 92
Bs-YclQ   -----------------------------KEQITVKHQ--LDKNGTKVPKNPKKVVVF-D--FGSLDTLDKLGLDDIVAGLPKQ---------V--LPK---Y--LS-K-FK----D-DKYADVGSL-K 84
Bs-FeuA   ----------------------------------YKAE----NGNVKIPKHPKRVVVM-A--DGYYGYFKTLGI--NVVGAPEN-----------VFKN--PY--YKGKTNG----V---ENIGDG--- 105
Sa-IsdE   --------------------------------------------------GEFRIVPT-T--VALTMTLDKLDLP--IVGKPT------------SYKT------LPNR-YK----D---VPEIGQP-M 78
Cg-HmuT   DPRTFTGLSIVEDIGDVVPVTDNASPA---LPVSLTDAD---GNDVVVE-DVSRILPL-DLYGTYSKTIAGLGLVDNIVGRTVS-------------ST---E---P--A-LA----D---TEVVTTGGH 141
Sd-ShuT   --------------------------------------------------AAERIVVA-G--GSLTELIYAMGAGERVVGVDET-------------TS---Y--PP-E-TA----K---LPHIGYW-K 69
```

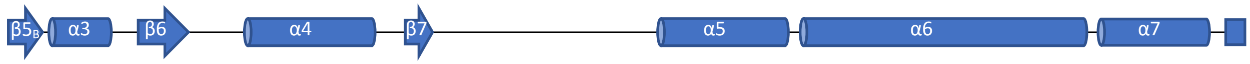


```
Mt-FecB   APDLRAIAAAHPDLILGS---QGLT-P-Q-LYPQLA-AIAPTVFT----A---AP-----GA-----DW-ENNLRGVGAATARIAAVDALIT---GFAEHATQVGT-----KHD--A---TH--FQASI 231
Mt-FecB2  GIQIDRIAALKPDLIVAI---NAGV-DAD-TYQQLS-AIAPTVAQSGGDA---FF-----E------PW-KDQARSIGQAVFAADRMRSLIE---AVDQKFAAVAQ-----RHP-RW---RG--KKALL 180
Ms-FecB2  GIPVEEIAALKPDLIVAT---NAGL-DAD-TYAKLS-EIAPTVAQTGSEA---FF-----E------PW-KDQATIIGQAVFKNAEMTELIK---SVDDRFTTVKT-----DHP-QF---SG--KKALL 201
Ec-FitE   DIDIEAIVAAKPDLIITE---P-TR-N-T-PIERLE-KIAPTVSI----D---H----------LKGGA-PEIYRKLAELTGTQSQLAILER---RYQAQINALKA-----TLD--S---QK--ITVSV 185
Sp-PiaA   GLNFEAISNSKPDVILAG---YSGI-TKE-DYDTLS-KIAPVAAY----KSKPWQ-----TL------W-RDMIKIDSKALGMEKEGDELIK---NTEARISKELEKHPEIKGK--I---KG--KKVLF 211
Sa-SirA   APNLEEISKLKPDLIVAS---KVRN-E-K-VYDQLS-KIAPTVST----DT-VF-------------KF-KDTTKLMGKALGKEKEAEDLLK---KYDDKVAAFQK-----DAKAKYKDAWP--LKASV 197
Sa-HtsA   QPNLEEISKLKPDLIIAD---SSRH-K-G-INKELN-KIAPTLSL----K--SFD-----GD---YKQN-INSFKTIAKALNKEKEGEKRLA---EHDKLINKYKD-----EIK--F--DRN--QKVLP 198
Bc-YfiY   QVNVETIASLKPDLIIGN---KMRH-E-K-VYEQLK-AIAPTVFS----E---TL-----RG-----EW-KDNFKFYAKALNKEKEGQKVVA---DYESRMKDLKG-----KLG-DK---VN--QEISM 160
Ba-FpuA   QPNLEEISRLKPDLIITA---SFRG-K-A-IKNELE-QIAPTVMF----D---PSTSNNDHF-----AEMTETFKQIAKAVGKEEEGKKVLA---DMDKAFADAKA-----KIE--KADLKD--KNIAM 201
Bs-FhuD   EPNIEKILEMKPDVILAS----TKF-P-EKTLQKIS-TAGTTIPV--------SH-----IS----SNW-KENMMLLAQLTGKEKKAKKIIA---DYEQDLKETKT-----KINDKA---KD--SKALV 176
Sp-FhuD   E-DVEKVASLKPDLIITY----NTD-K---NTDKLK-KIAPTIAF----D---Y----------AKYNY-LEQQEAMGDIVGKSDEVKKWKA---DWEKQTAQDSK-----DIK--AHLGDD--TSVTI 160
Sa-FhuD2  DGDVEKVAKEKPDLIIVY---S--T-D-K-DIKKYQ-KVAPTVVV----D---YN-----KH-----KY-LEQQEMLGKIVGKEDKVKAWKK---DWEETTAKDGK-----EIK--KAIGQD--ATVSL 182
Bs-YclQ   EPDFDKVAELDPDLIIIS----ARQ-S-E-SYKEFS-KIAPTIYL--------GV-----DTAKYMESF-KSDAETIGKIFDKEDKVKDELA---NIDHSIADVKK-------T--A---EKLNKNGLV 176
Bs-FeuA   -TSVEKVIDLNPDLIIVW---TTQG---A-DIKKLE-KIAPTVAV----K---YD-----KL-----DN-IEQLKEFAKMTGTEDKAEKWLA---KWDKKVAAAKT-----KIKKAV---GD--KTISI 194
Sa-IsdE   EPNVEAVKKLKPTHVLSVSTIKDEM-Q-P-FYKQLN-M---KGYF----Y--DF------DS---LKGM-QKSITQLGDQFNRKAQAKELND---HLNSVKQKIEN-----KAA---KQKKH--PKVLI 171
Cg-HmuT   TLNAEAILNLHPTLVIID---HSIGPR-E-VIDQIRAAGVATVIM----S---PQ-----RS---IASI-GDDIRDIASVVGLPEEGEKLAERSVAEVEEASTVVD-----ELT----PEDP--LKMVF 238
Sd-ShuT   QLSSEGILSLRPDSVITW---QDAG-P-QIVLDQLRAQKVNVVTL----P---RV-----PA--TLEQM-YANIRQLAKTLQVPEQGDALVT---QINQRLERVQQ-----NVA---AKKAP--VKAMF 165
```

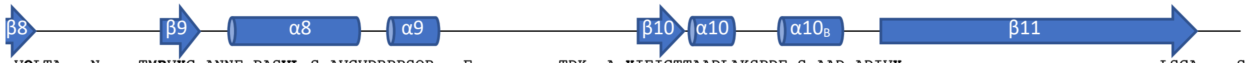


```
Mt-FecB   VQLTA---N----TMRVYG-ANNF-PASVL-S-AVGVDRPPSQR---F---------TDK--A-YIEIGTTAADLAKSPDF-S-AAD-ADIVY---------------------------LSCA----S 300
Mt-FecB2  LQGRL---W--QGNVVATL-AGWR--TDFL-N-DMGLVIADSIK---P--------FAVD--Q-RGVIP--------RDHIKA-VLDAADVLI---------------------------WMTE----- 244
Ms-FecB2  LGGTL---Y--RGGVQATP-PGWR--TDFL-T-QMGLTVLQV----------------------PALIP--------RDEIAS-VLDGADVLI---------------------------WTTE----- 257
Ec-FitE   IQANQ---G----KINVMH-SYHS-LGRVL-R-DAGFRFPPLIE--SI---------PEG--G-RMDVSA--------ERL-P-ELD-ADFVF---------------------------ATWR----G 249
Sp-PiaA   TMINAADTS----KFWIYT-SKDP-RANYL-T-DLGLVFPESL--KEF---------ESEDSF-AKEISAE-------EANKINDAD-VIITYGDDKTLEALQKDPLLGKINAIKNGAVAVIPD----N 308
Sa-SirA   VNFRA---D----HTRIYA--GGY-AGEIL-N-DLGFKRNKDLQ-KQV---------DNG-KD-IIQLTS-------KESI-P-LMN-ADHIF---------------------------VVKS----D 261
Sa-HtsA   AVVAK---A----GLLAHP-NYSY-VGQFL-N-ELGFKN--ALS---DDVTKGLSKYLKG--P-YLQLD--------TEHL-ADLNP-E-RMI---------------------------IMTD----H 266
Bc-YfiY   VRFMP---G----DVRIYH-GDTF-SGVIL-K-ELGFKRPGDQN---K---------D-D--F-AERNVS-------KERI-S-AMD-GDVLF---------------------------YFTF----D 198
Ba-FpuA   AQAFT---AKNVPTFRILT-DNSL-ALQVT-K-KLGLTNT-FEA---G---------KSEPDG-FKQTT--------VESL-Q-SVQ-DSNFI---------------------------YIVA----D 267
Bs-FhuD   IRIRQ---G----NIYIYP-EQVY-FNSTLYG-DLGLKAPNEVK---A---------A-K--A--QELIS-------LEKL-S-EMN-PDHIF---------------------------VQFS----D 237
Sp-FhuD   FEDFD---K----KIYAYG-KNWGRGSEVLYQ-AFGLQMPKALD-DAT---------KKE--G-WTEVPK--------EEV-G-KYA-GDVII---------------------------TAKA----K 225
Sa-FhuD2  FDEFD---K----KLYTYGDNWGR-GGEVL-YQAFGLKMQPEQQ-KLT---------AKA--G-WAEVKQ--------EEI-E-KYA-GDYIV---------------------------STSE----G 247
Bs-YclQ   IMAND---G----KISAFG-PKSR-YGLIH-D-VFGVAPA-DQN---I---------KAS--THGQSVS--------YEYI-S-KTN-PDYLF---------------------------VIDRGTAIG 241
Bs-FeuA   MQTNG---K----DIYVFG-KDFGRGGSIIYK-DLGLQATKLTKEKAI---------DQG-PG-YTSISL--------EKL-P-DFA-GDYIF---------------------------AGPW----Q 261
Sa-IsdE   LMGVP---G----SYLVAT-DKSY-IGDLV-K-IAGGENV--IK---V---------KDR--Q-YISSN--------TENL-L-NIN-PDIIL---------------------------RLPH--GMP 232
Cg-HmuT   LYARG-TGG----VFFILG-DAYG-GRDLI-E--GLGGVD-MAA---E--------KGIM--D-LAPA---------NAEA-LAELN-PDVFV---------------------------MMSEG---L 301
Sd-ShuT   ILSAGG--S----APQVAG-KGSV-ADAIL-S-LAGAEN--VAT---H-----------Q--Q-YKSY--------SAESL-I-AAN-PEVIV---------------------------VTSQ----- 222
```

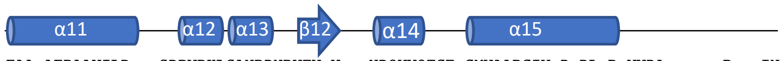


```
Mt-FecB   ------EAA-AERAAVILD---SDPWRKLSANRDNRVFV-V---NDQVWQTGE-GMVAARGIV-D-DL-R-WVDA------P---IN           359
Mt-FecB2  --------S-PEDEKALLA---DPEIAASQATAQRRHIF-TSKEQAGAIA-FS-SVLSYPVVA-EQLP-P-QISQILG---------           304
Ms-FecB2  --------S-DQDRDALLA---DPIVAQLAATRRDRNIF-TTKELAGAIA-FA-SPLSYPVVA-DQLP-P-ELARVLG---------           317
Ec-FitE   ----DTGGK-PQDELATMEKVMPGWCQFLTACRSGRYVL-I---SREEAI-SN-SFASLGLMA-AQI-QSQIAGRPL----P-----           312
Sp-PiaA   ------------------------------------------TPLAASCT--P-TPLSINYTI-EEYL-N-LLGNACKNAK------           341
Sa-SirA   PNAKDAALV-KKTESEWTS---SKEWKNLDAVKNNQVSDDL---DEITWNLAG-GYKSSLKLI-D-DL-YEKLNIEKQ---------           328
Sa-HtsA   ------AKKDSAEFKKLQE---DATWKKLNAVKNNRVDI-V---DRDVWARSR-GLISSEEMA-K-EL-VELSKK------------           324
Bc-YfiY   --KGNEKKG-SELEKEYIN---DPLFKNLNAVKNGKAYK-V---DDVIWNTAG-GVIAANLLL-D-DIEK-RFV-------------           281
Ba-FpuA   ------E---DNIFDTQLK--GNPAWEELKFKKENKMYK-L---KGDTWIF-G-GPESATSLA-T-QV-ADVMTA------------           322
Bs-FhuD   ---DENADK-PDALKDLEK---NPIWKSLKAVKEDHVYV-N-SVDPLA--QGG-TAWSKVRFL-K-AA-AEKLTQ-NKLA-------           301
Sp-FhuD   ------D---AAQP-EFQK---TAMWQNLEAVQNKYAFN-V---DSSVYW-YN-DPYTLDVIR-K-DL-KKQLLA-LPT--------           281
Sa-FhuD2  ------K-P-TPG--YEST---NM-WKNLKATKEGHIVK-V---DAGT-YWYN-DPYTLDFMR-K-DL-KEKLIKAAK---------           302
Bs-YclQ   ------ETS---STKQVVE---NDYVKNVNAVKNGHVIY-L---DSATWYLSGGGLESMTQMI-K-EV-KDGLEKEN----------           299
Bs-FeuA   ------SG---GDDGGVFE---SSIWKNLNAVKNGHVYK-M---DPIGFYFT-DPISLEGQLE-F-IT--ESLTKL-----------           316
Sa-IsdE   ------EEV-KKMFQKEFK--QNDIWKHFKAVKNNHVYD-L---EEVPFGITA-NVD-ADKAM-T-QL-YDLFYK------------           289
Cg-HmuT   ------V-S-TGGIDGLME---RPGIAQTTAGQNQRVLA-L---PDGQSL-AF-GAQ-TGELLLR-AS-R-ELYV-Q----------           356
Sd-ShuT   -------MV-DGD-INRLR---SIAGITHTAAWKNQRIITV---DQNLIL-GM-GPR-IADVV-E-SLHQQLWP-------------           277
```
