# Supplementary figures and images for: Differentiating the roles of Mycobacterium tuberculosis substrate binding proteins, FecB and FecB2, in iron uptake
Source: PLoS Pathog. 2023 Sep 25;19(9):e1011650. doi: 10.1371/journal.ppat.1011650 (PMC10553834; doi:10.1371/journal.ppat.1011650)

A

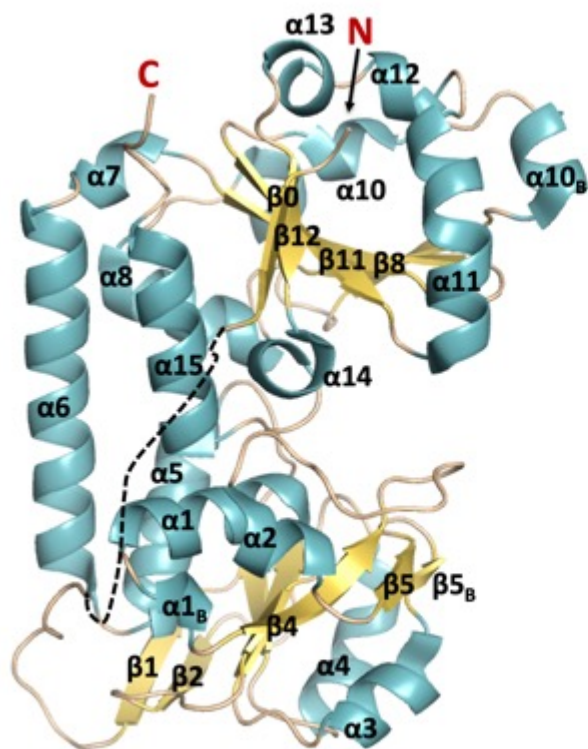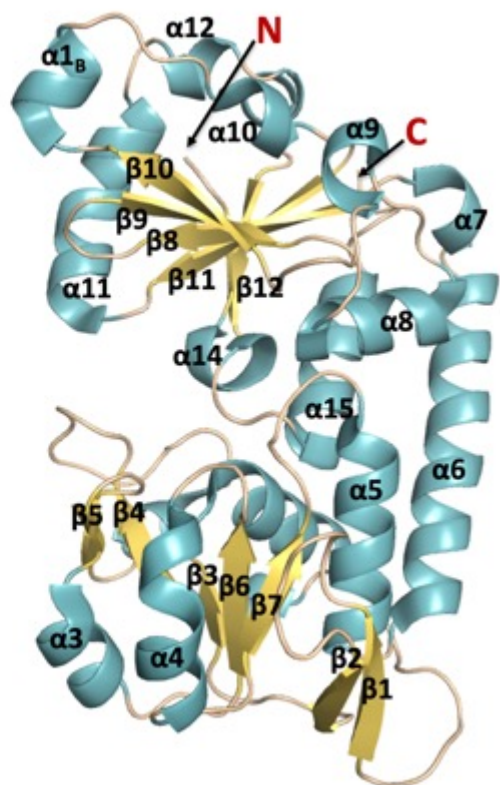

B

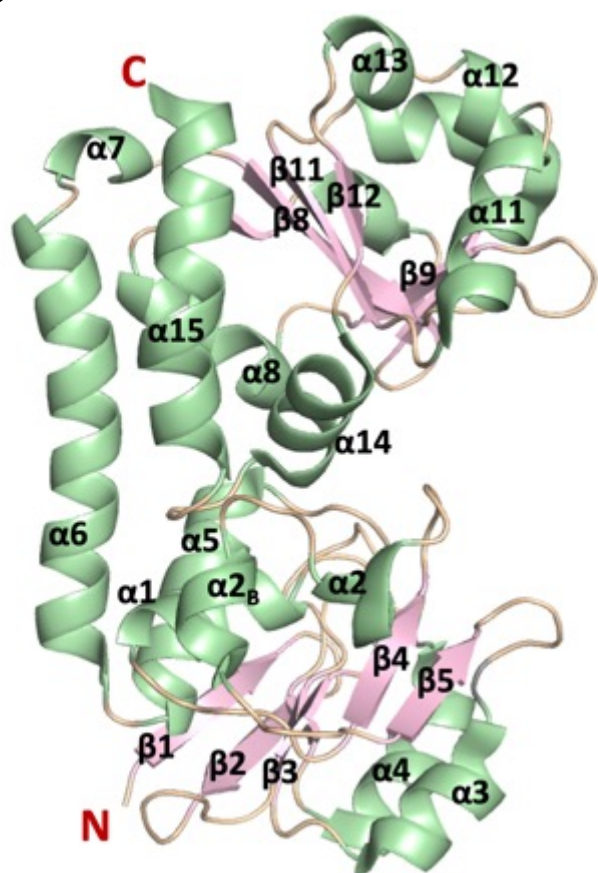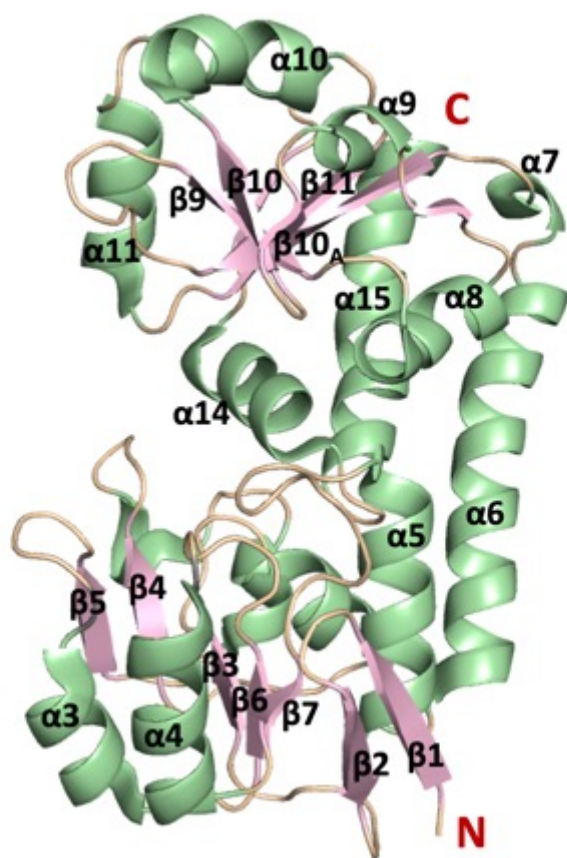

Supplement: S2 Fig — FecB (A) and FecB2 (B) are colored by secondary structure where α-helices and β-strands, respectively, are colored blue and yellow for FecB and green and pink for FecB2. N- and C-termini are labeled in red. FecB and FecB2 are shown with two identical 180° orientations. (PDF) [file ppat.1011650.s002.pdf]

**A**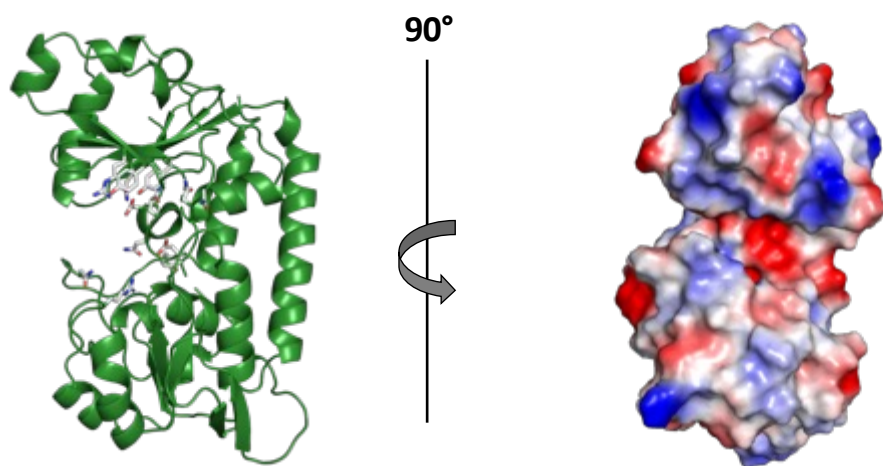**B**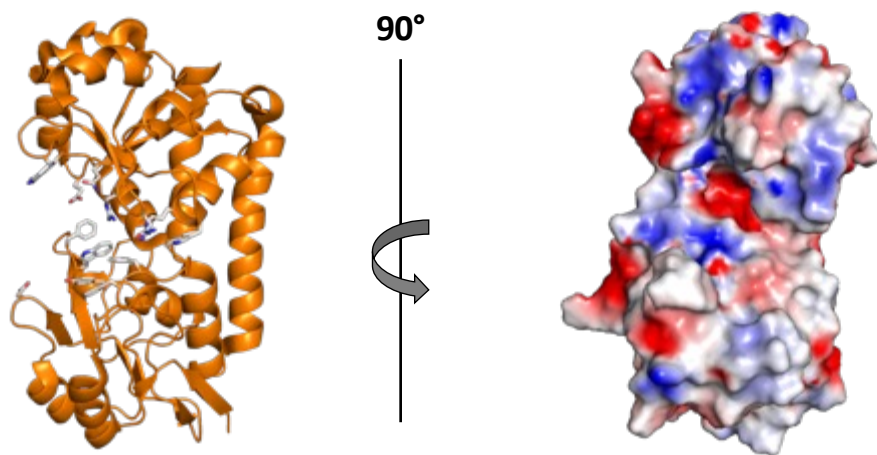**C**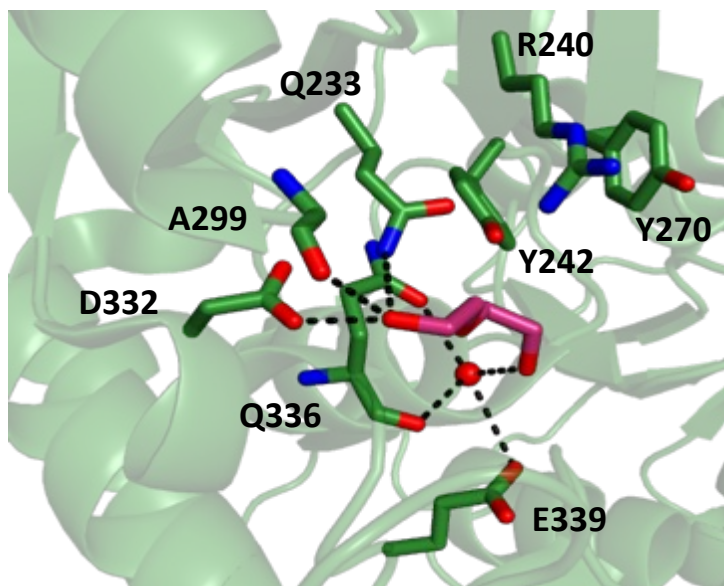

Supplement: S3 Fig — (A and B) Left panels: (A) FecB (green) and (B) FecB2 (orange) are shown as cartoon depictions and potential ligand binding residues shown as white sticks. Right panels: APBS (Adaptive Poisson-Boltzmann Solver ‐ a PyMol plugin) generated electrostatic surfaces of the ligand binding sites of FecB and FecB2. With negatively and positively charged molecular surfaces colored in red and blue, respectively. (C) FecB crystallized with a PEG molecule (pink stick) observed in the ligand-binding pocket. Polar interactions within 4 Å between FecB residues and PEG are indicated with dashed black lines, coordinating residues are shown as green sticks, and a coordinating water molecule is shown as a red sphere. Arg240, Tyr242 and Tyr270 are shown for comparison to Fig 3. (PDF) [file ppat.1011650.s003.pdf]

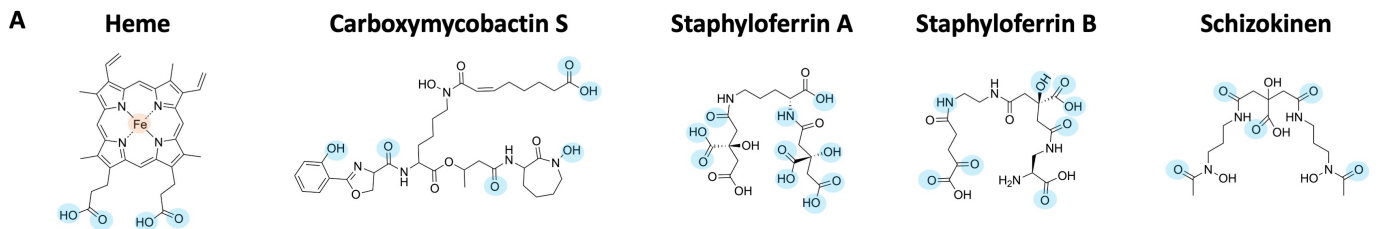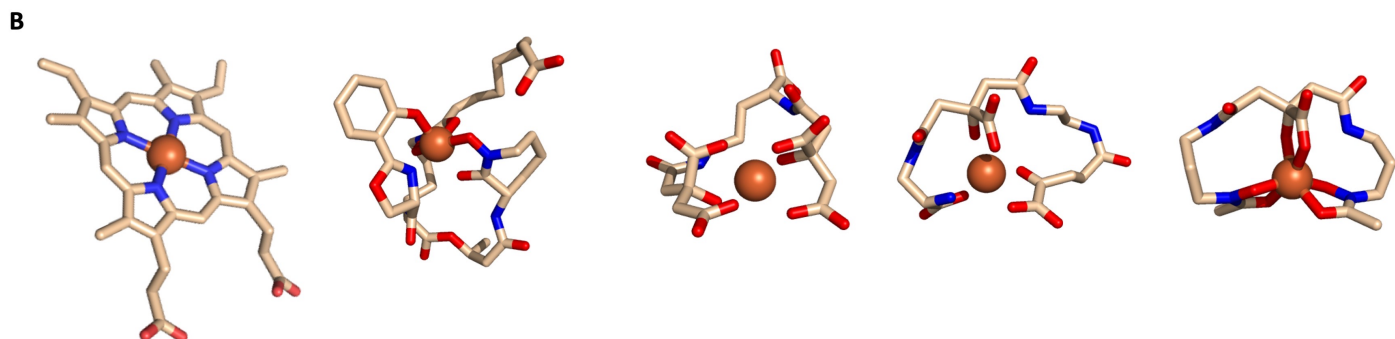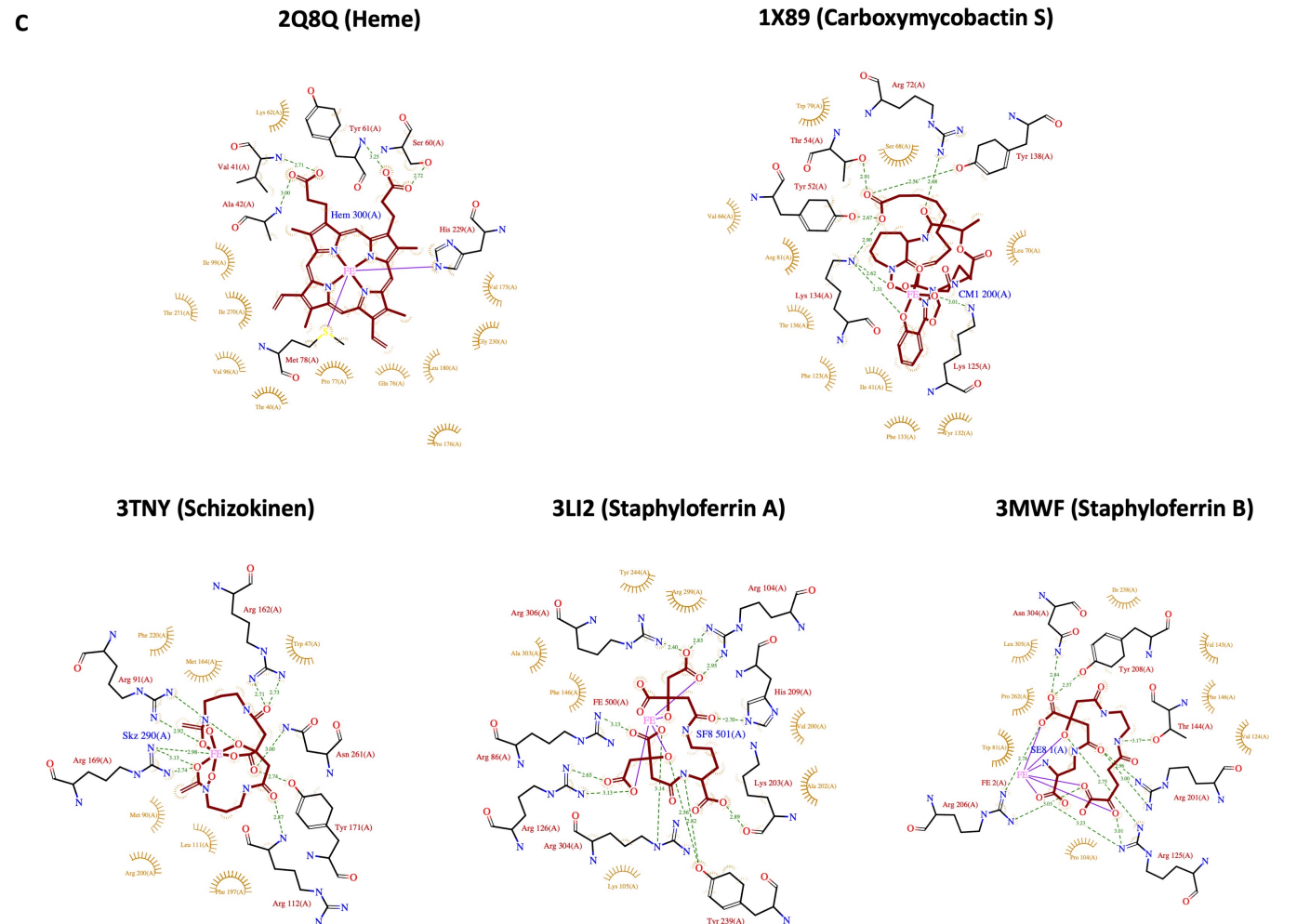

Supplement: S4 Fig — A and B represent molecular structures of heme and the apo- and ferric-siderophore respectively, and C represents heme bound to Sa-IsdE (PDB 2Q8Q) and ferric-siderophores bound to PBPs as in staphlyoferrin A bound to Sa-HtsA (PDB, 3LI2), staphyloferrin B bound to Sa-SirA (PDB, 3MWF) and schizokinen bound to Bc-YfiY (PDB, 3TNY) and carboxymycobactin S bound to siderocalin (PDB, 1X89). Depcitions were produced in LigPlot. (PDF) [file ppat.1011650.s004.pdf]

**A****FecB****+HisTag**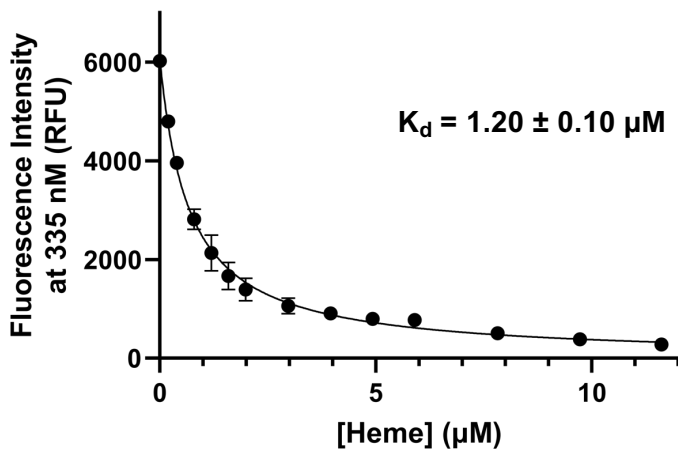**FecB2**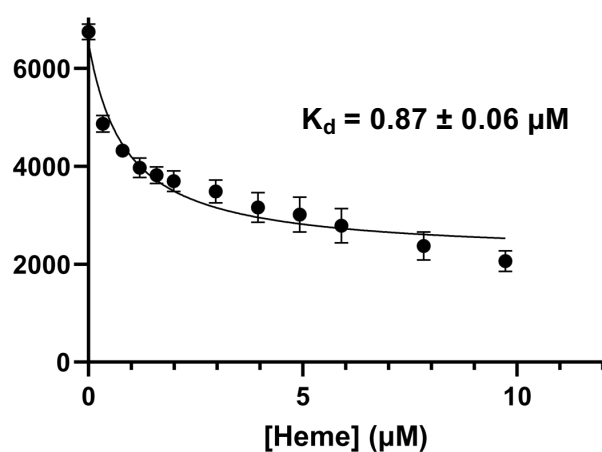**B****No-HisTag**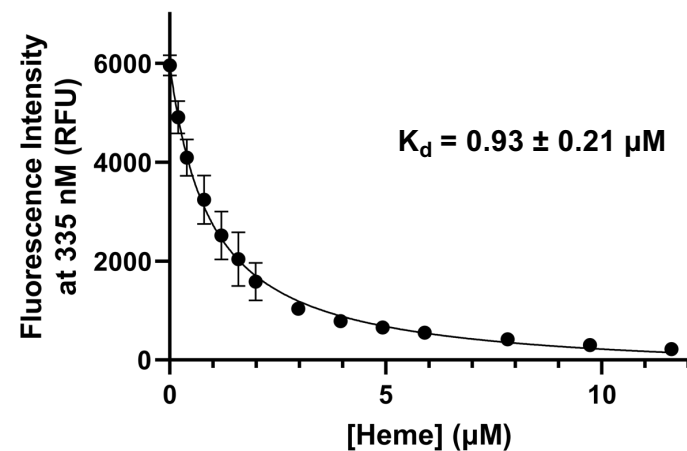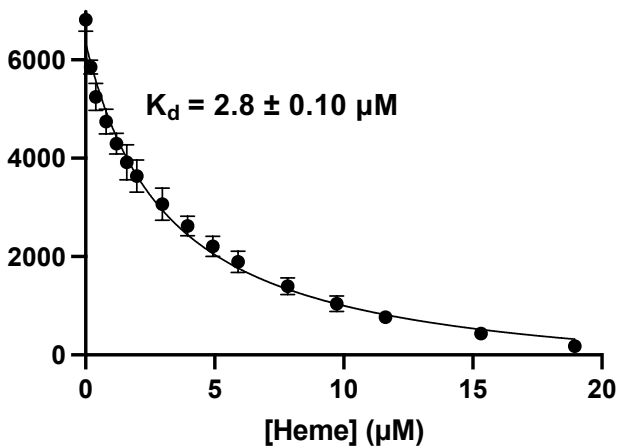

Supplement: S5 Fig — Representative fluorescent emission intensities at 335 nm after excitation at 280 nm of 100 nM FecB (left panels) and 100 nM FecB2 (right panels) with increasing concentrations of heme. Experiments were performed with (A) and without (B) the HisTag. Curves were fit using the equation in the methods and heme affinities (Kd) are included for each titration. (PDF) [file ppat.1011650.s005.pdf]

# A +HisTag FecB

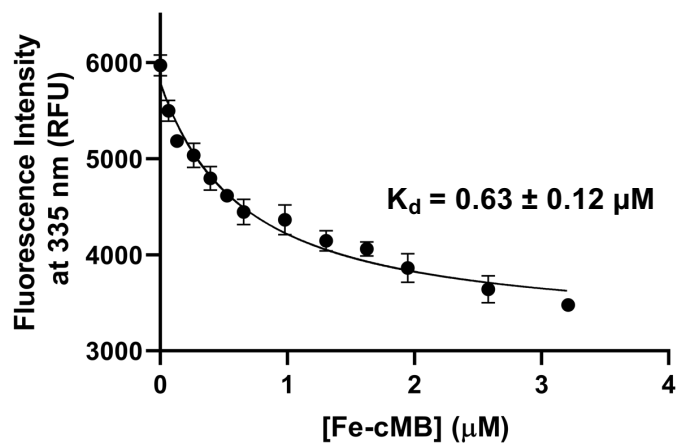

# FecB2

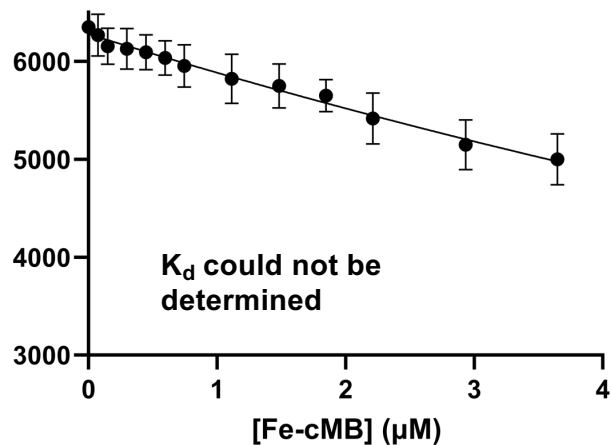

# B No-HisTag

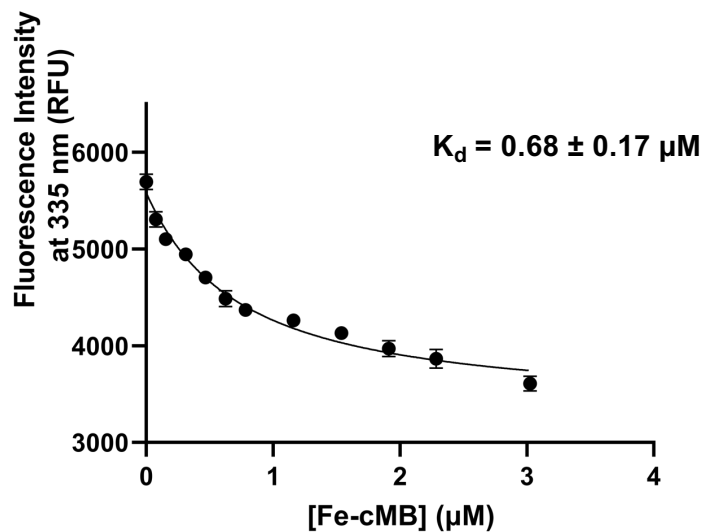

Supplement: S6 Fig — Representative fluorescent emission intensities at 335 nm after excitation at 280 nm of 100 nM FecB (left panels) and 100 nM FecB2 (right panels) with increasing concentrations of Fe-cMB. Experiments were performed with (A) and without (B) a HisTag. Curves were fit using the equation in the methods and Fe-cMB affinities (Kd) are included for each titration. Notably, no titration was carried out for FecB2 without a Histag, as Fe-MB bound so poorly to FecB-HisTag. (PDF) [file ppat.1011650.s006.pdf]

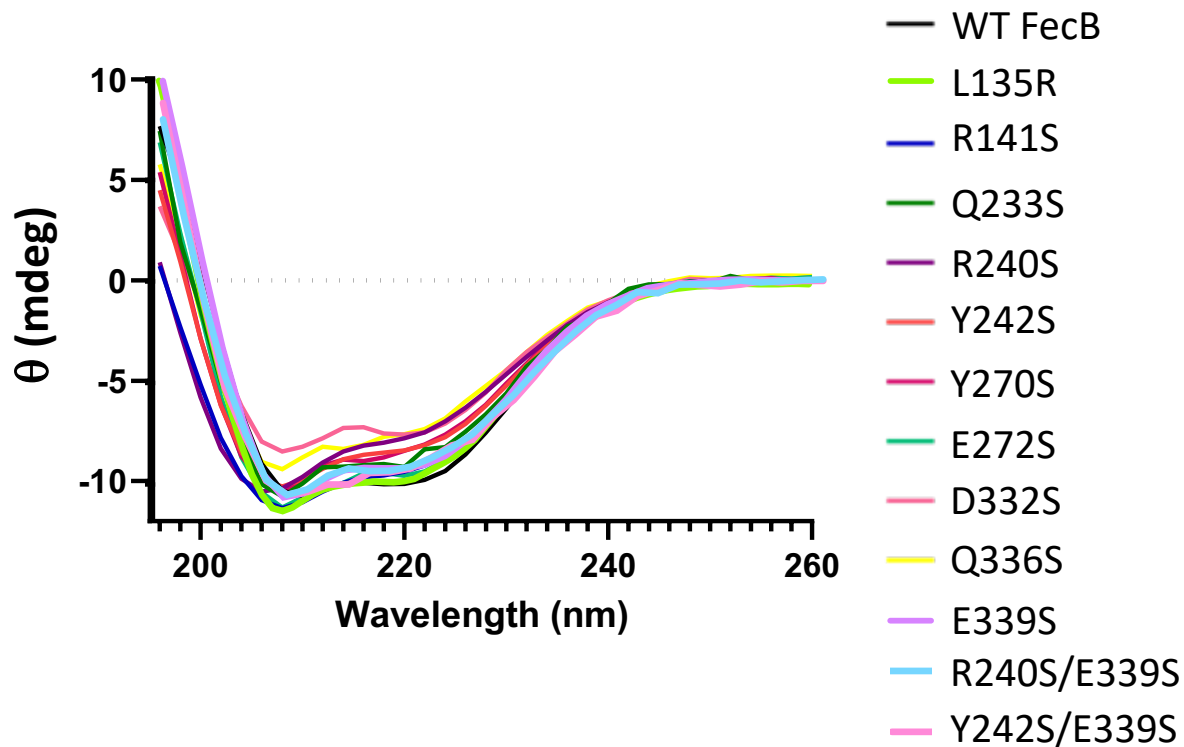

Supplement: S7 Fig — CD was performed to ensure that no major structural changes occurred with the generation of FecB variants compared to wild-type (WT) FecB. Experiments were performed at 25°C using a Jasco J-810 spectropolarimeter. WT and variant FecB samples (5 μM) were analyzed in 5 mM Tris pH 7.4, 35 mM NaCl, 1% glycerol. (PDF) [file ppat.1011650.s007.pdf]

# A

## Native FecB+His

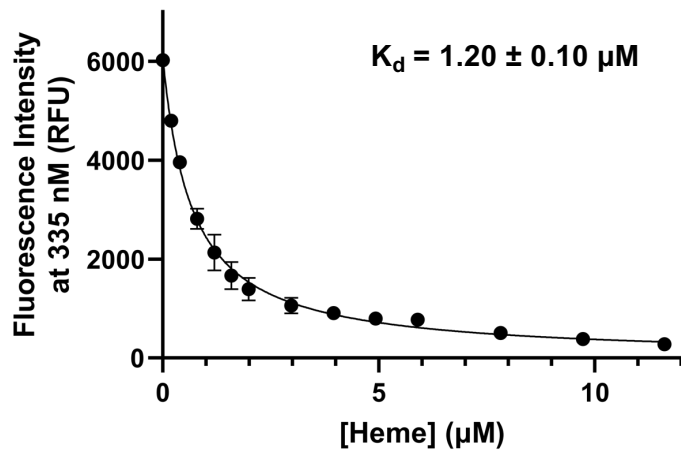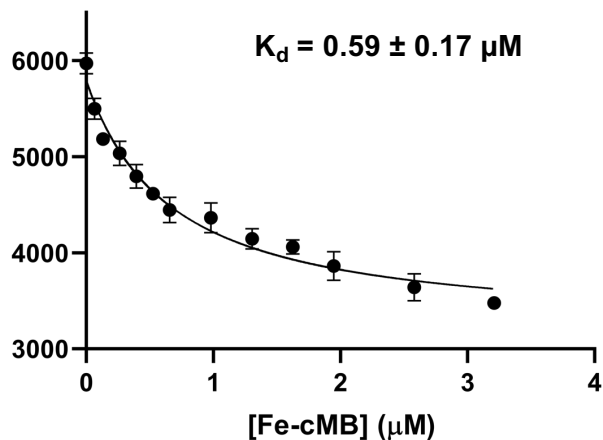

# B

## Refolded FecB +His

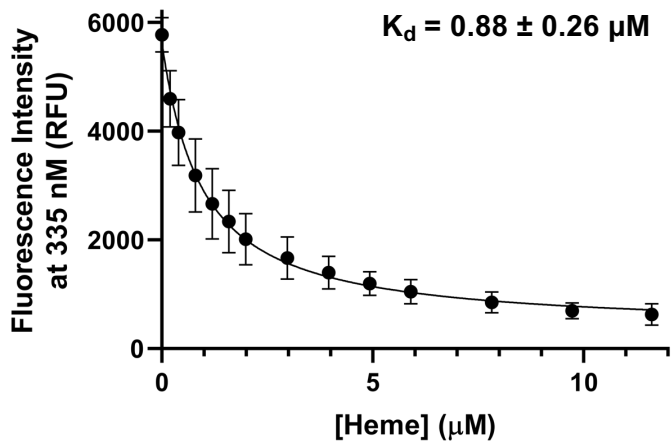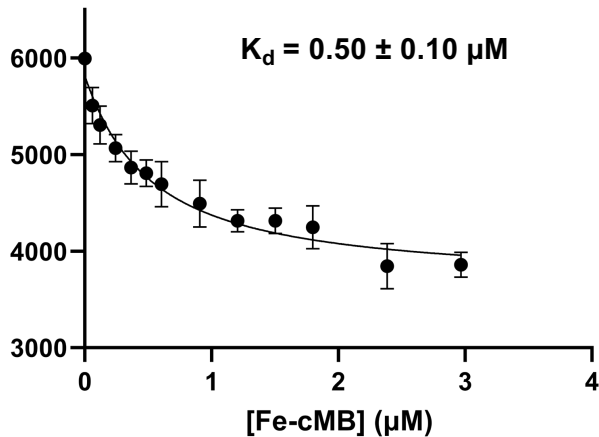

Supplement: S8 Fig — Representative fluorescent emission intensities at 335 nm after excitation at 280 nm of WT-FecB with increasing concentrations of heme. Titrations were performed with WT-FecB (100 nM) purified in its (A) native, soluble form and (B) refolded form. Curves were fit using the equation in the methods and heme or Fe-cMB affinities (Kd) are included for each titration. (PDF) [file ppat.1011650.s008.pdf]

## A. Apo-cMB

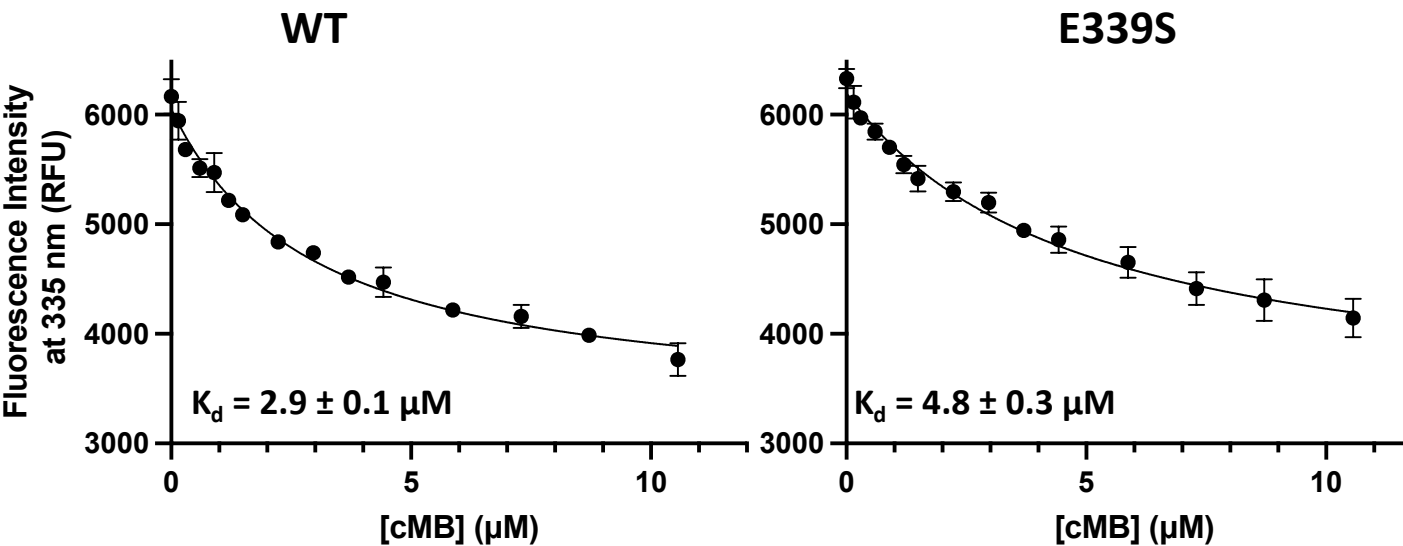

## B.

### Fe-mycobactin J (Fe-MBJ)

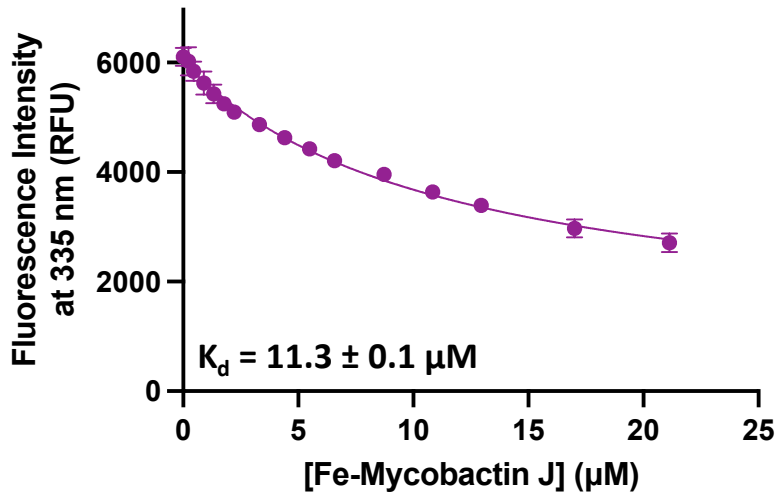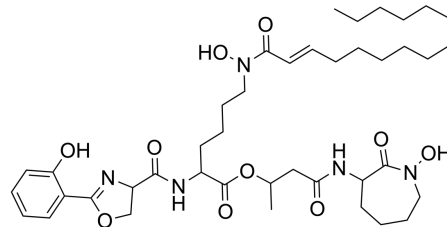

**C.**

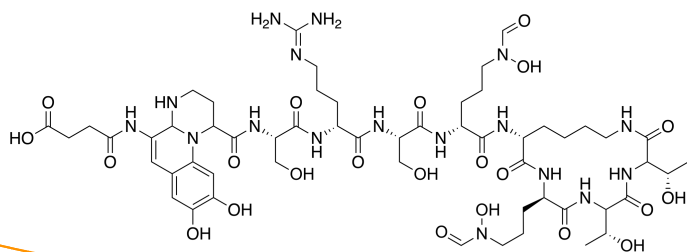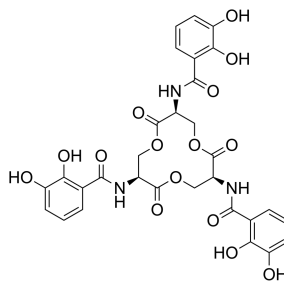

Supplement: S9 Fig — Representative fluorescent emission intensities at 335 nm after excitation at 280 nm of FecB with increasing concentrations of apo-cMB or Fe-siderophores. (A) Apo-cMB affinity to WT- and E339S-FecB variant tested. (B) Affinity of ferric-MBJ for FecB. (C) Affinity of Fe-pyoverdines (Fe-pyo) and Fe-enterobactin (Fe-EB) for FecB. Curves were fit using the equation in the methods and affinities (Kd) are included for each titration where possible. (PDF) [file ppat.1011650.s009.pdf]

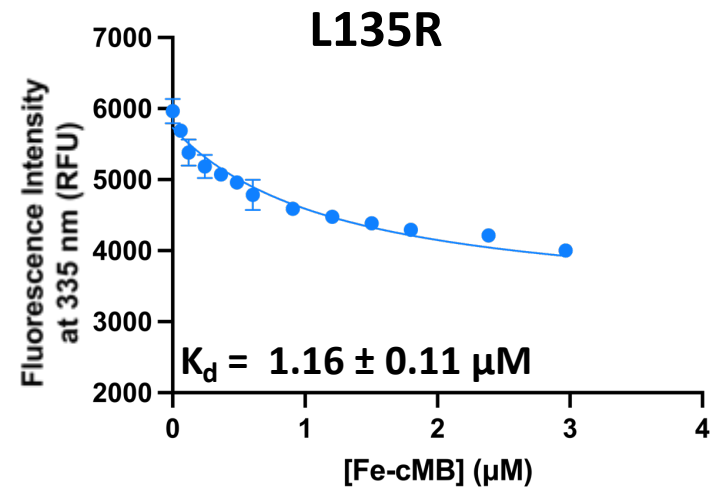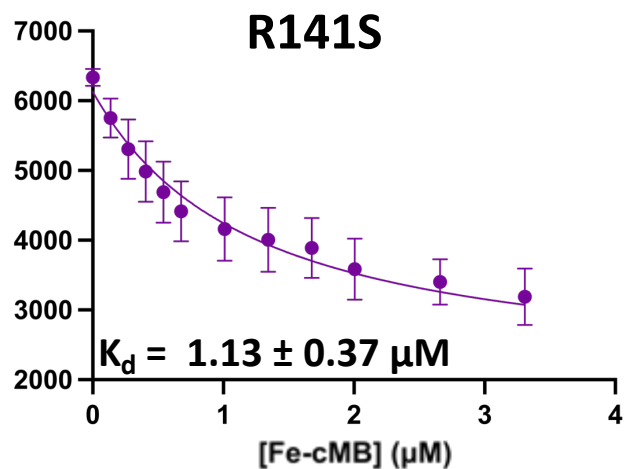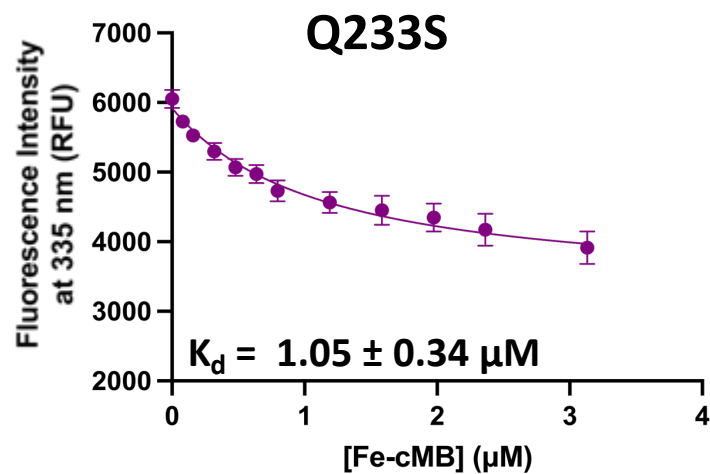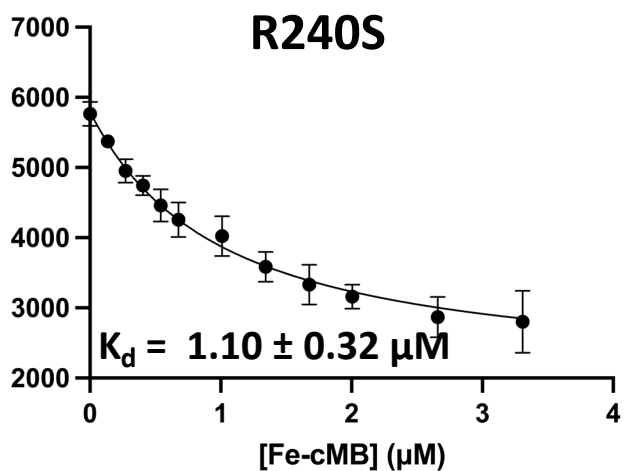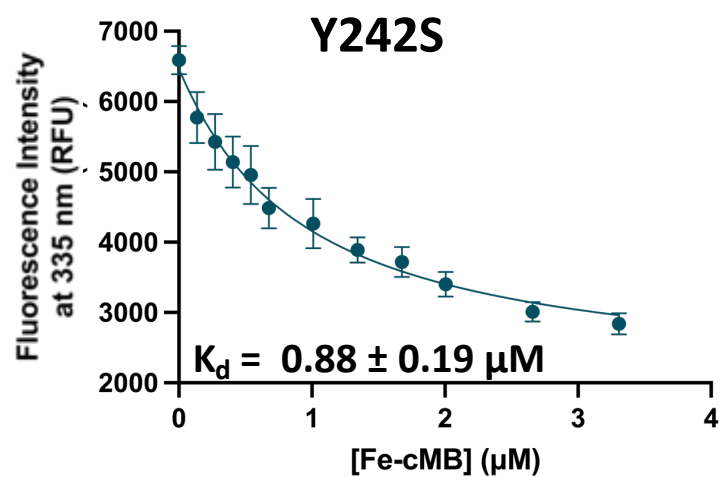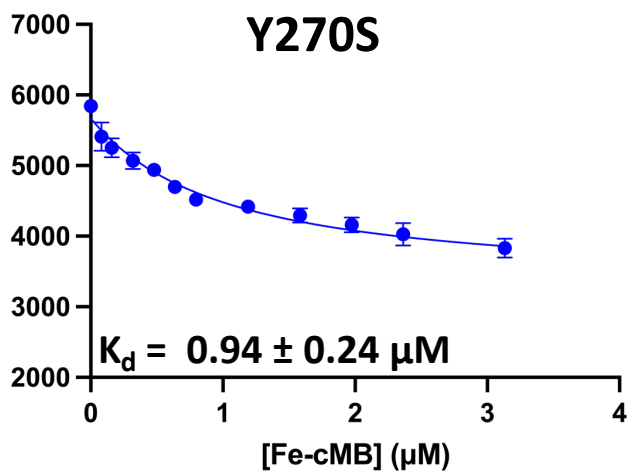

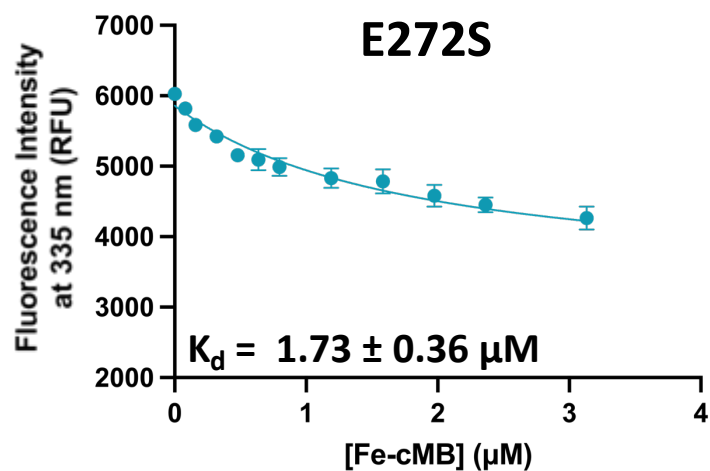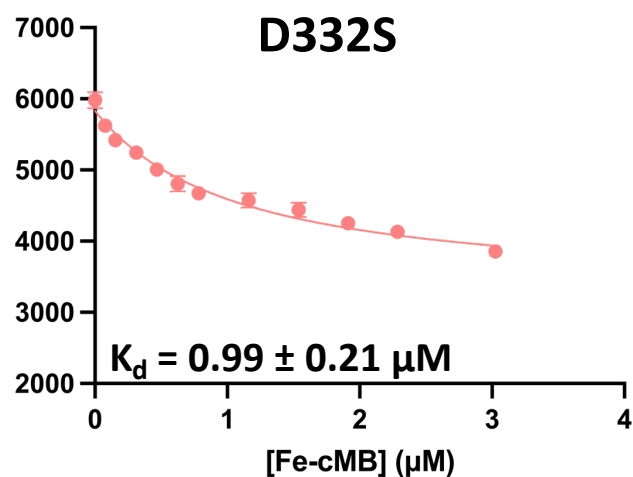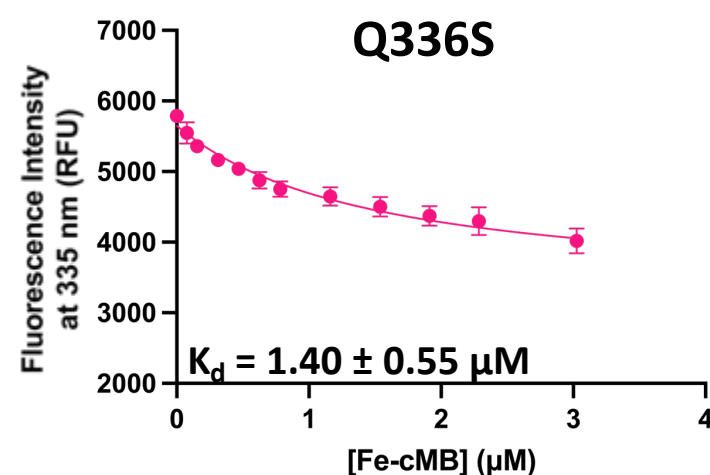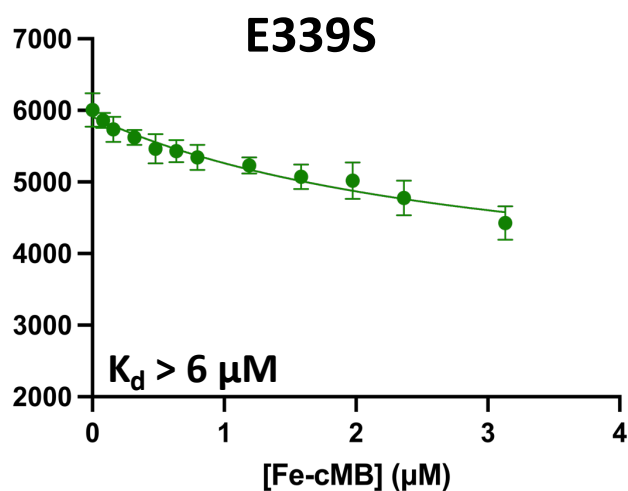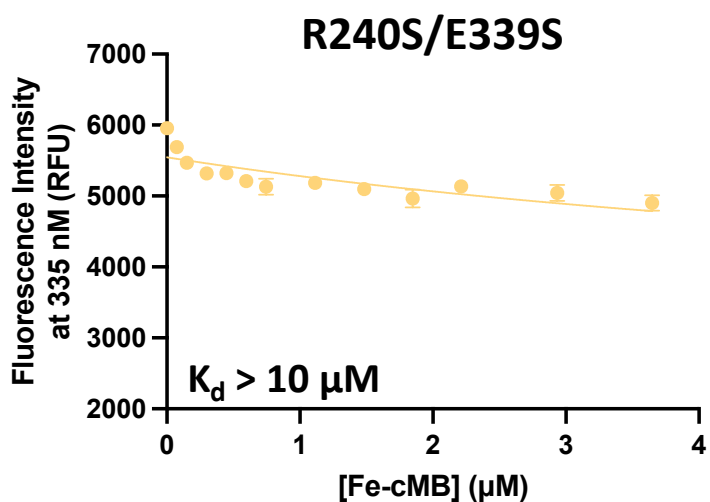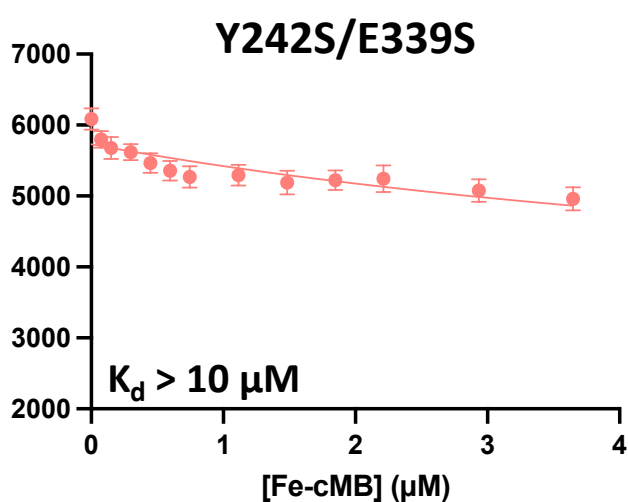

Supplement: S10 Fig — Representative fluorescent emission intensities at 335 nm after excitation at 280 nm of FecB variants with increasing concentrations of Fe-cMB. FecB variants tested for Fe-cMB affinity include FecB L135R, R141S, Q233S, R240S, Y242S, Y270S, E272S, D332S, Q336S, and E339S mutations, along with the double mutants FecB R240S-E339S and Y242S-E339S. Curves were fit using the equation in the methods and Fe-cMB affinities (Kd) are included for each titration. (PDF) [file ppat.1011650.s010.pdf]

## FecB2

WT

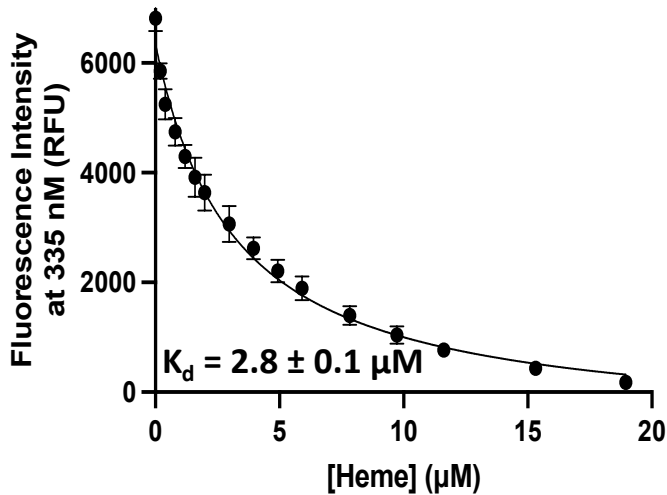

Y39S

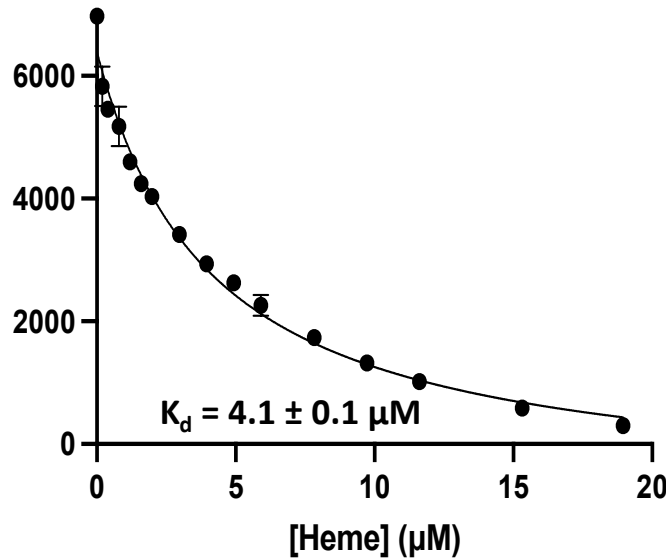

R184S

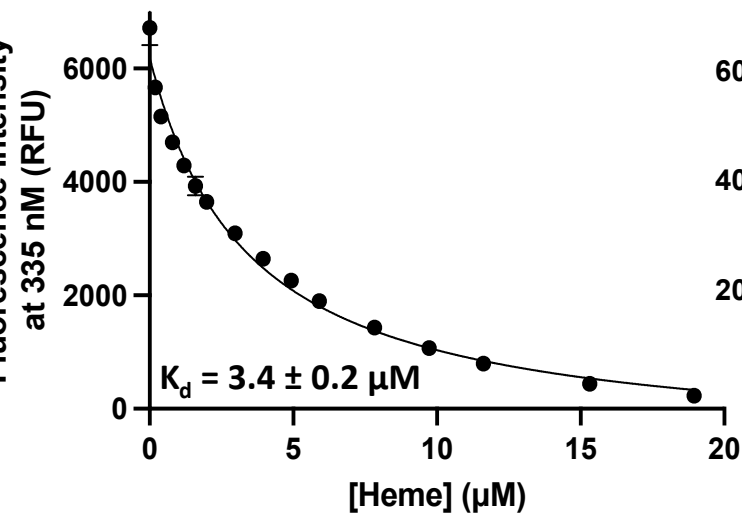

W58S

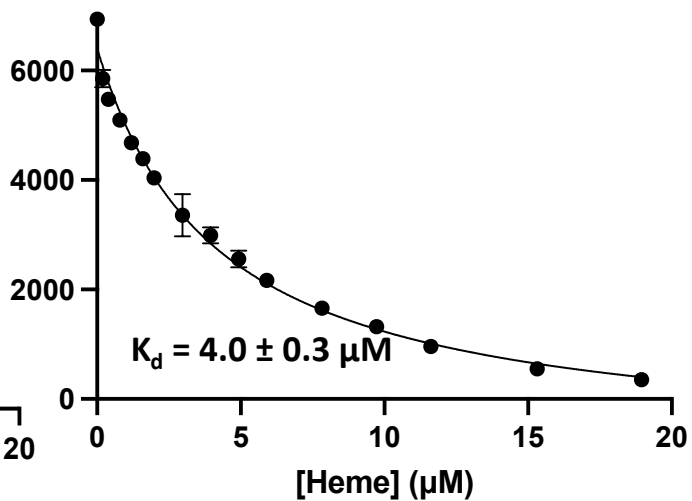

Supplement: S11 Fig — Representative fluorescent emission intensities at 335 nm after excitation at 280 nm of FecB2 variants with increasing concentrations of heme. FecB2 variants tested for heme affinity include FecB L135R, R141S, Q233S, R240S, Y242S, Y270S, E272S, D332S, Q336S, and E339S mutations, along with the double mutants R240S-E339S and Y242S-E339S. Curves were fit using the equation in the methods and Fe-cMB affinities (Kd) are included for each titration. (PDF) [file ppat.1011650.s011.pdf]

# A

## IP:FLAG (IP-<sup>FL</sup>)

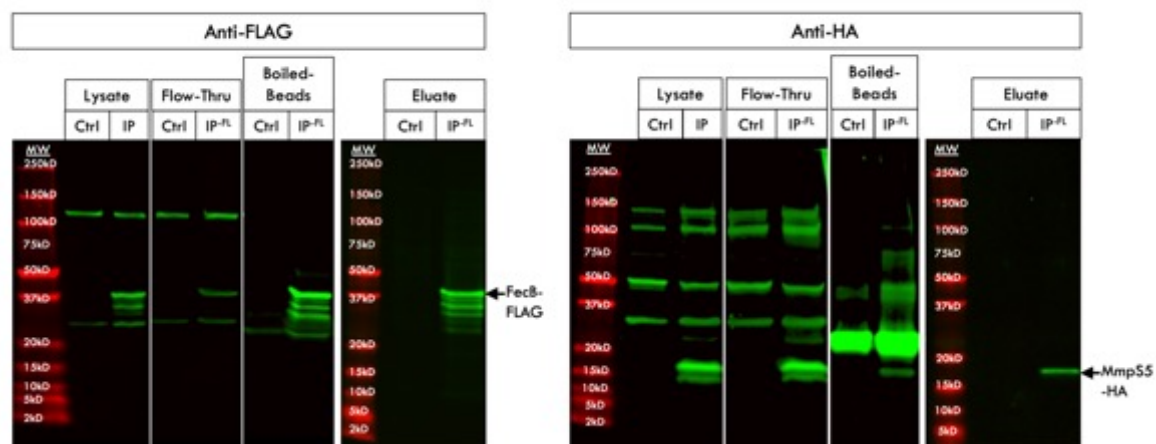

# B

## IP:HA (IP-<sup>HA</sup>)

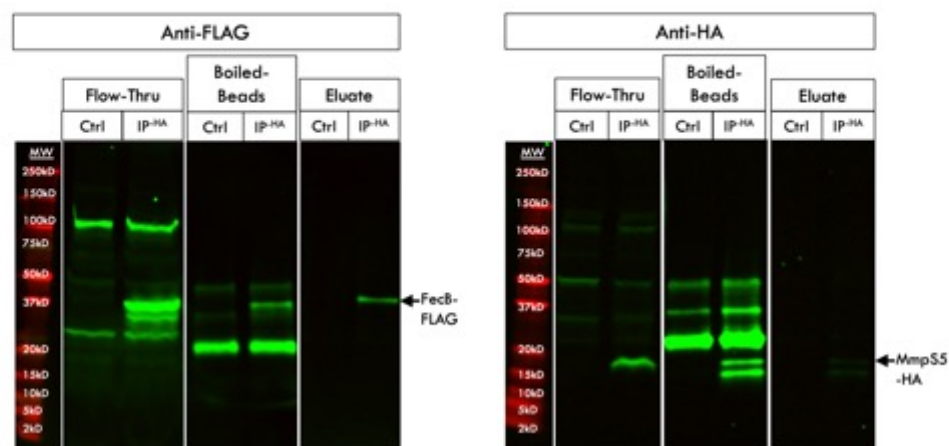

# C

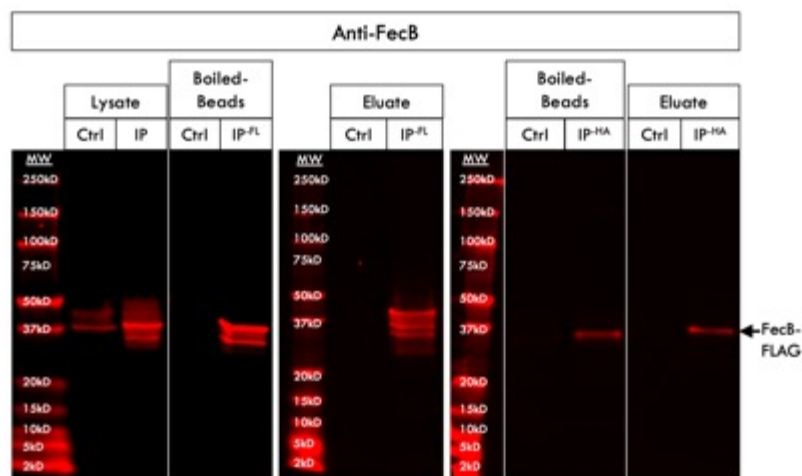

Supplement: S13 Fig — Western Blot analysis of protein co-IPs with MtbΔfecB with vectors expressing FecB-FLAG and MmpS5-HA, and the negative control whereby Mtb contains vectors that express the tags alone. Western Blot analysis of both the boiled beads and eluate of the (A) co-IP using the anti-FLAG beads to pull-down FecB-FLAG, probed with anti-FLAG (left panel) and anti-HA (right panel) antibody that recognizes FecB-FLAG andMmpS5-HA (respectively), (B) co-IP using the anti-HA beads to pull down MmpS5-HA, probed with an anti-FLAG (left panel) and anti-HA (right panel) antibody that recognizes FecB-FLAG and MmpS5-HA (respectively), and (C) co-IP using the anti-FLAG (left panel) and anti-HA beads (right panel) to pull down FecB-FLAG and MmpS5-HA (respectively), probed with anti-FecB antiserum that recognizes FecB. This figure shows all steps that generated Fig 6, so includes all lysates, washes, boiled beads and eluents. (PDF) [file ppat.1011650.s013.pdf]

## Fe-cMB

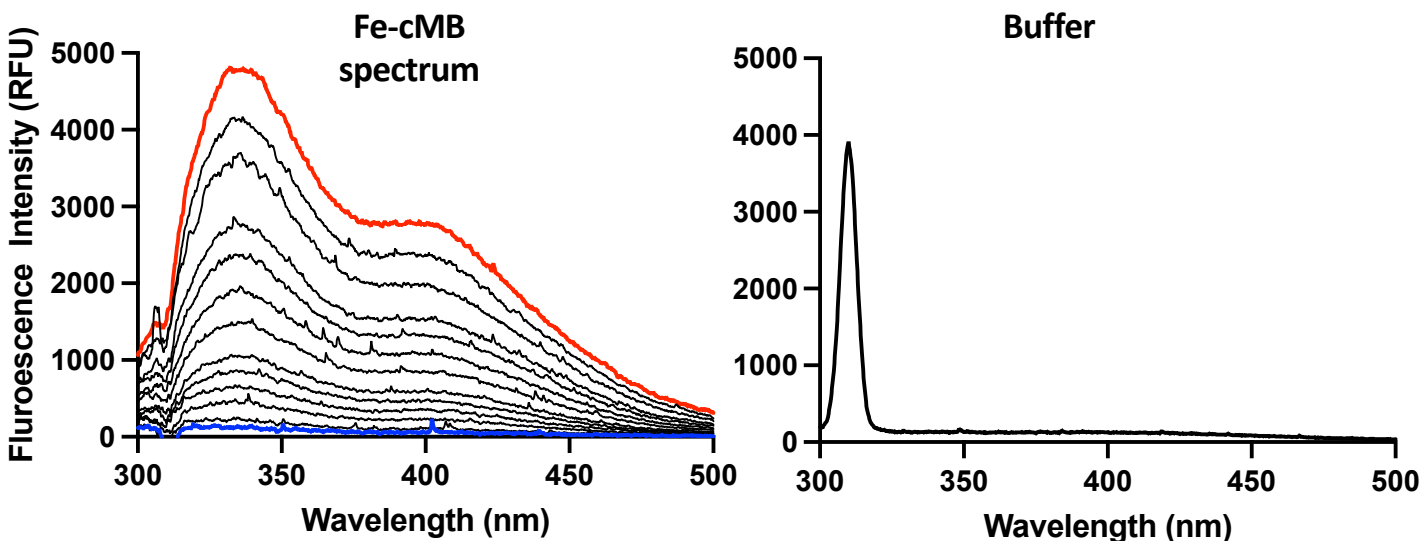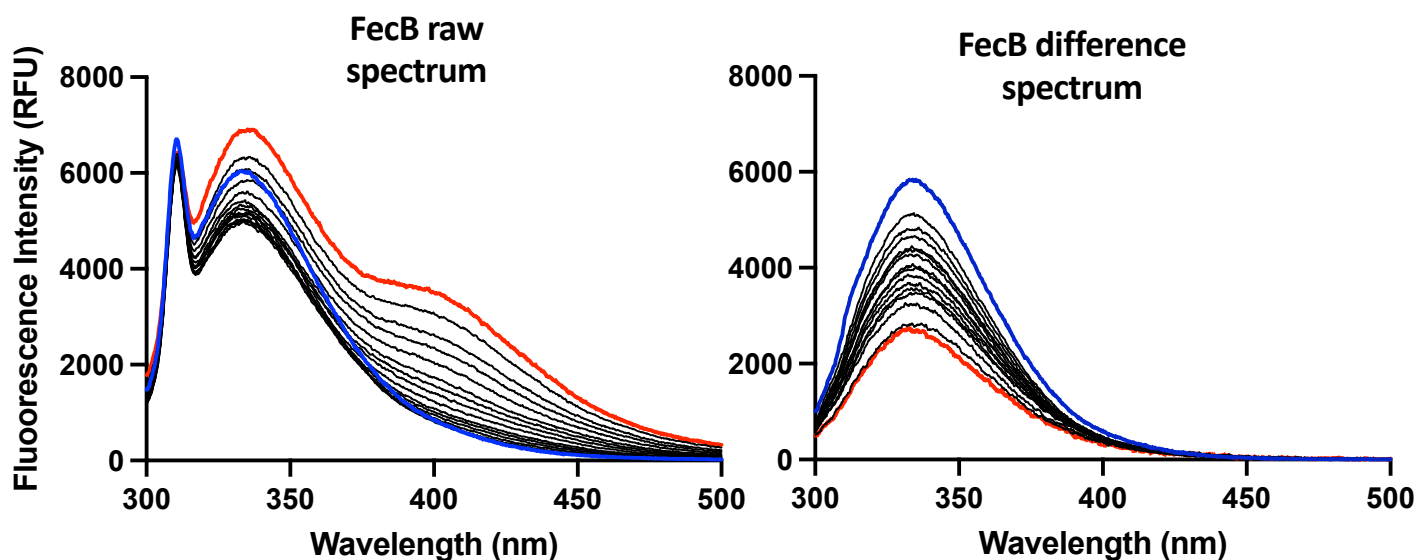

## Heme

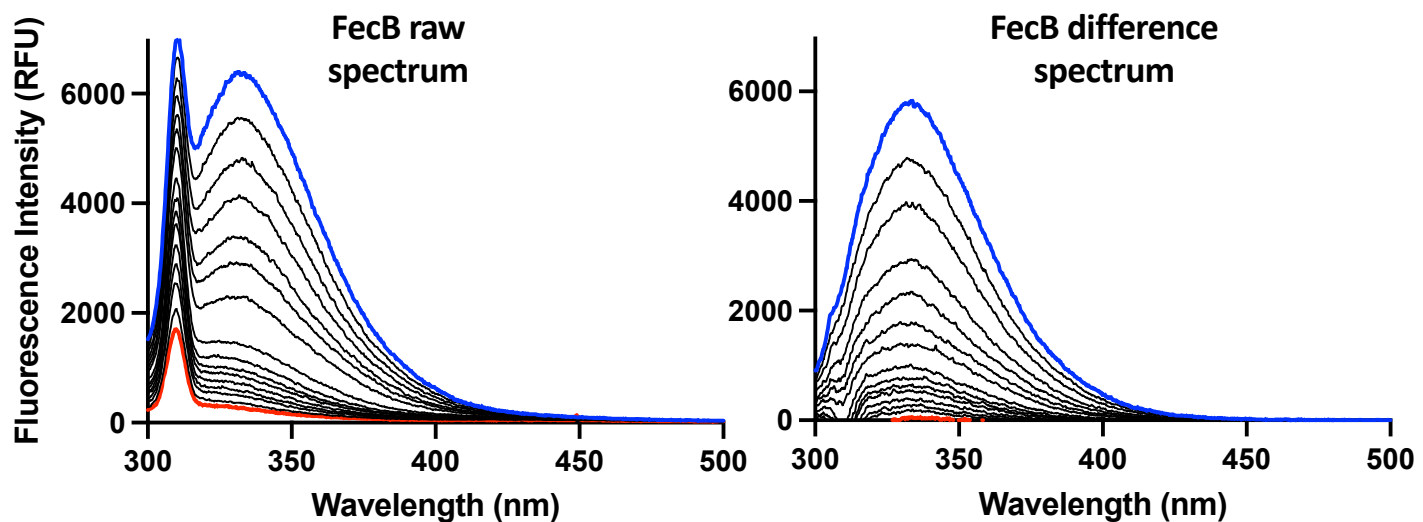

Supplement: S14 Fig — Representative titration experiments for FecB with ferric-carboxymycobactin (Fe-cMB) and heme. Raw data includes buffer fluorescence alone, and matched titrations of ligand into buffer (Fe-cMB spectrum) or into FecB (FecB raw spectrum). To attain the FecB difference spectrum, one must measure the three forms of raw data and subtract buffer and ligand signal from the protein-ligand experiment. The difference spectrum indicates fluorescence changes due to interactions between ligand and protein, as noise from ligand alone and buffer has been subtracted. The data from difference spectra is then plotted and fit to determine the affinity of the protein for its ligand. (PDF) [file ppat.1011650.s014.pdf]
