# Supplementary material for: Differentiating the roles of Mycobacterium tuberculosis substrate binding proteins, FecB and FecB2, in iron uptake
Source: PLoS Pathog. 2023 Sep 25;19(9):e1011650. doi: 10.1371/journal.ppat.1011650 (PMC10553834; doi:10.1371/journal.ppat.1011650)
Supplement: S12 Fig — (A) Peptides that were identified by MS (highlighted in yellow) results in 87% coverage of full-length FecB. The remaining residues not identified by MS are the first 42 residues, suggesting that FecB is processed and translocated to the periplasmic and cell-wall environments. (B) In the case of FecB2-FLAG, there is 99% coverage of the full-length FecB2 sequence, suggesting that the signal peptide is not processed. (PDF) [file ppat.1011650.s012.pdf]

Supplemental Figure 12A

All peptides from FLAG pulldown and sequencing are below. Those that are highlighted in yellow are included in the sequence of Msm-FecB (MSMEG_1039) and the coverage of these peptides is depicted in pink. The first 42 residues are not observed suggesting that the signal peptide has been processed.

AADPDLILGSAALTPGAFGELSDIAPTVFTGTPGAAWR;
AAPDVAAIR;
ADAVLKGDAWR;
ADAVLKGDAWRK;
AGAADDLIAGFEDAAR;
AGAADDLIAGFEDAARK;
AVGAATGR;DKADAVLK;
DKADAVLKGDAWR;
DTLRAVGAATGR;
FTDKPYEEIPADSDDFSIADADIVYVSFASPAAR;
IADAGVLGNQR
IVAAALPDGSDAQPSYLGTVVHDVPPAGDR;
VVLSGDQLDALCALGLQSR;
KLSANRDNR;
KTGEDNDAAHFQASVVQLTENTVR;
LSANRDNR;
NATADGADIAETTEVAGDPQR;
PDESCAPEPVAADPAPR;
RPDESCAPEPVAADPAPR;
RPDESCAPEPVAADPAPRR;
TGEDNDAAHFQASVVQLTENTVR;
VFGADNFPGDVLAAVGLDR;
VFGADNFPGDVLAAVGLDRPAAQR;
VFVVNNEVWQTGQNIVAAR;
VRNATADGADIAETTEVAGDPQR;
WVNAPIN

MSEMG_1039 (FecB)

We don’t see the first 42 residues

MLTFRPLVGTALIAAAASLISGCGTSGEQPVTQPSMTTSVTKIADAGVLGNQRRPDESCA
PEPVAADPAPRRVRNATADGADIAETTEVAGDPQRIVVLSGDQLDALCALGLQSRIVAAA
LPDGSDAQPSYLGTVVHDVPPAGDRAAPDVAAIRAADPDLILGSAALTPGAFGELSDIAP
TVFTGTPGAAWRDTLRAVGAATGRAGAADDLIAGFEDAARKTGEDNDAAHFQASVVQLTE
NTVRVFGADNFPGDVLAAVGLDRPAAQRFTDKPYEEIPADSDDFSIADADIVYVSFASPA
ARDKADAVLKGDAWRKLSANRDNRVFVVNNEVWQTGQNIVAARGILDDLRWVNAPIN

Supplemental Figure 12B

FecB2 (MSMEG_0438) peptides coverage. 95% including the apparent signal peptide.

RGFLAVTAGLALVAACSTDKPGEVADDGSVTVR;
GFLAVTAGLALVAACSTDKPGEVADDGSVTVR;
PGEVADDGSVTVR;
HAFGDTTIPGPPQR;
VVSAGLTEQDDLLAVGVVPIAVTDWFGGEPFGVWPWAQR
QLAGAQPAVLNLDNGIPVEEIAALKPDLIVATNAGLDADTYAK;
LSEIAPTVAQTGSEAFFEPWK;
LSEIAPTVAQTGSEAFFEPWKDQATIIGQAVFK;
DQATIIGQAVFK;
NAEMTELIK;
NAEMTELIKSVDDR;
NAEMTELIKSVDDRFTTVK;
SVDDRFTTVK;
TDHPQFSGKK
TDHPQFSGKKALLLGGTLYR;
SVDDRFTTVKTDHPQFSGK;
FTTVKTDHPQFSGK;
FTTVKTDHPQFSGKK
TDFLTQMGLTVLQVPALIPR;
TDHPQFSGK;
KALLLGGTLYR;
ALLLGGTLYR;
ALLLGGTLYRGGVQATPPGWR;
GGVQATPPGWR;
DEIASVLDGADVLIWTTESDQDR;
DEIASVLDGADVLIWTTESDQDRDALLADPIVAQLAATR;
DEIASVLDGADVLIWTTESDQDRDALLADPIVAQLAATRR;
DALLADPIVAQLAATR;
DALLADPIVAQLAATRR;
DRNIFTTK;
ELAGAIAFASPLSYPVVADQLPPELAR;

MSMEG_0438
MPRPLTRRGFLAVTAGLALVAACSTDKPGEVADDGSVTVRHAFGDTTIPGPPQRVVSAGLTEQDDLLA
VGVVPIAVTDWFGGEPFGVWPWAQRQLAGAQPAVLNLDNGIPVEEIAALKPDLIVATNAGLDADTYAK
LSEIAPTVAQTGSEAFFEPWKDQATIIGQAVFKNAEMTELIKSVDDRFTTVKTDHPQFSGKKALLLGG
TLYRGGVQATPPGWRTDFLTQMGLTVLQVPALIPRDEIASVLDGADVLIWTTESDQDRDALLADPIVA
QLAATRRDRNIFTTKELAGAIAFASPLSYPVVADQLPPELARVLG
