# Supplementary material for: Differentiating the roles of Mycobacterium tuberculosis substrate binding proteins, FecB and FecB2, in iron uptake
Source: PLoS Pathog. 2023 Sep 25;19(9):e1011650. doi: 10.1371/journal.ppat.1011650 (PMC10553834; doi:10.1371/journal.ppat.1011650)
Supplement: S1 Table — (DOCX) [file ppat.1011650.s015.docx]

**Supplemental Table 1** DALI server structural homology results for FecB and FecB2

| Search input | Structural homolog | Sequence identify (%) | PDB ID code | Z-score | rmsd, Å | Refer-ence |
| --- | --- | --- | --- | --- | --- | --- |
| FecB | *B. cereus* YfiY | 23 | 3TNY | 32.8 | 2.5* |  |
|  | *S. aureus* SirA | 21 | 3MWF | 29.8 | 3.1 |  |
|  | *S. aureus* HtsA | 20 | 3EIW | 26.1 | 3.6 | [1] |
|  | *B. subtilis* YclQ | 17 | 3GFV | 25.2 | 3.2 | [2] |
|  | *B. anthracis* FpuA | 16 | 6ALL | 24.6 | 3.6 |  |
|  | *B. subtilis* FeuA | 15 | 2WHY | 24.0 | 3.5 | [3] |
|  | *M. smegmatis* FecB2 | 19 | 4MDY | 23.1 | 3.9 |  |
|  | *B. subtilis* FhuD-like | 15 | 3HXP | 22.9 | 3.7 |  |
|  | *Streptococcus pneumoniae* PiaA | 16 | 4HMO | 22.7 | 3.2 | [4] |
|  | *::*  *S. aureus* IsdE | 17 | 2Q8Q | 20.1 | 4.6 | [5] |
|  | *Shigella dysenteriae* ShuT | 21 | 2R7A | 20.4 | 3.8 | [6] |
|  | *Vibro cholerae* HutB | 15 | 5KHL | 18.8 | 3.4 |  |
|  |  |  |  |  |  |  |
| FecB2 | *M. smegmatis* FecB2 | 65 | 4MDY | 42.9 | 0.9 |  |
|  | *S. pneumoniae* PiaA | 22 | 4HMO | 27.5 | 3.8 | [4] |
|  | *B. cereus* YfiY | 21 | 3TNY | 25.0 | 3.0 |  |
|  | *S. aureus* HtsA | 20 | 3EIW | 24.3 | 3.1 | [1] |
|  | *S. aureus* SirA | 22 | 3MWF | 24.2 | 3.9 |  |
|  | *B. anthracis* FpuA | 18 | 6ALL | 22.8 | 3.5 |  |
|  | *E. coli* FepB | 19 | 3TLK | 22.7 | 3.5 |  |
|  | *S. aureus* FhuD2 | 15 | 4FNA | 22.7 | 3.8 | [7] |
|  | *S. pseudintermedius* FhuD | 16 | 5FLY | 22.6 | 3.4 | [8] |
|  | *E. coli* FitE  *::* | 19 | 3BE5 | 22.5 | 3.7 | [9] |
|  | *S. dysenteriae* ShuT  *V. cholerae* HutB  *S. aureus* IsdE | 17  15  10 | 2R7A  5KHL  2Q8P | 17.9  16.8  16.6 | 3.6  3.0  3.3 | [6]  [5] |

rmsd, root-mean-square-deviation.

*Aligned in pymol by cealign to determine rmsd

:: below are the top results for heme-binding PBPs.

1. Beasley, F.C., et al., *Characterization of staphyloferrin A biosynthetic and transport mutants in Staphylococcus aureus.* Molecular Microbiology, 2009. **72**(4): p. 947-963.

2. Zawadzka, A.M., et al., *Characterization of a Bacillus subtilis transporter for petrobactin, an anthrax stealth siderophore.* Proceedings of the National Academy of Sciences of the United States of America, 2009. **106**(51): p. 21854-21859.

3. Peuckert, F., et al., *Structural basis and stereochemistry of triscatecholate siderophore binding by FeuA.* Angewandte Chemie - International Edition, 2009. **48**(42): p. 7924-7927.

4. Cheng, W., et al., *Structures of Streptococcus pneumoniae PiaA and Its Complex with Ferrichrome Reveal Insights into the Substrate Binding and Release of High Affinity Iron Transporters.* PLoS ONE, 2013. **8**(8).

5. Grigg, J.C., et al., *Heme coordination by Staphylococcus aureus IsdE.* Journal of Biological Chemistry, 2007. **282**(39): p. 28815-28822.

6. Ho, W.W., et al., *Holo- and apo-bound structures of bacterial periplasmic heme-binding proteins.* Journal of Biological Chemistry, 2007. **282**(49): p. 35796-35802.

7. Podkowa, K.J., et al., *Crystal and solution structure analysis of FhuD2 from Staphylococcus aureus in multiple Unliganded conformations and bound to ferrioxamine-B.* Biochemistry, 2014. **53**(12): p. 2017-2031.

8. Abate, F., et al., *Crystal structure of FhuD at 1.6% resolution: A ferrichrome-binding protein from the animal and human pathogen Staphylococcus pseudintermedius.* Acta Crystallographica Section:F Structural Biology Communications, 2016. **72**: p. 214-219.

9. Shi, R., et al., *Trapping open and closed forms of FitE - A group III periplasmic binding protein.* Proteins: Structure, Function and Bioinformatics, 2009. **75**(3): p. 598-609.
