# Supplementary material for: Differentiating the roles of Mycobacterium tuberculosis substrate binding proteins, FecB and FecB2, in iron uptake
Source: PLoS Pathog. 2023 Sep 25;19(9):e1011650. doi: 10.1371/journal.ppat.1011650 (PMC10553834; doi:10.1371/journal.ppat.1011650)
Supplement: S2 Table — (DOCX) [file ppat.1011650.s016.docx]

**S2 Table**

**Quantification of Secondary Structure Elements from Circular Dichroism with FecB variants.**

Analysis of CD experiments from **Supplemental Figure 7** provided estimations of secondary structure content, indicating that WT-FecB secondary structure is maintained for the FecB variants. Secondary structure analysis was performed with the BeStSel tool (https://bestsel.elte.hu/index.php).

| Secondary Structure | WT | L135R | R141S | Q233S | R240S | Y242S | Y270S | E272S | D332S | Q336S | E339S | E339S/  R240S | E339S/  Y242S |
| --- | --- | --- | --- | --- | --- | --- | --- | --- | --- | --- | --- | --- | --- |
| % α-helix | 17.7 | 13 | 17.1 | 13 | 13.6 | 17.7 | 14.9 | 18.9 | 12 | 15 | 16.3 | 16.6 | 15.3 |
| % β-sheet | 27.9 | 22.8 | 25.5 | 30.3 | 25.5 | 26.1 | 22.1 | 27.9 | 30.1 | 31.9 | 25.7 | 27.9 | 28.2 |
| % Turn | 13.8 | 15.7 | 12.9 | 14.2 | 14.5 | 13.1 | 14.8 | 12.8 | 14.7 | 14.1 | 14.9 | 13.9 | 14.1 |
| % Other | 40.5 | 48.6 | 44.5 | 42.5 | 46.4 | 43.2 | 48.3 | 40.3 | 43.2 | 39 | 43.2 | 41.5 | 47.5 |
