# Supplementary material for: Differentiating the roles of Mycobacterium tuberculosis substrate binding proteins, FecB and FecB2, in iron uptake
Source: PLoS Pathog. 2023 Sep 25;19(9):e1011650. doi: 10.1371/journal.ppat.1011650 (PMC10553834; doi:10.1371/journal.ppat.1011650)
Supplement: S3 Table — (DOCX) [file ppat.1011650.s017.docx]

**S3 Table**

List of primer sequences used in this study.

| Oligo Name | Sequence **(5' to 3')** |
| --- | --- |
| Rv0265c-For | TGCGGACTTTCCATGGCGGCGG TAACTATCAC |
| Rv0265c-Rev | GTAGTCACTCGAGTGCGCCCAAGATCTG |
| Rv0265c-Thrombin-For | GGGCATATGGCGGCGGTAACTATCACCCACCTGTTCGG |
| Rv0265c-Thrombin-Rev | GGGAAGCTTTCATGCGCCCAAGATCTGGC TGATCTGTGGCG |
| Rv3044-For | GGCATATGGCGTCACAATCGATGATCACGCCCACCACCC |
| Rv3044-Rev | GGAAGCTTCTAGTTGATCGGCGCGTCGACCCAGCG |
| R141S-FecB-For | ATCTGCCCGGTGTCGGTACTTCCAGCGCCCCCGACCT |
| R141S-FecB-Rev | AGGTCGGGGGCGCTGGAAGTACCGACACCGGGCAGAT |
| Q233S-FecB-For | GTCGATCGTGAGTCTGACCGCCAACACCAT |
| Q233S-FecB-Rev | ATGGTGTTGGCGGTCAGACTCACGATCGAC |
| R240S-FecB-For | TGACCGCCAACACCATGTCGGTATACGGCGCCAACAACTT |
| R240S-FecB-Rev | AAGTTGTTGGCGCCGTATACCGACATGGTGTTGGCGGTCA |
| Y242S-FecB-For | AACACCATGCGGGTATCCGGCGCCAACAACTTCC |
| Y242S-FecB-Rev | GGAAGTTGTTGGCGCCGGATACCCGCATGGTGTT |
| Y270S-FecB-For | TTCACCGACAAGGCCTCCATCGAGATCGGCACCA |
| Y270S-FecB-Rev | TGGTGCCGATCTCGATGGAGGCCTTGTCGGTGAA |
| E272S-FecB-For | ACCGACAAGGCCTACATCTCGATCGGCACCA |
| E272S-FecB-Rev | TGGTGCCGATCGAGATGTAGGCCTTGTCGGT |
| D322S-FecB-For | TCTTCGTCGTCAACAGCCAGGTATGGCAGACCG |
| D322S-FecB-Rev | CGGTCTGCCATACCTGGCTGTTGACGACGAAGA |
| Q336S-FecB-For | AACGACCAGGTATGGAGTACCGGCGAGGGTATGGT |
| Q336S-FecB-Rev | ACCATACCCTCGCCGGTACTCCATACCTGGTCGTT |
| E339S-FecB-For | TATGGCAGACCGGCTCAGGTATGGTCGCTG |
| E339S-FecB-Rev | CAGCGACCATACCTGAGCCGGTCTGCCATA |
| Y39S-FecB2-For | AGCGCGTGGTCAGCGCCGGCTCCACCGAGCAGGACGACTT |
| Y39S-FecB2-Rev | AAGTCGTCCTGCTCGGTGGAGCCGGCGCTGACCACGCGCT |
| W58S-FecB2-For | CCCATCGCGGTGACCGACTCGTTCGGTGACCAGCCGTTTG |
| W58S-FecB2-Rev | CAAACGGCTGGTCACCGAACGAGTCGGTCACCGCGATGGG |
| R184S-FecB2-For | TGTTGCTGCAGGGTTCCCTCTGGCAGGGCAACGT |
| R184S-FecB2-Rev | ACGTTGCCCTGCCAGAGGGAACCCTGCAGCAACA |
| S2334-attB2-RBS-MSM-FecB1-For | GGGGACAGCTTTCTTGTACAAAGTGGCCAGAAAGGAGGAAGGAGTGCTGACCTTCCGACCG |
| S2335-FLAG-Gly-MSM-fecB1-Rev1 | CTTGTCGTCGTCGTCCTTGTAGTCCTGGAAGTACAGGTTCTCGCCGCCGCCGCCGTTGATCGGTGCGTTGACCC |
| S2336-attB2-RBS-MSM-fecB2-For | GGGGACAGCTTTCTTGTACAAAGTGGCCAGAAAGGAGGAAGGAGTGCCGAGACCACTGACCC |
| S2337-FLAG-Gly-MSM-fecB2-Rev1 | CTTGTCGTCGTCGTCCTTGTAGTCCTGGAAGTACAGGTTCTCGCCGCCGCCGCCTCCCAGCACGCGGGC |
| S2338-attB3-FLAG-TEV-Rev2 | GGGGACAACTTTGTATAATAAAGTTGCCTATCACTTGTCGTCGTCGTCCTTGTAGTCCTGG |
| S2420-Pac-Nde-top | TAACTTAAGACCGGTAAGCTTGATATCGGCGCCTGATGCGGTATTTTCTCCTTACGCATCTGTGCGGTATTTCACACCGCA |
| S2421-Pac-Nde-RC | TATGCGGTGTGAAATACCGCACAGATGCGTAAGGAGAAAATACCGCATCAGGCGCCGATATCAAGCTTACCGGTCTTAAGTTAAT |
| S3423-RBS-MSMEG_0226-Nde-For-Gibson | GTGGCCAGAAAGGAGGAAGGACATATGAAGCTGTTGAAGCGGTTCTGG |
| S3424-MSMEG_0226-EcoRV-Rev-Gibson | CTCGCCGCCGCCACGCGTGATATCTGCAGATTTCACCAGGCAGAAG |
| S3463-Nde-EcoRV_top | TATGCACGTGAGTACTGCGATCGCCTACGTATCTAGAGAT |
| S3464-Nde-EcoRV RC | ATCTCTAGATACGTAGGCGATCGCAGTACTCACGTGCA |
