# Supplementary material for: Differentiating the roles of Mycobacterium tuberculosis substrate binding proteins, FecB and FecB2, in iron uptake
Source: PLoS Pathog. 2023 Sep 25;19(9):e1011650. doi: 10.1371/journal.ppat.1011650 (PMC10553834; doi:10.1371/journal.ppat.1011650)
Supplement: S4 Table — (DOCX) [file ppat.1011650.s018.docx]

**S4 Table**

List of plasmids used in this study

| Plasmids | Description | Antibiotic marker | Source |
| --- | --- | --- | --- |
| pDE43-MEK | Mycobacterial expression vector | Kan^R^ | [1] |
| pKP948 | MEK-Ptb38-RBS-*fecB1* (MSMEG_2319)-FLAG. Used for the expression of *M. smegmatis* FecB1. | Kan^R^ | This study |
| pKP950 | MEK-Ptb38-RBS-*fecB2* (MSMEG_0438)-FLAG. Used for the expression of *M. smegmatis* FecB1 | Kan^R^ | This study |
| pKP991 (control vector) | MEK vector lacking the promoter, ORF and the tag. Created by cloning restriction sites between PacI and NdeI sites in pDE43-MEK. | Kan^R^ | This study |
| pDE43-MEH | Mycobacterial expression vector | Hyg^R^ | [1] |
| pKP1230 | MEH-Ptb38-RBS-MmpS5 (MSMEG_0226)_TEV_FLAG.  Used for the expression of *M. smegmatis* MmpS5. | Hyg^R^ | This study |
| pKP1231 | MEH-Ptb38-RBS-MmpS4 (MSMEG_0380)_TEV_FLAG. Used for the expression of *M. smegmatis* MmpS4. | Hyg^R^ | This study |
| pKP1234 (control vector) | MEH-Ptb38-MCS_TEV_FLAG.  MEK control vector lacking the ORF but carrying the constitutive promoter, multiple cloning sites and the TEV-FLAG tag. | Hyg^R^ | This study |
| pGMEK-P_hsp60_-*fecB*-FLAG | Mycobacterial expression vector for FecB-FLAG | Kan^R^ | This study |
| pGMCS-P_750_-*mmpS5*-HA | Mycobacterial expression vector for MmpS5-HA | Strep^R^ | This study |

1. Blumenthal, A., et al., *Simultaneous analysis of multiple Mycobacterium tuberculosis knockdown mutants in vitro and in vivo.* PLoS One, 2010. **5**(12): p. e15667.
